# Premature Death, Suicide, and Nonlethal Intentional Self-Harm After Psychiatric Discharge

**DOI:** 10.1001/jamanetworkopen.2024.17131

**Published:** 2024-06-26

**Authors:** Philippe Mortier, Susana Conde, Itxaso Alayo, Franco Amigo, Laura Ballester, Roser Cirici Amell, Daniel Guinart, Salvatore Fabrizio Contaldo, Montserrat Ferrer, Angela Leis, Miguel Angel Mayer, Ana Portillo-Van Diest, Beatriz Puértolas-Gracia, Juan Manuel Ramírez-Anguita, Carlos Peña-Salazar, Ferran Sanz, Ronald C. Kessler, Diego Palao, Víctor Pérez Sola, Lars Mehlum, Ping Qin, Gemma Vilagut, Jordi Alonso

**Affiliations:** 1 Health Services Research Group, Hospital del Mar Research Institute, Barcelona, Spain; 2 Centro de Investigación Biomédica en Red de Epidemiología y Salud Pública, Instituto de Salud Carlos III (CIBERESP, ISCIII), Madrid, Spain; 3 Biosistemak Institute for Health Systems Research, Barakaldo, Bizkaia, Spain; 4 Institute of Neuropsychiatry and Addictions (INAD), Parc de Salut Mar, Barcelona, Spain; 5 Centro de Investigación Biomédica en Red de Salud Mental, Instituto de Salud Carlos III (CIBERSAM, ISCIII), Madrid, Spain; 6 Mental Health Research Group, Hospital del Mar Research Institute, Barcelona, Spain; 7 Department of Psychiatry, the Donald and Barbara Zucker School of Medicine at Hofstra/Northwell, Hempstead, New York; 8 Parc Sanitari Sant Joan de Déu, Sant Boi de Llobregat, Spain; 9 Department of Experimental and Health Sciences, Pompeu Fabra University, Barcelona, Spain; 10 Research Program on Biomedical Informatics (GRIB), Hospital del Mar Research Institute, Barcelona, Spain; 11 Department of Medicine and Life Sciences, Universitat Pompeu Fabra, Barcelona, Spain; 12 Mental Health and Intellectual Disability Services, Parc Sanitari Sant Joan de Déu, Barcelona, Spain; 13 Neurology Department, Parc Sanitari Sant Joan de Déu, Barcelona, Spain; 14 Teaching, Research and Innovation Unit, Parc Sanitari Sant Joan de Déu, Barcelona, Spain; 15 Instituto Nacional de Bioinformatica–ELIXIR-ES (IMPaCT-Data-ISCIII), Barcelona, Spain; 16 Department of Health Care Policy, Harvard Medical School, Boston, Massachusetts; 17 Department of Mental Health, Hospital Universitari Parc Taulí; Institut d’Investigació i Innovació Parc Taulí (I3PT), Unitat de Neurociències Traslacional I3PT-INc Universitat Autònoma de Barcelona, Sabadell, Spain; 18 Department of Psychiatry and Legal Medicine, Universitat Autònoma de Barcelona, Bellaterra, Spain; 19 Department of Paediatrics, Obstetrics and Gynaecology and Preventive Medicine and Public Health Department, Universitat Autònoma de Barcelona (UAB), Bellaterra, Spain; 20 National Centre for Suicide Research and Prevention, Institute of Clinical Medicine, University of Oslo, Oslo, Norway

## Abstract

**Importance:**

There is a need for representative research on serious adverse outcomes following discharge from psychiatric hospitalization.

**Objective:**

To compare rates of premature death, suicide, and nonlethal intentional self-harm after psychiatric discharge with rates in the general population and investigate associations of these outcomes with relevant variables associated with the index psychiatric hospitalization.

**Design, Setting, and Participants:**

This retrospective cohort study included all residents from Catalonia, Spain (7.6 million population), who had psychiatric hospitalizations between January 1, 2014, and December 31, 2018, and were older than 10 years at the index (first) hospitalization. Follow-up was until December 31, 2019. Statistical analysis was performed from December 1, 2022, through April 11, 2024.

**Exposures:**

Socioeconomic status, psychiatric diagnoses, duration of index hospitalization, and number of previous psychiatric hospitalizations.

**Main Outcomes and Measures:**

Postdischarge premature death (ie, all-cause death before age 70 years) and suicide (*International Statistical Classification of Diseases and Related Health Problems, Tenth Revision [ICD-10]* code range X60-X84), identified using mortality data, and postdischarge nonlethal intentional self-harm, identified using electronic health record and self-harm case register data. Standardized mortality ratios (SMRs) compared rates of premature death and suicide between the cohort and the general population. Fully adjusted, multivariable, cause-specific Cox proportional hazards regression models for the 3 outcomes were fitted.

**Results:**

A total of 49 108 patients discharged from psychiatric hospitalization were included (25 833 males [52.6%]; mean [SD] age at discharge, 44.2 [18.2] years). During follow-up, 2260 patients (4.6%) died prematurely, 437 (0.9%) died by suicide, and 4752 (9.7%) had an episode of nonlethal intentional self-harm. The overall SMR for premature death was 7.5 (95% CI, 7.2-7.9). For suicide, SMR was 32.9 (95% CI, 29.9-36.0) overall and was especially high among females (47.6 [95% CI, 40.2-54.9]). In fully adjusted sex-stratified hazard models, postdischarge premature death was associated with cognitive disorders (adjusted hazard ratio [AHR], 2.89 [95% CI, 2.24-3.74] for females; 2.59 [95% CI, 2.17-3.08] for males) and alcohol-related disorders (AHR, 1.41 [95% CI, 1.18-1.70] for females; 1.22 [95% CI, 1.09-1.37] for males). Postdischarge suicide was associated with postdischarge intentional self-harm (AHR, 2.83 [95% CI, 1.97-4.05] for females; 3.29 [95% CI, 2.47-4.40] for males), with depressive disorders (AHR, 2.13 [95% CI, 1.52-2.97]) and adjustment disorders (AHR, 1.94 [95% CI, 1.32-2.83]) among males, and with bipolar disorder among females (AHR, 1.94 [95% CI, 1.21-3.09]). Postdischarge intentional self-harm was associated with index admissions for intentional self-harm (AHR, 1.95 [95% CI, 1.73-2.21] for females; 2.62 [95% CI, 2.20-3.13] for males) as well as for adjustment disorders (AHR, 1.48 [95% CI, 1.33-1.65] for females; 1.99 [95% CI, 1.74-2.27] for males), anxiety disorders (AHR, 1.24 [95% CI, 1.10-1.39] for females; 1.36 [95% CI, 1.18-1.58] for males), depressive disorders (AHR, 1.54 [95% CI, 1.40-1.69] for females; 1.80 [95% CI, 1.58-2.04] for males), and personality disorders (AHR, 1.59 [95% CI, 1.46-1.73] for females; 1.43 [95% CI, 1.28-1.60] for males).

**Conclusions and Relevance:**

In this cohort study of patients discharged from psychiatric hospitalization, risk for premature death and suicide was significantly higher compared with the general population, suggesting individuals discharged from psychiatric inpatient care are a vulnerable population for premature death and suicidal behavior.

## Key Points

**Question:**

What is the risk for premature death, suicide, and nonlethal intentional self-harm following discharge from psychiatric hospitalization?

**Findings:**

In this cohort study including 49 108 patients, risk for postdischarge premature death (age <70 years) and suicide was significantly higher compared with the general population. Premature death was associated with cognitive disorders and alcohol-related disorders in both sexes; suicide was associated with postdischarge nonlethal intentional self-harm in both sexes, with depressive and adjustment disorders in males, and with bipolar disorder in females.

**Meaning:**

The findings suggest individuals discharged from psychiatric inpatient care constitute a vulnerable population for premature death and suicidal behavior.

## Teaser

This cohort study estimates rates of all-cause premature death, suicide, and nonlethal intentional self-harm after discharge from a psychiatric hospitalization among patients in Spain and investigates associations of these outcomes with variables including sex and mental disorders.

## Introduction

Discharge from psychiatric hospitalization carries a heightened risk of serious adverse outcomes^1^; suicide and self-harm rates are particularly elevated shortly after discharge,^2,3,4^ while long-term follow-up reveals a shift toward natural causes of death, especially vascular disease.^2^ Comparing adverse outcome rates and risk factors after psychiatric discharge is challenging due to significant between-study heterogeneity^2,3,5^ and sample restrictions regarding age,^2,4^ gender,^4^ patient type,^2,5^ and type of mental disorder.^2^ Large representative cohort studies encompassing multiple outcomes could address these challenges, but to our knowledge only 1 prior study, focusing on Danish residents, has explored all-cause death, suicide, and self-harm after psychiatric discharge in a single cohort.^1^ That study did not analyze risk based on relevant clinical information associated with the index admission.

We conducted a registry-based cohort study of patients discharged from psychiatric hospitalization to compare rates of premature death (ie, all-cause death before age 70 years) and suicide between the discharged cohort and the general population. Within the study cohort, we investigated the associations of premature death, suicide, and nonlethal intentional self-harm with independent variables including sex, age, socioeconomic status, intentional self-harm at admission and during follow-up, 15 mental disorder categories associated with the index hospitalization, duration of the index hospitalization, and number of previous psychiatric admissions.

## Methods

### Study Cohort

This retrospective cohort study included all patients from Catalonia, Spain (7.6 million population), with psychiatric hospitalizations between January 1, 2014, and December 31, 2018. The first hospitalization for each patient within this time frame was designated as the index hospitalization. We excluded patients aged younger than 10 years at discharge from the index hospitalization, who had missing values for health region, or who died during the index hospitalization. Follow-up for premature death, suicide, and nonlethal intentional self-harm extended until December 31, 2019, to ensure a minimum follow-up period of 1 year, thus mitigating potential inflation of incidence and risk estimates resulting from shorter exposure times.^3^ The observation period (2014-2019) was chosen based on common data availability across the data sources. Patients who emigrated from Catalonia during the observation period were followed up until the date of emigration and then were considered lost to follow-up. The protocol of this study was approved by the Parc de Salut Mar clinical research ethics committee, which waived the requirement for informed consent because all registry data had been fully anonymized and subjected to reidentification risk analysis. This cohort study followed the Strengthening the Reporting of Observational Studies in Epidemiology (STROBE) reporting guideline.

### Sources of Registry Data

Registry data representing the entire Catalan public health care system come from the Catalan Data Analytics Program for Health Research and Innovation.^6^ Catalonia’s health care system provides universal coverage funded through taxation.^7^ Using personal health care identification numbers, we linked registry data from the following 4 sources.

Mortality data came from Spain’s National Statistics Institute (INE),^8^ covering the period 2014 to 2019, and included information on date and cause of death categorized as suicide, other external causes, and natural causes. Determination of cause of death was ascertained using *International Statistical Classification of Diseases and Related Health Problems, Tenth Revision (ICD-10)* codes, aligning with World Health Organization (WHO) criteria,^9^ and relies on medical death certificates and statistical death registers (natural deaths) and judicial statistical death registers (unnatural deaths). The INE follows Eurostat’s European Statistics Code of Practice^10^ for data quality assurance.

Routinely collected electronic health record (EHR) data covering the period 2008 to 2019 came from the Catalan Health Service^11^ and included admission and discharge dates for psychiatric hospitalization along with associated mental disorder and self-harm codes. These codes use the *International Classification of Diseases, Ninth Revision, Clinical Modification (ICD-9-CM)*; *ICD-10*; and *International Statistical Classification of Diseases, Tenth Revision, Clinical Modification (ICD-10-CM)* classification systems. The registries are used for health care quality monitoring and reimbursement, with data quality ensured through verification and systematic checks by health documentalists in the Catalan Health Department.^12^

Data on clinically confirmed self-harm episodes came from the Catalonia Suicide Risk Code (CSRC) program,^13^ an integrated suicide-prevention protocol within the Catalan public health care system (2014-2019 data available). The protocol mandates face-to-face psychiatric evaluations for Catalan residents presenting with self-harm or suicide risk in any public health care setting.

Administrative data came from the Catalan Health Service’s central population register and cover the period 2008 to 2019. These data include sex, age, health region, and socioeconomic group.

### Outcome Variables

The main outcomes were premature death, suicide, and nonlethal intentional self-harm. Premature death was defined as death by any cause before age 70 years, following WHO criteria.^9^ Suicide was defined as death resulting from intentional self-harm (*ICD-10* code range, X60-X84). Nonlethal intentional self-harm was defined using 2 methods: (1) health care visits associated with standard *ICD* external cause codes for self-harm (*ICD-9-CM* E950*-E959* and *ICD-10* or *ICD-10-CM* T14.91, X60*-X84*, Y87.0, and T36*-T65*/T71* [if the fifth or sixth character equaled 2]) in routine EHR data from 4 health care settings (ie, emergency departments, general hospitalizations, psychiatric hospitalizations, and primary care) or (2) episodes with a clinically confirmed method of self-harm in CSRC program data. The first event of nonlethal intentional self-harm (registered in 1 or both of the data sources) was used to code the outcome event.

### Independent Variables and Covariates

Independent variables included (1) sex; (2) age at discharge from index hospitalization; (3) socioeconomic group (ie, actively working or retired with a contributory annual income less than vs more than €18 000 [US$19 440] as well as socioeconomic vulnerability categories); (4) psychiatric diagnosis of nonlethal intentional self-harm associated with index hospitalization (yes or no); (5) at least 1 nonlethal intentional self-harm episode occurring between psychiatric discharge from index hospitalization and suicide or premature death (yes or no); (6) mental disorder categories (not mutually exclusive), created by categorizing mental disorder *ICD* codes associated with the index hospitalization into 15 variables (yes or no) based on the clinical classification software developed by the US Healthcare Cost and Utilization Project (eTable 1 in Supplement 1); (7) duration of index hospitalization; and (8) number of psychiatric hospitalizations up to 6 years prior to the index hospitalization. Covariates included health region and year of discharge from the index hospitalization.

### Statistical Analysis

Descriptive statistics were calculated for the study cohort, and cumulative incidence was estimated for the 3 outcomes, including cumulative incidence function graphs. Age-sex standardized mortality ratios (SMRs) compared rates of premature death (ie, all-cause death before age 70) and suicide between the discharged cohort and the general population, with the expected number of deaths calculated using general population mortality rates in Catalonia.^8^ Incidence rates (per 100 000 person-years [PYs]) for the 3 outcomes were estimated as well as the lethality index of postdischarge intentional self-harm (ie, the ratio of postdischarge self-harm to suicide incidence), with a lower index indicating higher lethality.

Fully adjusted, multivariable, cause-specific Cox proportional hazards regression models for the 3 outcomes were fitted, considering all individual variables and covariates. As there was evidence for violation of the proportional hazards assumption in some models, models were repeated for different postdischarge follow-up times (7 days, 1 month, 3 months, 1 year, and 5 years). Multiple testing was controlled for false discovery rate (FDR) on a model-by-model basis, with FDR set at 5% using the Benjamini-Hochberg procedure.^14^

Death by any cause other than suicide was a competing event in all suicide analyses, and death by any cause was a competing event in all intentional self-harm analyses. All analyses were conducted in the full cohort and stratified by sex. Premature death analyses were restricted to the subcohort aged 10 to 69 years at discharge, with follow-up time censored at age 70 years.

R, version 4.3.1 (R Project for Statistical Computing) was used for the analysis, including R packages survival (version 3.5), cmprsk (version 2.2), tidycmprsk (version 0.2), and ggsurvfit (version 0.3). Statistical analysis was performed from December 1, 2022, through April 11, 2024. Two-sided *P* < .05 was considered significant.

## Results

### Study Cohort Descriptive Statistics

Of 49 527 total patients with psychiatric hospitalizations from 2014 to 2018, we excluded 297 aged younger than 10 years, 8 with missing values for health region, and 114 who died during the index hospitalization. Included in the final cohort were 49 108 patients, with the first hospitalization in this period selected as the index hospitalization for each patient; 44 267 of these were aged 10 to 69 years at discharge. Median follow-up time after discharge was 1327 (IQR, 832-1799) days; 1254 patients (2.6%) emigrated from Catalonia before December 31, 2019 (ie, were lost to follow-up). We used single imputation for 3 patients (<0.1%) with missing age values. The cohort comprised 23 275 females (47.4%) and 25 833 males (52.6%), with a mean (SD) age at discharge from index hospitalization of 44.2 (18.2) years. During follow-up, 2260 patients (4.6%) died prematurely (ie, before age 70 years), 437 (0.9%) died by suicide, and 4752 (9.7%) had 1 or more episodes of nonlethal intentional self-harm (3767 [7.7%] identified in routine EHR data and an additional 985 [2.0%] identified in CSRC program data). Notably, 11 957 patients (24.3%) had prior hospitalizations in the 6 years before index admission. The most prevalent mental disorders diagnosed during index hospitalization were schizophrenia and other psychotic disorders (15 629 [31.8%]), drug-related disorders (14 089 [28.7%]), alcohol-related disorders (11 949 [24.3%]), depressive disorders (9277 [18.9%]), and personality disorders (8246 [16.8%]). Median (IQR) duration of index hospitalization was 15 (7-28) days. See eTable 2 in Supplement 1 for additional study cohort descriptive statistics.

### Cumulative Incidence Estimates

The Figure illustrates the cumulative incidence of the outcomes under study. At 5-year follow-up, cumulative incidence estimates for premature death were 4.8% (95% CI, 4.4%-5.1%) for females and 8.1% (95% CI, 7.7%-8.6%) for males; for suicide were 0.9% (95% CI, 0.7%-1.0%) for females and 1.3% (95% CI, 1.1%-1.4%) for males; and for intentional self-harm were 14.2% (95% CI, 13.7%-14.7%) for females and 8.7% (95% CI, 8.3%-9.1%) for males (eTable 3 in Supplement 1).

**Figure.**
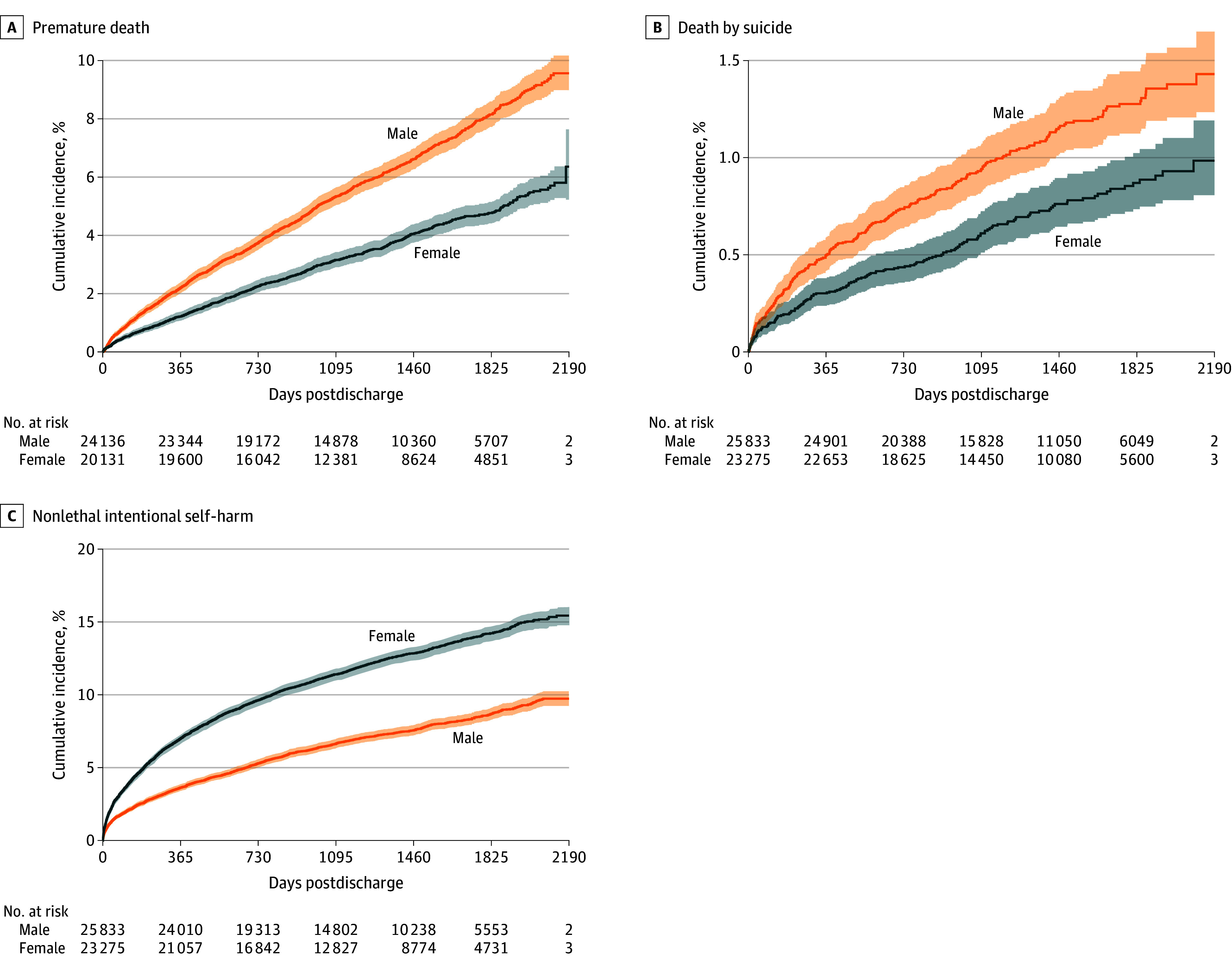
Cumulative Incidence of Postdischarge Serious Adverse Outcomes Premature death analyses were restricted to the subcohort of 44 267 patients aged 69 years or younger at discharge, with follow-up time censored at age 70 years. Shaded areas around each curve indicate 95% CIs.

### Standardized Mortality Ratio Estimates

The age-sex SMRs for premature death were estimated at 7.5 (95% CI, 7.2-7.9) overall, 7.7 (95% CI, 7.2-8.3) for females, and 7.5 (95% CI, 7.1-7.8) for males (eTable 4 in Supplement 1). For suicide, the SMRs were notably higher at 32.9 (95% CI, 29.9-36.0) overall, 47.6 (95% CI, 40.2-54.9) for females, and 27.9 (95% CI, 24.6-31.2) for males (eTable 5 in Supplement 1). Restricting follow-up to the first year postdischarge, SMRs for premature death increased to 9.4 (95% CI, 8.7-10.0) overall, 9.2 (95% CI, 8.0-10.3) for females, and 9.4 (95% CI, 8.6-10.3) for males. For suicide, the SMRs increased to 54.2 (95% CI, 46.7-61.8) overall, 74.7 (95% CI, 57.2-92.2) for females, and 47.2 (95% CI, 39.0-55.4) for males.

### Incidence Rates

The estimated incidence rate for premature death overall was 1435 per 100 000 PYs (95% CI, 1376-1495 per 100 000 PYs); for suicide overall was 250 per 100 000 PYs (95% CI, 227-274 per 100 000 PYs); and for nonlethal intentional self-harm overall was 2925 per 100 000 PYs (95% CI, 2842-3009 per 100 000 PYs). Incidence rates for premature death were estimated at 1040 (95% CI, 965-1114) per 100 000 PYs for females and 1766 (95% CI, 1677-1855) per 100 000 PYs for males; for suicide, 195 (95% CI, 165-225) per 100 000 PYs for females and 301 (95% CI, 265-337) per 100 000 PYs for males; and for intentional self-harm, 3788 (95% CI, 3649-3927) per 100 000 PYs for females and 2175 (95% CI, 2077-2273) per 100 000 PYs for males (Table 1, Table 2, and Table 3; eTables 6-8 in Supplement 1). Lethality was higher among males (lethality index, 7.2 [95% CI, 6.3-8.1], vs 19.4 [95% CI, 16.4-22.5] among females), increased with age, and was notably elevated among individuals with index admissions for bipolar disorder (lethality index, 6.4 [95% CI, 4.8-8.0]) or schizophrenia or other psychotic disorders (lethality index, 6.1 [95% CI, 4.9-7.2]) (eTable 9 in Supplement 1).

**Table 1.** IRs and AHRs for Premature Death After Discharge From Psychiatric Hospitalization^a^

Independent variable	Female patients (n = 20 131)			Male patients (n = 24 136)		
	IR (95% CI) per 100 000 PYs	AHR (95% CI)^b^	*P* value^c^	IR (95% CI) per 100 000 PYs	AHR (95% CI)^b^	*P* value^c^
Age at discharge from index hospitalization, y^d^						
10-14	212 (81-344)	0.35 (0.20-0.62)	.002	121 (33-310)	0.14 (0.06-0.34)	<.001
15-19	103 (41-212)	0.18 (0.09-0.34)	<.001	438 (263-613)	0.51 (0.35-0.74)	.002
20-29	370 (233-507)	0.61 (0.43-0.87)	.03	610 (481-739)	0.64 (0.50-0.81)	.002
30-39	718 (576-860)	1.17 (0.92-1.48)	.42	1110 (966-1253)	1.07 (0.88-1.31)	.70
40-49	1006 (860-1152)	1.68 (1.37-2.07)	<.001	1895 (1715-2074)	1.73 (1.44-2.08)	<.001
50-59	1589 (1381-1797)	2.78 (2.27-3.41)	<.001	2922 (2645-3198)	2.50 (2.07-3.01)	<.001
60-69	2940 (2528-3353)	4.83 (3.88-6.00)	<.001	6219 (5535-6904)	4.66 (3.82-5.68)	<.001
Socioeconomic group^d^						
Contributory annual income <€18 000^e^	986 (897-1075)	0.99 (0.90-1.10)	.89	1764 (1658-1871)	1.01 (0.94-1.09)	.83
Contributory annual income >€18 000^e^	955 (765-1145)	0.89 (0.77-1.04)	.31	1840 (1558-2123)	0.93 (0.83-1.05)	.39
Socioeconomically vulnerable categories	1288 (1098-1477)	1.13 (0.99-1.28)	.18	1732 (1534-1931)	1.06 (0.96-1.17)	.39
Diagnosis of intentional self-harm associated with index hospitalization, yes vs no	776 (483-1068)	1.13 (0.76-1.67)	.70	1822 (1243-2401)	0.97 (0.70-1.36)	.91
Intentional self-harm after discharge from index psychiatric hospitalization, yes vs no	925 (742-1108)	1.03 (0.82-1.28)	.89	2107 (1767-2446)	1.19 (1.00-1.42)	.13
Mental disorders associated with index hospitalization						
Adjustment disorders	740 (564-915)	0.85 (0.64-1.12)	.47	1795 (1484-2106)	0.96 (0.79-1.16)	.77
Alcohol-related disorders	1594 (1366-1822)	1.41 (1.18-1.70)	.002	2320 (2146-2495)	1.22 (1.09-1.37)	.002
Anxiety disorders	789 (575-1003)	0.83 (0.62-1.11)	.43	1493 (1200-1785)	0.84 (0.68-1.03)	.20
Attention-deficit/hyperactivity disorder	444 (144-1037)	1.48 (0.60-3.66)	.60	547 (261-834)	1.00 (0.58-1.71)	.99
Bipolar disorders	1086 (895-1277)	0.75 (0.59-0.96)	.07	1577 (1330-1825)	0.75 (0.62-0.91)	.01
Conduct disorder or oppositional defiant disorder	564 (269-860)	1.19 (0.68-2.06)	.69	632 (348-916)	0.76 (0.48-1.21)	.39
Delirium, dementia, and amnestic and other cognitive disorders	5417 (4183-6652)	2.89 (2.24-3.74)	<.001	8156 (6904-9408)	2.59 (2.17-3.08)	<.001
Depressive disorders	1143 (976-1310)	0.77 (0.63-0.95)	.05	2622 (2308-2936)	1.03 (0.88-1.19)	.82
Developmental disorders	759 (434-1084)	0.96 (0.62-1.49)	.89	959 (654-1264)	0.85 (0.61-1.18)	.48
Disorders usually diagnosed in infancy, childhood, or adolescence	131 (3-732)	0.42 (0.06-3.02)	.60	303 (122-625)	0.49 (0.23-1.04)	.15
Eating disorders	482 (285-678)	1.17 (0.76-1.81)	.67	1145 (371-2672)	1.32 (0.54-3.18)	.73
Personality disorders	944 (792-1097)	0.89 (0.74-1.08)	.47	1711 (1489-1933)	0.94 (0.81-1.09)	.59
Schizophrenia and other psychotic disorders	1021 (882-1160)	0.79 (0.64-0.98)	.10	1321 (1199-1444)	0.73 (0.63-0.84)	<.001
Drug-related disorders	1131 (954-1307)	1.23 (1.01-1.49)	.13	1409 (1287-1531)	0.89 (0.79-1.01)	.15
Other disorders	1020 (642-1398)	1.03 (0.70-1.51)	.89	1635 (1232-2039)	0.95 (0.73-1.22)	.77
Duration of index hospitalization, d^d^						
0-6	1036 (880-1192)	1.14 (0.99-1.32)	.21	2148 (1927-2370)	1.17 (1.06-1.29)	.009
7-13	857 (706-1008)	0.84 (0.72-0.98)	.10	1802 (1614-1989)	1.00 (0.91-1.11)	.99
14-20	1011 (840-1183)	0.98 (0.84-1.14)	.89	1634 (1437-1831)	0.97 (0.87-1.08)	.75
21-27	1109 (888-1330)	1.04 (0.88-1.24)	.79	1448 (1220-1676)	0.91 (0.79-1.04)	.28
≥28	1170 (1016-1324)	1.02 (0.90-1.17)	.86	1695 (1525-1865)	0.97 (0.88-1.07)	.72
Psychiatric hospitalizations in the previous 6 y, No.						
0	948 (863-1033)	1 [Reference]	NA	1699 (1593-1805)	1 [Reference]	NA
1	1137 (925-1348)	1.10 (0.89-1.36)	.60	1860 (1625-2095)	1.13 (0.98-1.31)	.20
2	1620 (1246-1994)	1.64 (1.26-2.11)	.002	1769 (1431-2108)	1.15 (0.93-1.42)	.33
3	927 (548-1306)	0.91 (0.59-1.39)	.79	2178 (1672-2685)	1.38 (1.07-1.76)	.04
4	757 (329-1185)	0.77 (0.43-1.38)	.60	2089 (1449-2728)	1.43 (1.04-1.97)	.08
≥5	1653 (1191-2116)	1.75 (1.28-2.40)	.003	1954 (1500-2409)	1.35 (1.05-1.74)	.06

Abbreviations: AHR, adjusted hazard ratio; IR, incidence rate; PYs, person years.

^a^ All premature death analyses were restricted to the subcohort of 44 267 patients with age at discharge 69 years or less, with follow-up time censored at age 70 years. Median (IQR) age at discharge among those with postdischarge premature death was 51.5 (42.1-59.0) years, median (IQR) age at event was 52.4 (44.0-61.4) years, and median (IQR) survival time (ie, between discharge and event) was 600 (242-1075) days. The IR for postdischarge premature death among females was 1040 (95% CI, 965-1114) per 100 000 PYs and among males was 1766 (95% CI, 1677-1855) per 100 000 PYs (eTable 6 in Supplement 1). The fully AHR for sex (males compared with females) was 1.73 (95% CI, 1.57-1.90; *P* < .001) (eTable 10 in Supplement 2).

^b^ The AHRs were calculated using a multivariable cause-specific hazard model that included all independent variables shown in the table, additionally adjusting for health region and year of discharge from index hospitalization.

^c^ After applying false-discovery-rate (Benjamini-Hochberg) correction for multiple testing.

^d^ Effect coding was used to estimate the deviation of risk (hazard) for all separate variable levels from the mean risk (hazard) in the study cohort.

^e^ US$19 440.

**Table 2.** IRs and AHRs for Suicide After Discharge From Psychiatric Hospitalization^a^

Independent variable	Female patients (n = 23 275)			Male patients (n = 25 833)		
	IR (95% CI) per 100 000 PYs	AHR (95% CI)^b^	*P* value^c^	IR (95% CI) per 100 000 PYs	AHR (95% CI)^b^	*P* value^c^
Age at discharge from index hospitalization, y^d^						
10-14	106 (34-248)	0.52 (0.22-1.22)	.49	121 (33-310)	0.51 (0.20-1.29)	.57
15-19	44 (9-129)	0.23 (0.08-0.66)	.06	201 (82-319)	0.84 (0.48-1.46)	.87
20-29	185 (88-282)	1.22 (0.72-2.05)	.76	234 (154-314)	1.01 (0.70-1.45)	.99
30-39	256 (171-341)	1.63 (1.11-2.40)	.07	262 (192-331)	1.08 (0.79-1.48)	.92
40-49	198 (133-263)	1.28 (0.87-1.86)	.62	365 (286-444)	1.31 (1.00-1.73)	.26
50-59	270 (184-355)	1.89 (1.30-2.76)	.02	354 (258-450)	1.15 (0.84-1.57)	.72
60-69	252 (147-358)	1.87 (1.19-2.93)	.06	394 (240-549)	1.30 (0.87-1.94)	.60
≥70	96 (44-182)	0.91 (0.47-1.74)	.86	322 (153-491)	1.09 (0.65-1.85)	.94
Socioeconomic group^d^						
Contributory annual income <€18 000^e^	186 (150-221)	0.95 (0.76-1.18)	.79	305 (262-347)	1.01 (0.84-1.20)	.99
Contributory annual income >€18 000^e^	208 (126-289)	1.09 (0.81-1.48)	.79	433 (305-561)	1.35 (1.06-1.72)	.15
Socioeconomically vulnerable categories	219 (143-294)	0.97 (0.73-1.29)	.88	209 (141-278)	0.74 (0.57-0.95)	.15
Diagnosis of intentional self-harm associated with index hospitalization, yes vs no	445 (234-657)	2.50 (1.45-4.32)	.02	620 (295-945)	1.22 (0.70-2.14)	.82
Intentional self-harm after discharge from index psychiatric hospitalization, yes vs no	458 (332-583)	2.83 (1.97-4.05)	<.001	934 (712-1156)	3.29 (2.47-4.40)	<.001
Mental disorders associated with index hospitalization						
Adjustment disorders	246 (148-345)	1.55 (0.93-2.57)	.38	546 (379-713)	1.94 (1.32-2.83)	.01
Alcohol-related disorders	253 (164-342)	1.13 (0.74-1.74)	.79	316 (253-380)	1.05 (0.79-1.37)	.94
Anxiety disorders	262 (144-380)	1.60 (0.97-2.63)	.33	375 (231-520)	1.25 (0.82-1.90)	.63
Attention-deficit/hyperactivity disorder	266 (55-778)	2.36 (0.72-7.76)	.50	195 (63-455)	1.17 (0.46-2.96)	.94
Bipolar disorders	306 (211-400)	1.94 (1.21-3.09)	.06	281 (180-382)	1.20 (0.78-1.84)	.74
Conduct disorder or oppositional defiant disorder	112 (23-326)	1.23 (0.37-4.03)	.85	96 (20-279)	0.53 (0.16-1.74)	.63
Delirium, dementia, and amnestic and other cognitive disorders	84 (23-216)	0.64 (0.23-1.81)	.72	188 (75-387)	0.62 (0.28-1.37)	.63
Depressive disorders	196 (135-257)	1.12 (0.72-1.74)	.79	571 (435-706)	2.13 (1.52-2.97)	<.001
Developmental disorders	35 (1-195)	0.20 (0.03-1.48)	.45	124 (40-290)	0.60 (0.24-1.46)	.63
Disorders usually diagnosed in infancy, childhood, or adolescence	131 (3-732)	1.61 (0.22-11.91)	.79	86 (10-312)	0.40 (0.10-1.68)	.60
Eating disorders	167 (72-329)	1.39 (0.65-2.95)	.72	671 (138-1960)	2.90 (0.90-9.31)	.32
Personality disorders	271 (192-350)	1.18 (0.81-1.71)	.72	344 (247-442)	1.01 (0.73-1.39)	.99
Schizophrenia and other psychotic disorders	162 (110-214)	1.12 (0.69-1.80)	.79	262 (209-316)	1.26 (0.90-1.76)	.58
Drug-related disorders	244 (163-325)	1.15 (0.75-1.75)	.79	252 (201-303)	0.82 (0.62-1.09)	.58
Other disorders	156 (51-364)	0.99 (0.40-2.43)	.98	148 (54-323)	0.47 (0.21-1.06)	.32
Duration of index hospitalization, d^d^						
0-6	185 (124-246)	0.91 (0.66-1.26)	.79	337 (252-422)	1.02 (0.80-1.30)	.97
7-13	178 (113-243)	0.90 (0.65-1.24)	.79	354 (273-435)	1.18 (0.95-1.48)	.53
14-20	160 (96-224)	0.81 (0.57-1.15)	.62	269 (191-347)	0.94 (0.73-1.22)	.92
21-27	292 (187-396)	1.54 (1.11-2.13)	.07	281 (183-378)	0.99 (0.74-1.34)	.99
≥28	194 (137-251)	0.99 (0.74-1.32)	.94	261 (196-325)	0.88 (0.70-1.12)	.65
Psychiatric hospitalizations in the previous 6 y, No.						
0	167 (135-200)	1 [Reference]	NA	287 (245-329)	1 [Reference]	NA
1	218 (131-305)	1.19 (0.75-1.89)	.76	289 (198-379)	1.17 (0.81-1.67)	.73
2	369 (199-540)	1.98 (1.17-3.36)	.07	311 (171-451)	1.25 (0.76-2.04)	.72
3	267 (107-550)	1.45 (0.66-3.21)	.72	420 (200-641)	1.76 (1.00-3.09)	.26
4	121 (15-436)	0.63 (0.15-2.62)	.79	651 (297-1004)	2.73 (1.51-4.92)	.01
≥5	335 (137-533)	1.63 (0.83-3.20)	.50	266 (101-430)	0.99 (0.51-1.93)	.99

Abbreviations: AHR, adjusted hazard ratio; IR, incidence rate; NA, not applicable; PYs, person-years.

^a^ Median (IQR) age at discharge among those with postdischarge death by suicide was 44.7 (35.8-55.7) years, median (IQR) age at event was 46.8 (36.9-56.9) years, and median (IQR) survival time (ie, between discharge and event) was 430 (132-976) days. The IR for postdischarge suicide among females was 195 (95% CI, 165-225) per 100 000 PYs and among males was 301 (95% CI, 265-337) per 100 000 PYs (eTable 7 in Supplement 1). The fully AHR for sex (males compared with females) was 1.87 (95% CI, 1.51-2.30; *P* < .001) (eTable 11 in Supplement 1).

^b^ The AHRs were calculated using a multivariable cause-specific hazard model that included all variables shown in the table, additionally adjusting for health region and year of discharge from index hospitalization.

^c^ After applying false-discovery-rate (Benjamini-Hochberg) correction for multiple testing.

^d^ Effect coding was used to estimate the deviation of risk (hazard) for all separate variable levels from the average risk (hazard) in the study cohort.

^e^ US$19 440.

**Table 3.** IRs and HRs for Intentional Self-Harm After Discharge From Psychiatric Hospitalization^a^

Independent variable	Female patients (n = 23 275)			Male patients (n = 25 833)		
	IR (95% CI) per 100 000 PYs	HR (95% CI)^b^	*P* value^c^	IR (95% CI) per 100 000 PYs	HR (95% CI)^b^	*P* value^c^
Age at discharge from index hospitalization, y^d^						
10-14	6912 (6091-7734)	1.59 (1.40-1.80)	<.001	1926 (1443-2410)	0.91 (0.71-1.17)	.60
15-19	6187 (5548-6825)	1.42 (1.28-1.58)	<.001	2213 (1809-2618)	1.17 (0.98-1.40)	.15
20-29	4392 (3895-4889)	1.19 (1.07-1.33)	.004	1898 (1666-2131)	1.10 (0.97-1.25)	.25
30-39	4022 (3668-4375)	1.15 (1.05-1.26)	.006	2264 (2053-2475)	1.21 (1.09-1.35)	.002
40-49	4298 (3979-4617)	1.18 (1.09-1.28)	<.001	2560 (2344-2775)	1.21 (1.09-1.34)	.001
50-59	3129 (2825-3433)	0.88 (0.80-0.97)	.02	2192 (1946-2438)	1.01 (0.90-1.14)	.93
60-69	2536 (2191-2880)	0.76 (0.66-0.86)	<.001	1655 (1333-1978)	0.81 (0.67-0.98)	.07
≥70	1295 (1061-1528)	0.41 (0.34-0.49)	<.001	1542 (1167-1917)	0.71 (0.56-0.90)	.01
Socioeconomic group^d^						
Contributory annual income <€18 000^e^	3701 (3535-3868)	0.94 (0.90-1.00)	.06	2161 (2044-2277)	0.99 (0.93-1.06)	.92
Contributory annual income >€18 000^e^	3643 (3286-3999)	0.93 (0.87-1.01)	.11	2089 (1801-2377)	0.89 (0.81-0.99)	.07
Socioeconomically vulnerable categories	4248 (3896-4600)	1.13 (1.06-1.21)	.001	2279 (2048-2510)	1.13 (1.03-1.23)	.02
Diagnosis of intentional self-harm associated with index hospitalization, yes vs no	12 111 (10 831-13 391)	1.95 (1.73-2.21)	<.001	8059 (6760-9357)	2.62 (2.20-3.13)	<.001
Mental disorders associated with index hospitalization						
Adjustment disorders	7654 (7049-8258)	1.48 (1.33-1.65)	<.001	4860 (4329-5391)	1.99 (1.74-2.27)	<.001
Alcohol-related disorders	4307 (3920-4693)	1.04 (0.93-1.16)	.58	2483 (2301-2666)	1.07 (0.96-1.19)	.30
Anxiety disorders	5728 (5138-6318)	1.24 (1.10-1.39)	<.001	3332 (2887-3778)	1.36 (1.18-1.58)	<.001
Attention-deficit/hyperactivity disorder	5117 (3699-6536)	0.98 (0.74-1.31)	.91	2452 (1831-3072)	1.19 (0.90-1.56)	.33
Bipolar disorders	2069 (1816-2322)	0.64 (0.55-0.74)	<.001	1636 (1388-1883)	0.88 (0.74-1.05)	.25
Conduct disorder or oppositional defiant disorder	4361 (3531-5192)	1.05 (0.86-1.30)	.67	1861 (1374-2349)	0.96 (0.72-1.28)	.90
Delirium, dementia, and amnestic and other cognitive disorders	1105 (801-1408)	0.54 (0.40-0.72)	<.001	1267 (901-1634)	0.69 (0.50-0.94)	.05
Depressive disorders	5358 (5018-5697)	1.54 (1.40-1.69)	<.001	3632 (3275-3988)	1.80 (1.58-2.04)	<.001
Developmental disorders	2687 (2062-3312)	0.72 (0.57-0.92)	.02	1596 (1199-1994)	0.81 (0.63-1.05)	.21
Disorders usually diagnosed in infancy, childhood, or adolescence	4028 (2536-5520)	0.80 (0.55-1.17)	.32	1522 (1010-2033)	0.70 (0.49-1.00)	.11
Eating disorders	5566 (4844-6289)	1.03 (0.89-1.19)	.72	1876 (809-3695)	0.81 (0.40-1.63)	.68
Personality disorders	6823 (6391-7256)	1.59 (1.46-1.73)	<.001	3567 (3240-3894)	1.43 (1.28-1.60)	<.001
Schizophrenia and other psychotic disorders	1481 (1320-1642)	0.44 (0.38-0.51)	<.001	1272 (1152-1392)	0.63 (0.55-0.72)	<.001
Drug-related disorders	4097 (3749-4446)	0.89 (0.80-0.99)	.05	2308 (2148-2468)	1.00 (0.90-1.11)	.96
Other disorders	4005 (3276-4734)	0.94 (0.78-1.13)	.59	2969 (2421-3516)	1.21 (1.00-1.47)	.11
Duration of index hospitalization, d^d^						
0-6	5868 (5497-6238)	1.20 (1.12-1.28)	<.001	3317 (3041-3592)	1.30 (1.20-1.42)	<.001
7-13	3869 (3551-4187)	1.02 (0.95-1.10)	.64	2476 (2255-2697)	1.14 (1.04-1.24)	.01
14-20	3404 (3095-3712)	0.98 (0.91-1.07)	.72	1955 (1740-2169)	0.97 (0.88-1.07)	.62
21-27	2817 (2482-3153)	0.90 (0.82-1.00)	.07	1654 (1413-1894)	0.86 (0.76-0.98)	.05
≥28	2804 (2578-3030)	0.92 (0.85-0.99)	.06	1504 (1347-1661)	0.81 (0.74-0.90)	<.001
Psychiatric hospitalizations in the previous 6 y, No.						
0	3858 (3693-4023)	1 [Reference]	NA	2203 (2084-2322)	1 [Reference]	NA
1	2970 (2635-3305)	1.09 (0.96-1.24)	.22	1733 (1506-1960)	1.04 (0.90-1.20)	.73
2	3801 (3225-4378)	1.44 (1.22-1.70)	<.001	1978 (1616-2339)	1.30 (1.06-1.58)	.03
3	3371 (2632-4109)	1.27 (1.01-1.60)	.07	2209 (1687-2730)	1.57 (1.22-2.01)	.002
4	4224 (3172-5275)	1.63 (1.25-2.11)	<.001	2703 (1954-3453)	1.95 (1.46-2.61)	<.001
≥5	5528 (4652-6404)	1.96 (1.64-2.33)	<.001	3362 (2747-3976)	2.59 (2.11-3.18)	<.001

Abbreviations: HR, hazard ratio; IR, incidence rate, NA, not applicable; PYs, person years.

^a^ Median (IQR) age at discharge among those with postdischarge nonlethal intentional self-harm was 41.1 (27.1-50.3) years, median (IQR) age at event was 42.1 (28.5-51.6) years, and median (IQR) survival time (ie, between discharge and event) was 314 (80-752) days. IR for postdischarge nonlethal intentional self-harm: females, 3788 (95% CI, 3649-3927) per 100 000 PYs; males, 2175 (95% CI, 2077-2273) per 100 000 PYs (eTable 8 in Supplement 1). Fully adjusted HR for sex (females vs males): 1.47 (95% CI, 1.38-1.56; *P* < .001) (eTable 12 in Supplement 1).

^b^ The HRs were calculated using a multivariable cause-specific hazard model that included all variables shown in the table, additionally adjusting for health region and year of discharge from index hospitalization.

^c^ After applying false-discovery-rate (Benjamini-Hochberg) correction for multiple testing.

^d^ Effect coding was used to estimate the deviation of risk (hazard) for all separate variable levels from the average risk (hazard) in the study cohort.

^e^ US$19 440.

### Cause-Specific Hazard Models

In fully adjusted multivariable hazard models (Table 1, Table 2, and Table 3), risk for postdischarge premature death and suicide was significantly higher among males compared with females (premature death: adjusted hazard ratio [AHR], 1.73 [95% CI, 1.57-1.90]; suicide: AHR, 1.87 [95% CI, 1.51-2.30]) and gradually increased with age in both sexes, especially for premature death. In contrast, females compared with males had higher risk for postdischarge intentional self-harm (AHR, 1.47 [95% CI, 1.38-1.56]), and risk gradually decreased with age in both sexes. Risk for intentional self-harm was higher among socioeconomically vulnerable categories in both sexes.

Index admissions for intentional self-harm were associated with postdischarge repeat self-harm, especially among males (females: AHR, 1.95 [95% CI, 1.73-2.21]; males: AHR, 2.62 [95% CI, 2.20-3.13]), and were associated with postdischarge suicide among females (AHR, 2.50 [95% CI, 1.45-4.32]). In addition, incidence of 1 or more intentional self-harm episodes after index admission was associated with subsequent suicide among both males (AHR, 3.29 [95% CI, 2.47-4.40]) and females (AHR, 2.83 [95% CI, 1.97-4.05]) and with premature death among males (AHR, 1.19 [95% CI, 1.00-1.42]).

Risk for postdischarge premature death was especially high among the 3108 individuals with cognitive disorders (6.3%) (females: AHR, 2.89 [95% CI, 2.24-3.74]; males: AHR, 2.59 [95% CI, 2.17-3.08]) followed by alcohol-related disorders (females: AHR, 1.41 [95% CI, 1.18-1.70]; males: AHR, 1.22 [95% CI, 1.09-1.37]) and was significantly lower among both females and males admitted for bipolar disorders (females: AHR, 0.75 [95% CI, 0.59-0.96]; males: AHR, 0.75 [95% CI, 0.62-0.91]) or psychosis (females: AHR, 0.79 [95% CI, 0.64-0.98]; males: AHR, 0.73 [95% CI, 0.63-0.84]) and among females admitted for depressive disorders (AHR, 0.77 [95% CI, 0.63-0.95]). Postdischarge suicide was associated with admissions for depressive disorders (AHR, 2.13 [95% CI, 1.52-2.97]) and adjustment disorders (AHR, 1.94 [95% CI, 1.32-2.83]) among males and with bipolar disorder among females (AHR, 1.94 [95% CI, 1.21-3.09]). Postdischarge intentional self-harm was consistently associated in both sexes with admissions for adjustment disorders (females: AHR, 1.48 [95% CI, 1.33-1.65]; males: AHR, 1.99 [95% CI, 1.74-2.27]), anxiety disorders (females: AHR, 1.24 [95% CI, 1.10-1.39]; males: AHR, 1.36 [95% CI, 1.18-1.58]), depressive disorders (females: AHR, 1.54 [95% CI, 1.40-1.69]; males: AHR, 1.80 [95% CI, 1.58-2.04]), and personality disorders (females: AHR, 1.59 [95% CI, 1.46-1.73]; males: AHR, 1.43 [95% CI, 1.28-1.60]), with AHRs being slightly higher among males.

Having previous psychiatric hospitalizations was associated with risk for all 3 adverse outcomes, with a clear gradual increase of risk for intentional self-harm with an increasing number of previous hospitalizations. Shorter duration of index psychiatric admission was associated with postdischarge intentional self-harm. eTables 10 to 15 in Supplement 1 provide an overview of all multivariable hazard models.

## Discussion

We found that risk of premature death among individuals discharged from psychiatric hospitalization was almost 8 times higher compared with the general population. Prior research has shown increased all-cause mortality posthospitalization,^1,2,15,16^ but these studies did not define premature death as strictly as ours (ie, death before age 70 years). This likely contributes to the lower premature death rate in our study’s cohort (1435 per 100 000 PYs) compared with the all-cause mortality rate of 2414 per 100 000 PYs estimated among Catalan adult patients with mental disorders (with or without psychiatric hospitalizations).^17^ This underscores the necessity for further studies distinguishing between premature and general mortality in discharged individuals, which is crucial for providing evidence to prevent avoidable deaths and address health disparities in this vulnerable population.

Among those discharged from psychiatric hospitalization, we found a higher risk of premature death among the small group of individuals (6.3%) previously admitted for cognitive disorders (AHR, 2.89 for females and 2.59 for males). The underlying pathology leading to cognitive syndromes may explain this, and prevention efforts may be limited to adequate management of this pathology and the superimposed cognitive syndromes. Risk for premature death was also increased in the far larger group (24.3%) of those previously admitted for alcohol-related disorders, in line with prior research.^1,15,16,18,19,20,21,22,23^ This finding emphasizes the importance of targeted prevention interventions for patients with alcohol use disorders, including improved detection and treatment efforts for neoplasms, infectious diseases, diabetes, circulatory system diseases, and respiratory diseases.^17,24,25,26^

Consistent with prior evidence,^5,15,27^ our study revealed substantially elevated postdischarge suicide rates compared with the general population (SMR, 32.9), especially in the first year postdischarge (SMR, 54.2), and these were associated with previous intentional self-harm. Our sex-specific analyses provide a deeper understanding of the association between mental disorders and postdischarge suicide risk. First, among males, our findings aligned with a previous study among Swedish patients^28^ by documenting a specific association of postdischarge suicide with adjustment and depressive disorders. Adjustment disorders are not frequently included in epidemiological mental health studies, and further research is needed to determine their exact role with regard to suicide risk. Second, although postdischarge suicide rates in this study were higher among males compared with females (1.3% vs 0.9%), consistent with an earlier study,^29^ females had disproportionately high suicide rates compared with the general population (SMR, 47.6), in line with previous evidence^30^ and potentially explained by higher treatment-seeking rates among females with severe disorders.^31^ In line with a study among Danish patients,^32^ we found that admission for bipolar disorder was associated with postdischarge suicide among females. Adequate treatment for bipolar disorder, including considering the use of lithium and electroconvulsive therapy,^33^ may contribute to suicide prevention in this particularly vulnerable group.

An important contribution of our study is the estimation of nonlethal intentional self-harm rates after psychiatric hospitalization discharge drawing from reliable EHR data and specific self-harm case registry data. Our estimated rate of 2925 per 100 000 PYs is comparable with rates from Denmark^1^ but substantially higher than the pooled rate of 722 per 100 000 PYs estimated in a 2019 meta-analysis,^3^ most likely due to methodological constraints related to the evidence included in the meta-analysis, such as small sample sizes and selected samples. Our study identified intentional self-harm, adjustment disorders, anxiety disorders, depressive disorders, and personality disorders as independent risk factors associated with postdischarge self-harm. This is in line with a previous study among US psychiatric inpatients that documented an important role of negative affectivity, conscientiousness, neuroticism, and borderline personality traits when explaining postdischarge suicidal behavior.^34^ In line with a previous study from Denmark among patients hospitalized for self-harm,^35^ our findings also indicated heightened lethality of postdischarge self-harm among patients with bipolar disorder and schizophrenia.

A recent comprehensive review of evidence^39^ showed that mechanisms associated with premature death and suicidal behavior among people with mental illness have many commonalities, including behavioral factors (eg, smoking, poor diet, and low physical activity) and reduced access to adequate care. Proposed solutions include policies to cease tobacco use and improve nutrition as well as better integration of mental and physical health care through investment in collaborative care models, training, and early detection and treatment of comorbidity. Precision medicine approaches, through the use of machine learning–based prediction algorithms,^32,40,41^ have shown potential to better delineate patients at highest risk, opening the perspective on improved allocation of effective mental health treatment in this vulnerable population.

### Limitations

Our study has a number of limitations. First, the use of EHR data may be prone to misclassification and underregistration by medical professionals. However, this risk is mitigated in the Catalan public health care system, where professional health documentalists conduct continuous validation of health records, ensuring reliability and standardization of available health information. Second, health information of private health care providers (eg, hospitals, primary health care centers, or emergency departments) was not included in our study. Although access to public health care in Catalonia is universal, about 31.6% (24.9% in 2014) of Catalan residents have both private and public health care coverage.^36^ One US study found an association between low income levels and increased propensity to seek outpatient mental health treatment in the private (vs public) sector,^37^ suggesting that individuals with higher socioeconomic status may be underrepresented in our study. However, private insurance is commonly acquired only to bypass waiting times in Spain’s public health care system,^38^ and since most psychiatric hospitalizations cannot be postponed, individuals with dual health care coverage may be inclined to rely on the public health care system. Third, findings from this study are representative for the autonomous region of Catalonia, Spain, and may not generalize to other regions in Spain or other countries. Fourth, we did not investigate postdischarge repetition of self-harm and we did not have access to the specific causes of premature death in our cohort. Future studies should also investigate the associations of mental disorder comorbidity, time-varying effects of postdischarge repeat self-harm, and postdischarge repeat hospitalizations with serious postdischarge adverse outcomes.

## Conclusions

In this cohort study of patients discharged from psychiatric hospitalization, we observed a significantly higher risk of premature death compared with the general population, particularly pronounced among those previously hospitalized for cognitive disorders and alcohol-related disorders. Furthermore, we identified a significantly elevated risk of suicide and nonlethal intentional self-harm, particularly in postdischarge suicide rates, than was observed in the general population, with specific associated variables being adjustment and depressive disorders among males and bipolar disorders among females.

## Notes

Supplement 1. **eTable 1.** Mental Disorder Diagnosis Categories and Corresponding *ICD-9-CM* and *ICD-10-CM* Diagnostic Codes **eTable 2.** Study Cohort Descriptive Statistics **eTable 3.** Cumulative Incidence Estimates of Adverse Outcomes After Discharge From Psychiatric Hospitalization, Stratified by Postdischarge Follow-Up Time **eTable 4.** Standardized Mortality Ratios for Premature Mortality **eTable 5.** Standardized Mortality Ratios for Suicide **eTable 6.** Incidence Rates for Premature Death After Discharge From Psychiatric Hospitalization, Total and Stratified by Sex **eTable 7.** Incidence Rates for Suicide After Discharge From Psychiatric Hospitalization, Total and Stratified by Sex **eTable 8.** Incidence Rates for Intentional Self-Harm After Discharge From Psychiatric Hospitalization, Total and Stratified by Sex **eTable 9.** Lethality Index of Intentional Self-Harm After Discharge From Psychiatric Hospitalization, Total and Stratified by Sex **eTable 10.** Hazard Ratios for Premature Death After Discharge From Psychiatric Hospitalization, Total and Stratified by Sex **eTable 11.** Hazard Ratios for Suicide After Discharge From Psychiatric Hospitalization, Total and Stratified by Sex **eTable 12.** Hazard Ratios for Intentional Self-Harm After Discharge From Psychiatric Hospitalization, Total and Stratified by Sex **eTable 13.** Hazard Ratios for Premature Death After Discharge From Psychiatric Hospitalization, Total and Stratified by Postdischarge Follow-Up Time **eTable 14.** Hazard Ratios for Suicide After Discharge From Psychiatric Hospitalization, Total and Stratified by Postdischarge Follow-Up Time **eTable 15.** Hazard Ratios for Intentional Self-Harm After Discharge From Psychiatric Hospitalization, Total and Stratified by Postdischarge Follow-Up Time

Supplement 2.
Data Sharing Statement
