# Supplementary material for: Premature Death, Suicide, and Nonlethal Intentional Self-Harm After Psychiatric Discharge
Source: JAMA Netw Open. 2024 Jun 26;7(6):e2417131. doi: 10.1001/jamanetworkopen.2024.17131 (PMC11208976; doi:10.1001/jamanetworkopen.2024.17131)
Supplement: Supplement 1. — eTable 1. Mental Disorder Diagnosis Categories and Corresponding ICD-9-CM and ICD-10-CM Diagnostic Codes eTable 2. Study Cohort Descriptive Statistics eTable 3. Cumulative Incidence Estimates of Adverse Outcomes After Discharge From Psychiatric Hospitalization, Stratified by Postdischarge Follow-Up Time eTable 4. Standardized Mortality Ratios for Premature Mortality eTable 5. Standardized Mortality Ratios for Suicide eTable 6. Incidence Rates for Premature Death After Discharge From Psychiatric Hospitalization, Total and Stratified by Sex eTable 7. Incidence Rates for Suicide After Discharge From Psychiatric Hospitalization, Total and Stratified by Sex eTable 8. Incidence Rates for Intentional Self-Harm After Discharge From Psychiatric Hospitalization, Total and Stratified by Sex eTable 9. Lethality Index of Intentional Self-Harm After Discharge From Psychiatric Hospitalization, Total and Stratified by Sex eTable 10. Hazard Ratios for Premature Death After Discharge From Psychiatric Hospitalization, Total and Stratified by Sex eTable 11. Hazard Ratios for Suicide After Discharge From Psychiatric Hospitalization, Total and Stratified by Sex eTable 12. Hazard Ratios for Intentional Self-Harm After Discharge From Psychiatric Hospitalization, Total and Stratified by Sex eTable 13. Hazard Ratios for Premature Death After Discharge From Psychiatric Hospitalization, Total and Stratified by Postdischarge Follow-Up Time eTable 14. Hazard Ratios for Suicide After Discharge From Psychiatric Hospitalization, Total and Stratified by Postdischarge Follow-Up Time eTable 15. Hazard Ratios for Intentional Self-Harm After Discharge From Psychiatric Hospitalization, Total and Stratified by Postdischarge Follow-Up Time [file jamanetwopen-e2417131-s001.pdf]

Supplementary Online Content

Mortier P, Conde S, Alayo I, et al. Premature death, suicide, and nonlethal intentional self-harm after psychiatric discharge. *JAMA Netw Open*. 2024;7(6):e2417131. doi:10.1001/jamanetworkopen.2024.17131

eTable 1. Mental Disorder Diagnosis Categories and Corresponding *ICD-9-CM* and *ICD-10-CM* Diagnostic Codes

eTable 2. Study Cohort Descriptive Statistics

eTable 3. Cumulative Incidence Estimates of Adverse Outcomes After Discharge From Psychiatric Hospitalization, Stratified by Postdischarge Follow-Up Time

eTable 4. Standardized Mortality Ratios for Premature Mortality

eTable 5. Standardized Mortality Ratios for Suicide

eTable 6. Incidence Rates for Premature Death After Discharge From Psychiatric Hospitalization, Total and Stratified by Sex

eTable 7. Incidence Rates for Suicide After Discharge From Psychiatric Hospitalization, Total and Stratified by Sex

eTable 8. Incidence Rates for Intentional Self-Harm After Discharge From Psychiatric Hospitalization, Total and Stratified by Sex

eTable 9. Lethality Index of Intentional Self-Harm After Discharge From Psychiatric Hospitalization, Total and Stratified by Sex

eTable 10. Hazard Ratios for Premature Death After Discharge From Psychiatric Hospitalization, Total and Stratified by Sex

eTable 11. Hazard Ratios for Suicide After Discharge From Psychiatric Hospitalization, Total and Stratified by Sex

eTable 12. Hazard Ratios for Intentional Self-Harm After Discharge From Psychiatric Hospitalization, Total and Stratified by Sex

eTable 13. Hazard Ratios for Premature Death After Discharge From Psychiatric Hospitalization, Total and Stratified by Postdischarge Follow-Up Time

eTable 14. Hazard Ratios for Suicide After Discharge From Psychiatric Hospitalization, Total and Stratified by Postdischarge Follow-Up Time

eTable 15. Hazard Ratios for Intentional Self-Harm After Discharge From Psychiatric Hospitalization, Total and Stratified by Postdischarge Follow-Up Time

This supplementary material has been provided by the authors to give readers additional information about their work.

© 2024 Mortier P et al. *JAMA Network Open*.

eTable 1. Mental Disorder Diagnosis Categories and Corresponding *ICD-9-CM* and *ICD-10-CM* diagnostic codes

Mental Disorder Diagnosis Category	ICD-9-CM	ICD-10-CM
Adjustment disorders	309.0; 309.1; 309.22; 309.23; 309.24; 309.28; 309.29; 309.3; 309.4; 309.82; 309.83; 309.89; 309.9	F43.20; F43.21; F43.22; F43.23; F43.24; F43.25; F43.29; F43.8; F43.9
Alcohol-related disorders	291.0; 291.1; 291.2; 291.3; 291.4; 291.5; 291.81; 291.82; 291.89; 291.9; 303.00; 303.01; 303.02; 303.03; 303.90; 303.91; 303.92; 303.93; 305.00; 305.01; 305.02; 305.03	F10.10; F10.11; F10.120; F10.121; F10.129; F10.130; F10.131; F10.132; F10.139; F10.14; F10.150; F10.151; F10.159; F10.180; F10.181; F10.182; F10.188; F10.19; F10.20; F10.21; F10.220; F10.221; F10.229; F10.230; F10.231; F10.232; F10.239; F10.24; F10.250; F10.251; F10.259; F10.26; F10.27; F10.280; F10.281; F10.282; F10.288; F10.29; F10.920; F10.921; F10.929; F10.930; F10.931; F10.932; F10.939; F10.94; F10.950; F10.951; F10.959; F10.96; F10.97; F10.980; F10.981; F10.982; F10.988; F10.99
Anxiety disorders	293.84; 300.00; 300.01; 300.02; 300.09; 300.10; 300.20; 300.21; 300.22; 300.23; 300.29; 300.3; 300.5; 300.9; 308.0; 308.1; 308.2; 308.3; 308.4; 308.9; 309.81; 313.0; 313.1; 313.21; 313.22; 313.3; 313.82; 313.83	F06.4; F40.00; F40.01; F40.02; F40.10; F40.11; F40.210; F40.218; F40.220; F40.228; F40.230; F40.231; F40.232; F40.233; F40.240; F40.241; F40.242; F40.243; F40.248; F40.290; F40.291; F40.298; F40.8; F40.9; F41.0; F41.1; F41.3; F41.8; F41.9; F42.2; F42.3; F42.4; F42.8; F42.9; F43.0; F43.10; F43.11; F43.12; F45.8; F48.8; F48.9
Attention-deficit/hyperactivity disorder	314.00; 314.01; 314.1; 314.2; 314.8; 314.9	F90.0; F90.1; F90.2; F90.8; F90.9
Bipolar disorders	296.00; 296.01; 296.02; 296.03; 296.04; 296.05; 296.06; 296.10; 296.11; 296.12; 296.13; 296.14; 296.15; 296.16; 296.40; 296.41; 296.42; 296.43; 296.44; 296.45; 296.46; 296.50; 296.51; 296.52; 296.53; 296.54; 296.55; 296.56; 296.60; 296.61; 296.62; 296.63; 296.64; 296.65; 296.66; 296.7; 296.80; 296.81; 296.82; 296.89; 296.90; 296.99	F30.10; F30.11; F30.12; F30.13; F30.2; F30.3; F30.4; F30.8; F30.9; F31.0; F31.10; F31.11; F31.12; F31.13; F31.2; F31.30; F31.31; F31.32; F31.4; F31.5; F31.60; F31.61; F31.62; F31.63; F31.64; F31.70; F31.71; F31.72; F31.73; F31.74; F31.75; F31.76; F31.77; F31.78; F31.81; F31.89; F34.81; F31.9
Conduct disorder or Oppositional defiant disorder	312.00; 312.01; 312.02; 312.03; 312.10; 312.11; 312.12; 312.13; 312.20; 312.21; 312.22; 312.23; 312.4; 312.81; 312.82; 312.89; 312.9; 313.81	F91.0; F91.1; F91.2; F91.3; F91.8; F91.9
Delirium, dementia, and amnestic and other cognitive disorders	290.0; 290.10; 290.11; 290.12; 290.13; 290.20; 290.21; 290.3; 290.40; 290.41; 290.42; 290.43; 290.8; 290.9; 293.0; 293.1; 294.0; 294.10; 294.11; 294.20; 294.21; 294.8; 294.9; 310.0; 310.2; 310.81; 310.89; 310.9; 331.0; 331.11; 331.19; 331.2; 331.82	F01.50; F01.51; F02.80; F02.81; F03.90; F03.91; F04; F05; F07.0; F07.81; F07.89; F07.9; F09; F48.2; G30.0; G30.1; G30.8; G30.9; G31.01; G31.09; G31.1; G31.81; G31.83; G31.84; G31.9
Depressive disorders	293.83; 296.20; 296.21; 296.22; 296.23; 296.24; 296.25; 296.26; 296.30; 296.31; 296.32; 296.33; 296.34; 296.35; 296.36; 300.4; 311	F32.0; F32.1; F32.2; F32.3; F32.4; F32.5; F32.81; F32.89; F32.9; F33.0; F33.1; F33.2; F33.3; F33.40; F33.41; F33.42; F33.8; F33.9; F34.1; F34.89; F34.9; F39; F53.0
Developmental disorders	307.0; 307.9; 315.00; 315.01; 315.02; 315.09; 315.1; 315.2; 315.31; 315.32; 315.34; 315.35; 315.39; 315.4; 315.5; 315.8; 315.9; 317; 318.0; 318.1; 318.2; 319	F70; F71; F72; F73; F78; F79; F80.0; F80.1; F80.2; F80.4; F80.81; F80.82; F80.89; F80.9; F81.0; F81.2; F81.81; F81.89; F81.9; F82; F88; F89; F98.5
Disorders usually diagnosed in infancy, childhood, or adolescence	299.00; 299.01; 299.10; 299.11; 299.80; 299.81; 299.90; 299.91; 307.20; 307.21; 307.22; 307.23; 307.3; 307.6; 307.7; 309.21; 313.23; 313.89; 313.9	F84.0; F84.2; F84.3; F84.5; F84.8; F84.9; F93.0; F93.8; F93.9; F94.0; F94.1; F94.2; F94.8; F94.9; F95.0; F95.1; F95.2; F95.8; F95.9; F98.0; F98.1; F98.21; F98.29; F98.3; F98.4; F98.8; F98.9

© 2024 Mortier P et al. *JAMA Network Open*.

Eating disorders	307.1; 307.50; 307.51; 307.52; 307.53; 307.54; 307.59	F50.00; F50.01; F50.02; F50.2; F50.81; F50.82; F50.89; F50.9
Other disorders	293.89; 293.9; 300.11; 300.12; 300.13; 300.14; 300.15; 300.16; 300.19; 300.6; 300.7; 300.81; 300.82; 300.89; 302.0; 302.1; 302.2; 302.3; 302.4; 302.50; 302.51; 302.52; 302.53; 302.6; 302.70; 302.71; 302.72; 302.73; 302.74; 302.75; 302.76; 302.79; 302.81; 302.82; 302.83; 302.84; 302.85; 302.89; 302.9; 306.0; 306.1; 306.2; 306.3; 306.4; 306.50; 306.51; 306.52; 306.53; 306.59; 306.6; 306.7; 306.8; 306.9; 307.40; 307.41; 307.42; 307.43; 307.44; 307.45; 307.46; 307.47; 307.48; 307.49; 307.80; 307.81; 307.89; 310.1; 312.30; 312.31; 312.32; 312.33; 312.34; 312.35; 312.39; 316	F06.1; F06.30; F06.31; F06.32; F06.33; F06.34; F06.8; F44.0; F44.1; F44.2; F44.4; F44.5; F44.6; F44.7; F44.81; F44.89; F44.9; F45.0; F45.1; F45.20; F45.21; F45.22; F45.29; F45.41; F45.42; F45.9; F48.1; F51.01; F51.02; F51.03; F51.04; F51.05; F51.09; F51.11; F51.12; F51.13; F51.19; F51.3; F51.4; F51.5; F51.8; F51.9; F52.0; F52.1; F52.21; F52.22; F52.31; F52.32; F52.4; F52.5; F52.6; F52.8; F52.9; F54; F59; F63.0; F63.1; F63.2; F63.3; F63.81; F63.89; F63.9; F64.0; F64.1; F64.2; F64.8; F64.9; F65.0; F65.1; F65.2; F65.3; F65.4; F65.50; F65.51; F65.52; F65.81; F65.89; F65.9; F66; F68.10; F68.11; F68.12; F68.13; F68.8; F68A; F99
Personality disorders	301.0; 301.10; 301.11; 301.12; 301.13; 301.20; 301.21; 301.22; 301.3; 301.4; 301.50; 301.51; 301.59; 301.6; 301.7; 301.81; 301.82; 301.83; 301.84; 301.89; 301.9	F34.0; F60.0; F60.1; F60.2; F60.3; F60.4; F60.5; F60.6; F60.7; F60.81; F60.89; F60.9; F69
Schizophrenia and other psychotic disorders	293.81; 293.82; 295.00; 295.01; 295.02; 295.03; 295.04; 295.05; 295.10; 295.11; 295.12; 295.13; 295.14; 295.15; 295.20; 295.21; 295.22; 295.23; 295.24; 295.25; 295.30; 295.31; 295.32; 295.33; 295.34; 295.35; 295.40; 295.41; 295.42; 295.43; 295.44; 295.45; 295.50; 295.51; 295.52; 295.53; 295.54; 295.55; 295.60; 295.61; 295.62; 295.63; 295.64; 295.65; 295.70; 295.71; 295.72; 295.73; 295.74; 295.75; 295.80; 295.81; 295.82; 295.83; 295.84; 295.85; 295.90; 295.91; 295.92; 295.93; 295.94; 295.95; 297.0; 297.1; 297.2; 297.3; 297.8; 297.9; 298.0; 298.1; 298.2; 298.3; 298.4; 298.8; 298.9	F06.0; F06.2; F20.0; F20.1; F20.2; F20.3; F20.5; F20.81; F20.89; F20.9; F21; F22; F23; F24; F25.0; F25.1; F25.8; F25.9; F28; F29; F53; F53.1
Drug-related disorders	292.0; 292.11; 292.12; 292.2; 292.81; 292.82; 292.83; 292.84; 292.85; 292.89; 292.9; 304.00; 304.01; 304.02; 304.03; 304.10; 304.11; 304.12; 304.13; 304.20; 304.21; 304.22; 304.23; 304.30; 304.31; 304.32; 304.33; 304.40; 304.41; 304.42; 304.43; 304.50; 304.51; 304.52; 304.53; 304.60; 304.61; 304.62; 304.63; 304.70; 304.71; 304.72; 304.73; 304.80; 304.81; 304.82; 304.83; 304.90; 304.91; 304.92; 304.93; 305.20; 305.21; 305.22; 305.23; 305.30; 305.31; 305.32; 305.33; 305.40; 305.41; 305.42; 305.43; 305.50; 305.51; 305.52; 305.53; 305.60; 305.61; 305.62; 305.63; 305.70; 305.71; 305.72; 305.73; 305.80; 305.81; 305.82; 305.83; 305.90; 305.91; 305.92; 305.93	F11.10; F11.11; F11.120; F11.121; F11.122; F11.129; F11.13; F11.14; F11.150; F11.151; F11.159; F11.181; F11.182; F11.188; F11.19; F11.20; F11.21; F11.220; F11.221; F11.222; F11.229; F11.23; F11.24; F11.250; F11.251; F11.259; F11.281; F11.282; F11.288; F11.29; F11.90; F11.920; F11.921; F11.922; F11.929; F11.93; F11.94; F11.950; F11.951; F11.959; F11.981; F11.982; F11.988; F11.99; F12.10; F12.11; F12.120; F12.121; F12.122; F12.129; F12.13; F12.150; F12.151; F12.159; F12.180; F12.188; F12.19; F12.20; F12.21; F12.220; F12.221; F12.222; F12.229; F12.23; F12.250; F12.251; F12.259; F12.280; F12.288; F12.29; F12.90; F12.920; F12.921; F12.922; F12.929; F12.93; F12.950; F12.951; F12.959; F12.980; F12.988; F12.99; F13.10; F13.11; F13.120; F13.121; F13.129; F13.130; F13.131; F13.132; F13.139; F13.14; F13.150; F13.151; F13.159; F13.180; F13.181; F13.182; F13.188; F13.19; F13.20; F13.21; F13.220; F13.221; F13.229; F13.230; F13.231; F13.232; F13.239; F13.24; F13.250; F13.251; F13.259; F13.26; F13.27; F13.280; F13.281; F13.282; F13.288; F13.29; F13.90; F13.920; F13.921; F13.929; F13.930; F13.931; F13.932; F13.939; F13.94; F13.950; F13.951; F13.959; F13.96; F13.97; F13.980; F13.981; F13.982; F13.988; F13.99; F14.10; F14.11; F14.120; F14.121; F14.122; F14.129; F14.13; F14.14; F14.150; F14.151; F14.159; F14.180; F14.181; F14.182; F14.188; F14.19; F14.20; F14.21; F14.220; F14.221; F14.222; F14.229; F14.23; F14.24; F14.250; F14.251; F14.259; F14.280; F14.281;

© 2024 Mortier P et al. *JAMA Network Open*.

		F14.282; F14.288; F14.29; F14.90; F14.920; F14.921; F14.922; F14.929; F14.93; F14.94; F14.950; F14.951; F14.959; F14.980; F14.981; F14.982; F14.988; F14.99; F15.10; F15.11; F15.120; F15.121; F15.122; F15.129; F15.13; F15.14; F15.150; F15.151; F15.159; F15.180; F15.181; F15.182; F15.188; F15.19; F15.20; F15.21; F15.220; F15.221; F15.222; F15.229; F15.23; F15.24; F15.250; F15.251; F15.259; F15.280; F15.281; F15.282; F15.288; F15.29; F15.90; F15.920; F15.921; F15.922; F15.929; F15.93; F15.94; F15.950; F15.951; F15.959; F15.980; F15.981; F15.982; F15.988; F15.99; F16.10; F16.11; F16.120; F16.121; F16.122; F16.129; F16.14; F16.150; F16.151; F16.159; F16.180; F16.183; F16.188; F16.19; F16.20; F16.21; F16.220; F16.221; F16.229; F16.24; F16.250; F16.251; F16.259; F16.280; F16.283; F16.288; F16.29; F16.90; F16.920; F16.921; F16.929; F16.94; F16.950; F16.951; F16.959; F16.980; F16.983; F16.988; F16.99; F18.10; F18.11; F18.120; F18.121; F18.129; F18.14; F18.150; F18.151; F18.159; F18.17; F18.180; F18.188; F18.19; F18.20; F18.21; F18.220; F18.221; F18.229; F18.24; F18.250; F18.251; F18.259; F18.27; F18.280; F18.288; F18.29; F18.90; F18.920; F18.921; F18.929; F18.94; F18.950; F18.951; F18.959; F18.97; F18.980; F18.988; F18.99; F19.10; F19.11; F19.120; F19.121; F19.122; F19.129; F19.130; F19.131; F19.132; F19.139; F19.14; F19.150; F19.151; F19.159; F19.16; F19.17; F19.180; F19.181; F19.182; F19.188; F19.19; F19.20; F19.21; F19.220; F19.221; F19.222; F19.229; F19.230; F19.231; F19.232; F19.239; F19.24; F19.250; F19.251; F19.259; F19.26; F19.27; F19.280; F19.281; F19.282; F19.288; F19.29; F19.90; F19.920; F19.921; F19.922; F19.929; F19.930; F19.931; F19.932; F19.939; F19.94; F19.950; F19.951; F19.959; F19.96; F19.97; F19.980; F19.981; F19.982; F19.988; F19.99; F55.0; F55.1; F55.2; F55.3; F55.4; F55.8

© 2024 Mortier P et al. *JAMA Network Open*.

eTable 2. Study Cohort Descriptive Statistics

	Total Cohort (n=49108)[a]						Subcohort used for premature death analyses (n=44267)[b]					
	Total (n=49101)		Females (n=23271)		Males (n=25830)		Total (n=44272)		Females (n=20134)		Males (n=24138)	
**Independent variables**	**n**	**%**	**n**	**%**	**n**	**%**	**n**	**%**	**n**	**%**	**n**	**%**
**Sex**												
male	25833	52.6	/	/	25833	100.0	24136	54.5	/	/	24136	100.0
female	23275	47.4	23275	100.0	/	/	20131	45.5	20131	100.0	/	/
**Age at discharge from index hospitalization, y**												
10-14	2154	4.4	1259	5.4	895	3.5	2154	4.9	1259	6.3	895	3.7
15-19	3384	6.9	1877	8.1	1507	5.8	3384	7.6	1877	9.3	1507	6.2
20-29	5915	12.0	2087	9.0	3828	14.8	5915	13.4	2087	10.4	3828	15.9
30-39	9157	18.6	3649	15.7	5508	21.3	9157	20.7	3649	18.1	5508	22.8
40-49	11095	22.6	4848	20.8	6247	24.2	11095	25.1	4848	24.1	6247	25.9
50-59	8052	16.4	3899	16.8	4153	16.1	8052	18.2	3899	19.4	4153	17.2
60-69	4510	9.2	2512	10.8	1998	7.7	4510	10.2	2512	12.5	1998	8.3
70 or more	4841	9.9	3144	13.5	1697	6.6	/	/	/	/	/	/
**Socio-economic group**												
contributory annual income < 18000 €/y	34164	69.6	15924	68.4	18240	70.6	30452	68.8	13444	66.8	17008	70.5
contributory annual income > 18000 €/y	6512	13.3	3472	14.9	3040	11.8	5654	12.8	3000	14.9	2654	11.0
socio-economically vulnerable categories	8432	17.2	3879	16.7	4553	17.6	8161	18.4	3687	18.3	4474	18.5
**Diagnosis of intentional self-harm associated with index hospitalization**												
yes	1779	3.6	1094	4.7	685	2.7	1634	3.7	1008	5.0	626	2.6
**Intentional self-harm after discharge from index psychiatric hospitalization**												
yes	4752	9.7	2863	12.3	1889	7.3	4569	10.3	2745	13.6	1824	7.6
**Mental disorders associated with index hospitalization**												
Adjustment disorders	4901	10.0	2736	11.8	2165	8.4	4633	10.5	2586	12.8	2047	8.5
Alcohol-related disorders	11949	24.3	3414	14.7	8535	33.0	11602	26.2	3310	16.4	8292	34.4
Anxiety disorders	4095	8.3	2126	9.1	1969	7.6	3839	8.7	1940	9.6	1899	7.9
Attention-deficit/hyperactivity disorder	1001	2.0	306	1.3	695	2.7	998	2.3	305	1.5	693	2.9
Bipolar disorders	6366	13.0	3470	14.9	2896	11.2	5801	13.1	3090	15.3	2711	11.2
Conduct disorder or oppositional defiant disorder	1713	3.5	779	3.3	934	3.6	1587	3.6	706	3.5	881	3.7
Delirium, dementia, and amnestic and other cognitive disorders	3108	6.3	1674	7.2	1434	5.6	1230	2.8	498	2.5	732	3.0

© 2024 Mortier P et al. *JAMA Network Open*.

Depressive disorders	9277	18.9	5759	24.7	3518	13.6	7589	17.1	4561	22.7	3028	12.5
Developmental disorders	1834	3.7	753	3.2	1081	4.2	1793	4.1	731	3.6	1062	4.4
Disorders usually diagnosed in infancy, childhood, or adolescence	871	1.8	221	0.9	650	2.5	868	2.0	221	1.1	647	2.7
Eating disorders	1418	2.9	1288	5.5	130	0.5	1413	3.2	1284	6.4	129	0.5
Personality disorders	8246	16.8	4434	19.1	3812	14.8	7889	17.8	4205	20.9	3684	15.3
Schizophrenia and other psychotic disorders	15629	31.8	6252	26.9	9377	36.3	14706	33.2	5609	27.9	9097	37.7
Drug-related disorders	14089	28.7	3936	16.9	10153	39.3	13958	31.5	3850	19.1	10108	41.9
Other disorders	2120	4.3	939	4.0	1181	4.6	1929	4.4	813	4.0	1116	4.6
**Duration of index hospitalization, d**												
0-6	11497	23.4	5758	24.7	5739	22.2	10135	22.9	4900	24.3	5235	21.7
7-13	10290	21.0	4484	19.3	5806	22.5	9467	21.4	3973	19.7	5494	22.8
14-20	8892	18.1	4136	17.8	4756	18.4	8179	18.5	3687	18.3	4492	18.6
21-27	5897	12.0	2807	12.1	3090	12.0	5326	12.0	2423	12.0	2903	12.0
28 or more	12532	25.5	6090	26.2	6442	24.9	11160	25.2	5148	25.6	6012	24.9
**Psychiatric hospitalizations in the previous 6 y, No.**												
0	37151	75.7	17918	77.0	19233	74.5	32941	74.4	15207	75.5	17734	73.5
1	5985	12.2	2669	11.5	3316	12.8	5597	12.6	2410	12.0	3187	13.2
2	2489	5.1	1120	4.8	1369	5.3	2375	5.4	1037	5.2	1338	5.5
3	1304	2.7	567	2.4	737	2.9	1268	2.9	542	2.7	726	3.0
4	761	1.5	340	1.5	421	1.6	744	1.7	327	1.6	417	1.7
5 or more	1418	2.9	661	2.8	757	2.9	1342	3.0	608	3.0	734	3.0
**Covariates**												
**Health region**												
Lleida	2497	5.1	1242	5.3	1255	4.9	2244	5.1	1072	5.3	1172	4.9
Camp de Tarragona	3901	7.9	1718	7.4	2183	8.5	3657	8.3	1576	7.8	2081	8.6
Terres de l'Ebre	896	1.8	407	1.7	489	1.9	813	1.8	357	1.8	456	1.9
Girona	4719	9.6	2190	9.4	2529	9.8	4291	9.7	1894	9.4	2397	9.9
Central Catalonia	3484	7.1	1620	7.0	1864	7.2	3193	7.2	1429	7.1	1764	7.3
High Pyrenees and Aran	342	0.7	176	0.8	166	0.6	317	0.7	159	0.8	158	0.7
Barcelona Metropolitan South	9543	19.4	4713	20.2	4830	18.7	8457	19.1	4032	20.0	4425	18.3
Barcelona Metropolitan North	11722	23.9	5504	23.6	6218	24.1	10714	24.2	4862	24.2	5852	24.2
Barcelona City	12004	24.4	5705	24.5	6299	24.4	10581	23.9	4750	23.6	5831	24.2
**Year of discharge**												
2014	13302	27.1	6249	26.8	7053	27.3	12222	27.6	5537	27.5	6685	27.7
2015	10333	21.0	4844	20.8	5489	21.2	9359	21.1	4203	20.9	5156	21.4

© 2024 Mortier P et al. *JAMA Network Open*.

2016	9465	19.3	4503	19.3	4962	19.2	8541	19.3	3885	19.3	4656	19.3
2017	8350	17.0	4019	17.3	4331	16.8	7418	16.8	3416	17.0	4002	16.6
2018	7658	15.6	3660	15.7	3998	15.5	6727	15.2	3090	15.3	3637	15.1

a The cohort consists of all patients with a psychiatric hospitalization between January 1, 2014, and December 31, 2018, in the region of Catalonia, Spain (7.6 million population). The first hospitalization within this timeframe was designated as the index hospitalization. We excluded patients aged <10 at discharge from index hospitalization (n=297), with missing values for health region (n=8), and patients who died during index hospitalization (n=114).

b subcohort used for premature death analysis, i.e., the subcohort aged 10-69 years old at discharge.

© 2024 Mortier P et al. *JAMA Network Open*.

eTable 3. Cumulative Incidence Estimates of Adverse Outcomes After Discharge From Psychiatric Hospitalization, Stratified by Post-Discharge Follow-Up Time

	0 days	7 days		30 days		90 days	
**Post-discharge adverse outcomes**	**n at risk**	**n at risk**	**CumI, % (95% CI)**	**n at risk**	**CumI, % (95% CI)**	**n at risk**	**CumI, % (95% CI)**
**Premature death**							
Female	20131	20107	0.07 (0.04 - 0.12)	20063	0.19 (0.14 - 0.26)	19953	0.45 (0.36 - 0.55)
Male	24136	24119	0.05 (0.02 - 0.08)	24032	0.34 (0.27 - 0.42)	23879	0.78 (0.67 - 0.89)
Total	44267	44226	0.06 (0.04 - 0.09)	44095	0.27 (0.23 - 0.32)	43832	0.63 (0.56 - 0.70)
**Death by suicide**							
Female	23275	23242	0.02 (0.01 - 0.05)	23177	0.08 (0.05 - 0.12)	23055	0.13 (0.09 - 0.19)
Male	25833	25807	0.02 (0.01 - 0.05)	25696	0.12 (0.08 - 0.17)	25515	0.21 (0.16 - 0.27)
Total	49108	49049	0.02 (0.01 - 0.04)	48873	0.10 (0.08 - 0.13)	48570	0.17 (0.14 - 0.21)
**Non-lethal intentional self-harm**							
Female	23275	23065	0.85 (0.74 - 0.97)	22736	1.93 (1.75 - 2.11)	22287	3.35 (3.13 - 3.59)
Male	25833	25682	0.54 (0.45 - 0.63)	25414	1.12 (1.00 - 1.25)	25062	1.82 (1.66 - 1.98)
Total	49108	48747	0.68 (0.61 - 0.76)	48150	1.50 (1.40 - 1.61)	47349	2.54 (2.41 - 2.69)

Abbreviations: CumI = cumulative incidence; CI = confidence interval.

© 2024 Mortier P et al. *JAMA Network Open*.

eTable 3. ***(continued)***

	0 days	365 days (1 year)		730 days (2 years)		1095 days (3 years)	
**Post-discharge adverse outcomes**	**n at risk**	**n at risk**	**CumI, % (95% CI)**	**n at risk**	**CumI, % (95% CI)**	**n at risk**	**CumI, % (95% CI)**
**Premature death**							
Female	20131	19600	1.21 (1.07 - 1.37)	16042	2.25 (2.05 - 2.47)	12381	3.16 (2.91 - 3.42)
Male	24136	23344	2.20 (2.02 - 2.39)	19172	3.74 (3.50 - 3.99)	14878	5.34 (5.04 - 5.64)
Total	44267	42944	1.75 (1.63 - 1.88)	35214	3.07 (2.90 - 3.23)	27259	4.35 (4.15 - 4.56)
**Death by suicide**							
Female	23275	22653	0.30 (0.24 - 0.38)	18625	0.44 (0.36 - 0.53)	14450	0.61 (0.51 - 0.72)
Male	25833	24901	0.50 (0.42 - 0.59)	20388	0.74 (0.64 - 0.85)	15828	0.94 (0.82 - 1.07)
Total	49108	47554	0.40 (0.35 - 0.46)	39013	0.59 (0.53 - 0.67)	30278	0.78 (0.71 - 0.87)
**Non-lethal intentional self-harm**							
Female	23275	21057	6.99 (6.66 - 7.32)	16842	9.63 (9.25 - 10.02)	12827	11.40 (10.98 - 11.83)
Male	25833	24010	3.62 (3.40 - 3.86)	19313	5.29 (5.02 - 5.58)	14802	6.64 (6.33 - 6.96)
Total	49108	45067	5.22 (5.02 - 5.42)	36155	7.35 (7.12 - 7.58)	27629	8.90 (8.64 - 9.16)

Abbreviations: CumI = cumulative incidence; CI = confidence interval.

© 2024 Mortier P et al. *JAMA Network Open*.

eTable 3. ***(continued)***

	0 days	1460 days (4 years)		1825 days (5 years)		2190 days (6 years)	
**Post-discharge adverse outcomes**	**n at risk**	**n at risk**	**CumI, % (95% CI)**	**n at risk**	**CumI, % (95% CI)**	**n at risk**	**CumI, % (95% CI)**
**Premature death**							
Female	20131	8624	4.05 (3.75 - 4.37)	4851	4.77 (4.41 - 5.14)	3	6.36 (5.22 - 7.64)
Male	24136	10360	6.62 (6.27 - 6.98)	5707	8.15 (7.72 - 8.59)	2	9.56 (8.98 - 10.17)
Total	44267	18984	5.46 (5.22 - 5.70)	10558	6.62 (6.34 - 6.92)	5	8.11 (7.51 - 8.74)
**Death by suicide**							
Female	23275	10080	0.76 (0.64 - 0.89)	5600	0.87 (0.73 - 1.02)	3	0.98 (0.81 - 1.19)
Male	25833	11050	1.15 (1.02 - 1.31)	6049	1.28 (1.12 - 1.44)	2	1.43 (1.23 - 1.65)
Total	49108	21130	0.97 (0.88 - 1.07)	11649	1.08 (0.98 - 1.20)	5	1.22 (1.08 - 1.37)
**Non-lethal intentional self-harm**							
Female	23275	8774	12.84 (12.38 - 13.30)	4731	14.22 (13.70 - 14.74)	3	15.43 (14.79 - 16.08)
Male	25833	10238	7.56 (7.21 - 7.91)	5553	8.68 (8.28 - 9.09)	2	9.73 (9.23 - 10.24)
Total	49108	19012	10.06 (9.77 - 10.35)	10284	11.30 (10.98 - 11.63)	5	12.42 (12.02 - 12.83)

Abbreviations: CumI = cumulative incidence; CI = confidence interval.

© 2024 Mortier P et al. *JAMA Network Open*.

eTable 4. Standardized Mortality Ratios for Premature Mortality

	Entire follow-up time (median = 1327 days)[a]			One-year follow-up time[b]		
	Males	Females	Total	Males	Females	Total
Age, y	SMR (95% CI)	SMR (95% CI)	SMR (95% CI)	SMR (95% CI)	SMR (95% CI)	SMR (95% CI)
10-14	13.2 (0.3-26.1)	29.1 (11.1-47.1)	21.6 (10.3-32.9)	12.0 (-11.5-35.4)	42.3 (0.8-83.7)	28.1 (3.5-52.6)
15-19	19.7 (11.8-27.5)	8.4 (2.2-14.6)	15.1 (9.8-20.4)	32.7 (13.4-52.1)	8.7 (-3.4-20.7)	22.9 (10.5-35.4)
20-24	14.1 (9.1-19.1)	23.7 (10.8-36.6)	16 (11.3-20.7)	21.5 (9.8-33.1)	39.8 (7.9-71.6)	25.1 (13.8-36.4)
25-29	18.3 (13.4-23.1)	21.4 (10.6-32.2)	18.9 (14.4-23.3)	24.1 (13.6-34.7)	15.6 (-2.0-33.2)	22.5 (13.3-31.7)
30-34	22.4 (18.0-26.8)	22.7 (15.0-30.5)	22.5 (18.6-26.3)	28.2 (18.7-37.7)	37.5 (18.5-56.5)	30.5 (22.0-39.1)
35-39	18.4 (15.3-21.6)	24.4 (18.5-30.3)	20 (17.2-22.8)	24.5 (17.6-31.4)	32.7 (19.6-45.8)	26.7 (20.6-32.9)
40-44	14.9 (12.7-17.0)	16.1 (12.7-19.5)	15.2 (13.4-17.1)	15.0 (10.9-19.2)	18.4 (11.4-25.3)	16.0 (12.5-19.6)
45-49	11.8 (10.3-13.3)	10.0 (8.0-12)	11.2 (10.0-12.4)	14.5 (11.3-17.6)	8.6 (5.1-12.1)	12.6 (10.2-15.0)
50-54	7.1 (6.2-8.1)	8.1 (6.6-9.5)	7.4 (6.6-8.2)	8.3 (6.4-10.2)	7.4 (4.8-10.1)	8.0 (6.5-9.6)
55-59	5.5 (4.7-6.2)	6.2 (5.0-7.3)	5.7 (5.1-6.3)	5.9 (4.4-7.3)	6.3 (4.1-8.6)	6.0 (4.8-7.2)
60-64	5.2 (4.5-6.0)	6.9 (5.7-8.1)	5.8 (5.1-6.4)	6.4 (4.9-7.9)	7.9 (5.5-10.4)	6.9 (5.6-8.2)
65-69	2.6 (2.1-3.0)	2.8 (2.1-3.5)	2.6 (2.3-3.0)	5.6 (4.3-6.9)	5.8 (4.0-7.6)	5.7 (4.6-6.7)
**Total**	7.5 (7.1-7.8)	7.7 (7.2-8.3)	7.5 (7.2-7.9)	9.4 (8.6-10.3)	9.2 (8.0-10.3)	9.4 (8.7-10.0)

Abbreviations: CI = confidence interval; SMR = standardized mortality ratio.

a Expected age-sex stratified premature mortality rates were calculated by multiplying the average age-sex stratified annual mortality rate (period 2014-2019) by the median follow-up time in years (i.e., 3.63 years).

b Expected age-sex stratified premature mortality rates used were the average age-sex stratified annual mortality rates for the period 2014-2019.

© 2024 Mortier P et al. *JAMA Network Open*.

eTable 5. Standardized Mortality Ratios for Suicide

	Entire follow-up time (median = 1,327 days)[a]			One-year follow-up time[b]		
	Males	Females	Total	Males	Females	Total
**Age, y**	**SMR (95% CI)**	**SMR (95% CI)**	**SMR (95% CI)**	**SMR (95% CI)**	**SMR (95% CI)**	**SMR (95% CI)**
10-14	1476.1 (29.5-2922.6)	928.1 (114.6-1741.7)	1111.5 (385.3-1837.7)	1340.7 (-1287.1-3968.4)	1348.8 (-520.5-3218.2)	1346.1 (-177.2-2869.4)
15-29	41.1 (29.0-53.3)	51.4 (27.0-75.9)	43.6 (32.6-54.5)	74.7 (43.5-105.9)	87.9 (27.0-148.8)	77.8 (50.0-105.7)
30-39	30.3 (22.2-38.3)	92.6 (61.9-123.2)	41.2 (32.6-49.7)	55.0 (34.2-75.7)	192.2 (107.9-276.4)	79.0 (56.4-101.5)
40-44	28.1 (19.2-37.1)	70.7 (42.4-98.9)	36.7 (27.6-45.8)	29.6 (12.1-47.1)	160.4 (79.2-241.6)	55.9 (34.4-77.4)
45-49	33.2 (23.4-43.0)	26.1 (11.3-40.8)	31.4 (23.2-39.6)	57.6 (33.0-82.2)	15.8 (-6.1-37.7)	46.8 (27.7-65.9)
50-54	19.0 (11.5-26.4)	54.4 (31.1-77.6)	27.0 (19.2-34.8)	24.8 (8.6-41.0)	47.0 (5.8-88.3)	29.8 (14.2-45.5)
55-59	31.3 (19.5-43.1)	42.8 (22.4-63.1)	34.9 (24.6-45.2)	54.8 (25.0-84.6)	36.5 (0.7-72.4)	49.0 (25.7-72.3)
60-64	22.3 (9.7-35.0)	52.6 (24.0-81.2)	31.9 (19.4-44.3)	47.3 (12.3-82.4)	117.6 (36.1-199.1)	69.5 (34.3-104.6)
65-69	37.0 (16.9-57.2)	30.7 (10.7-50.8)	34.2 (19.9-48.5)	82.8 (25.4-140.2)	37.2 (-4.9-79.3)	62.1 (25.4-98.8)
70-74	13.0 (1.6-24.3)	28.3 (3.5-53.1)	17.8 (6.8-28.8)	37.7 (0.8-74.6)	41.1 (-15.9-98.2)	38.8 (7.7-69.8)
75-79	13.9 (1.7-26.1)	23.6 (0.5-46.7)	17.0 (5.9-28.1)	20.2 (-7.8-48.1)	21.4 (-20.6-63.5)	20.6 (-2.7-43.9)
80-84	8.4 (-1.1-17.9)	0.0 (0.0-0.0)	6.1 (-0.8-13.1)	20.4 (-7.9-48.6)	0.0 (0.0-0.0)	14.9 (-5.7-35.5)
85-89	7.5 (-7.2-22.1)	0.0 (0.0-0.0)	4.9 (-4.7-14.4)	27.2 (-26.1-80.4)	0.0 (0.0-0.0)	17.7 (-17.0-52.3)
90-94	0.0 (0.0-0.0)	0.0 (0.0-0.0)	0.0 (0.0-0.0)	0.0 (0.0-0.0)	0.0 (0.0-0.0)	0.0 (0.0-0.0)
95+	0.0 (0.0-0.0)	0.0 (0.0-0.0)	0.0 (0.0-0.0)	0.0 (0.0-0.0)	0.0 (0.0-0.0)	0.0 (0.0-0.0)
**Total**	27.9 (24.6-31.2)	47.6 (40.2-54.9)	32.9 (29.9-36.0)	47.2 (39.0-55.4)	74.7 (57.2-92.2)	54.2 (46.7-61.8)

Abbreviations: CI = confidence interval; SMR = standardized mortality ratio.

a Expected age-sex stratified suicide rates were calculated by multiplying the mean age-sex stratified annual suicide rate (period 2014-2019) by the median follow-up time in years (i.e., 3.63 years).

b Expected age-sex stratified suicide rates used were the mean age-sex stratified annual suicide rates for the period 2014-2019.

© 2024 Mortier P et al. *JAMA Network Open*.

eTable 6. Incidence Rates for Premature Death After Discharge From Psychiatric Hospitalization, Total and Stratified by Sex (n=44267)[a]

	Total	Females	Males
	IR[b] (95% CI)	IR[b] (95% CI)	IR[b] (95% CI)
**Total**	1435 (1376-1495)	1040 (965-1114)	1766 (1677-1855)
**Independent variables**			
**Age at discharge from index hospitalization, y**			
10-14	175 (83-266)	212 (81-344)	121 (33-310)
15-19	252 (163-341)	103 (41-212)	438 (263-613)
20-29	526 (430-623)	370 (233-507)	610 (481-739)
30-39	953 (850-1057)	718 (576-860)	1110 (966-1253)
40-49	1497 (1378-1616)	1006 (860-1152)	1895 (1715-2074)
50-59	2269 (2095-2443)	1589 (1381-1797)	2922 (2645-3198)
60-69	4365 (3987-4744)	2940 (2528-3353)	6219 (5535-6904)
**Socio-economic group**			
contributory annual income < 18000 €/y	1420 (1349-1491)	986 (897-1075)	1764 (1658-1871)
contributory annual income > 18000 €/y	1367 (1201-1534)	955 (765-1145)	1840 (1558-2123)
socio-economically vulnerable categories	1532 (1394-1671)	1288 (1098-1477)	1732 (1534-1931)
**Diagnosis of intentional self-harm associated with index hospitalization**	1168 (884-1452)	776 (483-1068)	1822 (1243-2401)
**Intentional self-harm after discharge from index psychiatric hospitalization**	1396 (1222-1571)	925 (742-1108)	2107 (1767-2446)
**Mental disorders associated with index hospitalization**			
Adjustment disorders	1201 (1033-1369)	740 (564-915)	1795 (1484-2106)
Alcohol-related disorders	2112 (1971-2252)	1594 (1366-1822)	2320 (2146-2495)
Anxiety disorders	1144 (962-1326)	789 (575-1003)	1493 (1200-1785)
Attention-deficit/hyperactivity disorder	516 (284-748)	444 (144-1037)	547 (261-834)
Bipolar disorders	1314 (1160-1468)	1086 (895-1277)	1577 (1330-1825)
Conduct disorder or oppositional defiant disorder	601 (396-807)	564 (269-860)	632 (348-916)
Delirium, dementia, and amnestic and other cognitive disorders	7044 (6147-7941)	5417 (4183-6652)	8156 (6904-9408)
Depressive disorders	1725 (1565-1885)	1143 (976-1310)	2622 (2308-2936)
Developmental disorders	877 (653-1100)	759 (434-1084)	959 (654-1264)
Disorders usually diagnosed in infancy, childhood, or adolescence	261 (112-513)	131 (3-732)	303 (122-625)
Eating disorders	537 (338-736)	482 (285-678)	1145 (371-2672)
Personality disorders	1299 (1168-1430)	944 (792-1097)	1711 (1489-1933)
Schizophrenia and other psychotic disorders	1209 (1116-1301)	1021 (882-1160)	1321 (1199-1444)
Drug-related disorders	1332 (1231-1433)	1131 (954-1307)	1409 (1287-1531)

© 2024 Mortier P et al. *JAMA Network Open*.

Other disorders	1379 (1096-1663)	1020 (642-1398)	1635 (1232-2039)
**Duration of hospitalization**			
0-6	1600 (1464-1737)	1036 (880-1192)	2148 (1927-2370)
7-13	1401 (1276-1527)	857 (706-1008)	1802 (1614-1989)
14-20	1353 (1220-1486)	1011 (840-1183)	1634 (1437-1831)
21-27	1296 (1136-1456)	1109 (888-1330)	1448 (1220-1676)
28 or more	1455 (1339-1572)	1170 (1016-1324)	1695 (1525-1865)
**Psychiatric hospitalizations in the previous 6 y, No.**			
0	1350 (1281-1419)	948 (863-1033)	1699 (1593-1805)
1	1548 (1386-1710)	1137 (925-1348)	1860 (1625-2095)
2	1705 (1454-1957)	1620 (1246-1994)	1769 (1431-2108)
3	1637 (1306-1968)	927 (548-1306)	2178 (1672-2685)
4	1494 (1091-1896)	757 (329-1185)	2089 (1449-2728)
>=5	1819 (1494-2145)	1653 (1191-2116)	1954 (1500-2409)
**Covariates**			
**Health region**			
Lleida	1230 (984-1476)	899 (592-1206)	1523 (1147-1899)
Camp de Tarragona	1465 (1259-1670)	1136 (860-1412)	1712 (1418-2007)
Terres de l'Ebre	1323 (913-1733)	1303 (684-1922)	1339 (792-1886)
Girona	1518 (1322-1714)	1075 (826-1323)	1869 (1578-2161)
Central Catalonia	1345 (1133-1556)	942 (678-1206)	1675 (1356-1994)
High Pyrenees and Aran	1117 (485-1749)	759 (207-1942)	1462 (631-2880)
Barcelona Metropolitan South	1548 (1407-1689)	1124 (950-1298)	1936 (1718-2154)
Barcelona Metropolitan North	1279 (1165-1392)	842 (706-977)	1647 (1473-1822)
Barcelona City	1552 (1425-1678)	1179 (1014-1344)	1854 (1668-2040)
**Year of discharge**			
2014	1465 (1370-1560)	1032 (915-1150)	1829 (1685-1973)
2015	1445 (1326-1564)	1012 (864-1161)	1799 (1620-1978)
2016	1559 (1413-1706)	1186 (996-1376)	1871 (1653-2088)
2017	1325 (1156-1494)	1064 (841-1288)	1545 (1297-1793)
2018	1063 (861-1265)	743 (493-993)	1334 (1026-1642)

Abbreviations: CI = confidence interval; IR = incidence rate.

© 2024 Mortier P et al. *JAMA Network Open*.

a All premature death analyses were restricted to the subcohort of 44267 patients with age at discharge 69 years or less, with follow-up time censored at age 70 years. Median (IQR) age at discharge among those with post-discharge premature death was 51.5 (42.1-59.0); median (IQR) age at event was 52.4 (44.0-61.4) years; median (IQR) survival time (i.e., between discharge and event) was 600 (242-1075) days.

b per 100 000 person-years

© 2024 Mortier P et al. *JAMA Network Open*.

eTable 7. Incidence Rates for Suicide After Discharge From Psychiatric Hospitalization, Total and Stratified by Sex (n=49108)[a]

	Total	Females	Males
	IR[b] (95% CI)	IR[b] (95% CI)	IR[b] (95% CI)
**Total**	250 (227-274)	195 (165-225)	301 (265-337)
**Independent variables**			
**Age at discharge from index hospitalization, y**			
10-14	112 (51-213)	106 (34-248)	121 (33-310)
15-19	114 (54-173)	44 (9-129)	201 (82-319)
20-29	217 (155-279)	185 (88-282)	234 (154-314)
30-39	260 (206-313)	256 (171-341)	262 (192-331)
40-49	290 (238-342)	198 (133-263)	365 (286-444)
50-59	313 (248-377)	270 (184-355)	354 (258-450)
60-69	312 (223-401)	252 (147-358)	394 (240-549)
70 or more	167 (99-236)	96 (44-182)	322 (153-491)
**Socio-economic group**			
contributory annual income < 18000 €/y	249 (221-277)	186 (150-221)	305 (262-347)
contributory annual income > 18000 €/y	311 (238-384)	208 (126-289)	433 (305-561)
socio-economically vulnerable categories	214 (163-264)	219 (143-294)	209 (141-278)
**Diagnosis of intentional self-harm associated with index hospitalization**	510 (331-690)	445 (234-657)	620 (295-945)
**Intentional self-harm after discharge from index psychiatric hospitalization**	646 (530-762)	458 (332-583)	934 (712-1,156)
**Mental disorders associated with index hospitalization**			
Adjustment disorders	377 (285-468)	246 (148-345)	546 (379-713)
Alcohol-related disorders	298 (246-350)	253 (164-342)	316 (253-380)
Anxiety disorders	318 (225-410)	262 (144-380)	375 (231-520)
Attention-deficit/hyperactivity disorder	217 (93-427)	266 (55-778)	195 (63-455)
Bipolar disorders	295 (226-364)	306 (211-400)	281 (180-382)
Conduct disorder or oppositional defiant disorder	103 (38-224)	112 (23-326)	96 (20-279)
Delirium, dementia and amnestic and other cognitive disorders	130 (53-207)	84 (23-216)	188 (75-387)
Depressive disorders	334 (271-397)	196 (135-257)	571 (435-706)
Developmental disorders	87 (32-190)	35 (1-195)	124 (40-290)
Disorders usually diagnosed in infancy, childhood, or adolescence	97 (20-285)	131 (3-732)	86 (10-312)
Eating disorders	210 (86-334)	167 (72-329)	671 (138-1,960)
Personality disorders	304 (242-366)	271 (192-350)	344 (247-442)
Schizophrenia and other psychotic disorders	223 (184-261)	162 (110-214)	262 (209-316)

© 2024 Mortier P et al. *JAMA Network Open*.

Drug-related disorders	250 (206-293)	244 (163-325)	252 (201-303)
Other disorders	152 (62-241)	156 (51-364)	148 (54-323)
**Duration of hospitalization**			
0-6	259 (207-311)	185 (124-246)	337 (252-422)
7-13	276 (223-330)	178 (113-243)	354 (273-435)
14-20	218 (167-269)	160 (96-224)	269 (191-347)
21-27	286 (215-357)	292 (187-396)	281 (183-378)
28 or more	228 (185-272)	194 (137-251)	261 (196-325)
**Psychiatric hospitalizations in the previous 6 y, No.**			
0	229 (202-256)	167 (135-200)	287 (245-329)
1	257 (193-320)	218 (131-305)	289 (198-379)
2	337 (228-446)	369 (199-540)	311 (171-451)
3	353 (202-504)	267 (107-550)	420 (200-641)
4	410 (203-618)	121 (15-436)	651 (297-1,004)
>=5	298 (170-425)	335 (137-533)	266 (101-430)
**Covariates**			
**Health region**			
Lleida	276 (165-386)	162 (65-334)	387 (203-572)
Camp de Tarragona	148 (85-211)	144 (66-273)	151 (66-236)
Terres de l'Ebre	298 (113-483)	263 (72-672)	328 (120-713)
Girona	328 (241-415)	255 (143-367)	392 (262-522)
Central Catalonia	270 (179-360)	270 (138-402)	269 (145-394)
High Pyrenees and Aran	/ [c]	/ [c]	/ [c]
Barcelona Metropolitan South	258 (204-312)	191 (125-257)	324 (238-410)
Barcelona Metropolitan North	224 (179-269)	140 (88-192)	300 (228-372)
Barcelona City	267 (217-316)	231 (164-297)	299 (227-372)
**Year of discharge**			
2014	259 (221-298)	204 (154-253)	310 (252-367)
2015	223 (179-267)	175 (118-232)	266 (199-332)
2016	262 (205-319)	213 (139-287)	307 (222-393)
2017	247 (179-316)	174 (91-257)	316 (208-424)
2018	275 (178-372)	205 (84-326)	340 (191-489)

Abbreviations: CI = Confidence interval; IR = incidence rate.

© 2024 Mortier P et al. *JAMA Network Open*.

a Median (IQR) age at discharge among those with post-discharge death by suicide was 44.7 (35.8-55.7) years; median (IQR) age at event was 46.8 (36.9-56.9) years; median (IQR) survival time (i.e., between discharge and event) was 430 (132-976) days.

b per 100 000 person-years

c incidence rate could not be estimated due to zero cases of suicide

© 2024 Mortier P et al. *JAMA Network Open*.

eTable 8. Incidence Rates for Non-Lethal Intentional Self-Harm After Discharge From Psychiatric Hospitalization, Total and Stratified by Sex (n=49108)[a]

	Total	Females	Males
	IR[b] (95% CI)	IR[b] (95% CI)	IR[b] (95% CI)
**Total**	2925 (2842-3009)	3788 (3649-3927)	2175 (2077-2273)
**Independent variables**			
**Age at discharge from index hospitalization, y**			
10-14	4689 (4185-5193)	6912 (6091-7734)	1926 (1443-2410)
15-19	4315 (3927-4703)	6187 (5548-6825)	2213 (1809-2618)
20-29	2737 (2509-2964)	4392 (3895-4889)	1898 (1666-2131)
30-39	2945 (2756-3133)	4022 (3668-4375)	2264 (2053-2475)
40-49	3314 (3130-3498)	4298 (3979-4617)	2560 (2344-2775)
50-59	2645 (2451-2840)	3129 (2825-3433)	2192 (1946-2438)
60-69	2160 (1919-2401)	2536 (2191-2880)	1655 (1333-1978)
70 or more	1373 (1174-1572)	1295 (1061-1528)	1542 (1167-1917)
**Socio-economic group**			
contributory annual income < 18000 €/y	2866 (2767-2965)	3701 (3535-3868)	2161 (2044-2277)
contributory annual income > 18000 €/y	2916 (2683-3149)	3643 (3286-3999)	2089 (1801-2377)
socio-economically vulnerable categories	3157 (2955-3360)	4248 (3896-4600)	2279 (2048-2510)
**Diagnosis of intentional self-harm associated with index hospitalization**	10520 (9590-11449)	12111 (10831-13391)	8059 (6760-9357)
**Mental disorders associated with index hospitalization**			
Adjustment disorders	6392 (5983-6801)	7654 (7049-8258)	4860 (4329-5391)
Alcohol-related disorders	2992 (2822-3162)	4307 (3920-4693)	2483 (2301-2666)
Anxiety disorders	4518 (4149-4886)	5728 (5138-6318)	3332 (2887-3778)
Attention-deficit/hyperactivity disorder	3212 (2612-3813)	5117 (3699-6536)	2452 (1831-3072)
Bipolar disorders	1873 (1695-2051)	2069 (1816-2322)	1636 (1388-1883)
Conduct disorder or oppositional defiant disorder	2978 (2520-3437)	4361 (3531-5192)	1861 (1374-2349)
Delirium, dementia, and amnestic and other cognitive disorders	1176 (942-1410)	1105 (801-1408)	1267 (901-1634)
Depressive disorders	4701 (4451-4951)	5358 (5018-5697)	3632 (3275-3988)
Developmental disorders	2038 (1692-2384)	2687 (2062-3312)	1596 (1199-1994)
Disorders usually diagnosed in infancy, childhood, or adolescence	2117 (1590-2643)	4028 (2536-5520)	1522 (1010-2033)
Eating disorders	5218 (4553-5884)	5566 (4844-6289)	1876 (809-3695)
Personality disorders	5271 (4996-5545)	6823 (6391-7256)	3567 (3240-3894)
Schizophrenia and other psychotic disorders	1354 (1258-1450)	1481 (1320-1642)	1272 (1152-1392)
Drug-related disorders	2796 (2646-2946)	4097 (3749-4446)	2308 (2148-2468)

© 2024 Mortier P et al. *JAMA Network Open*.

Other disorders	3417 (2974-3859)	4005 (3276-4734)	2969 (2421-3516)
**Duration of hospitalization**			
0-6	4579 (4349-4810)	5868 (5497-6238)	3317 (3041-3592)
7-13	3076 (2890-3262)	3869 (3551-4187)	2476 (2255-2697)
14-20	2617 (2435-2800)	3404 (3095-3712)	1955 (1740-2169)
21-27	2196 (1994-2399)	2817 (2482-3153)	1654 (1413-1894)
28 or more	2121 (1986-2256)	2804 (2578-3030)	1504 (1347-1661)
**Psychiatric hospitalizations in the previous 6 y, No.**			
0	2992 (2892-3092)	3858 (3693-4023)	2203 (2084-2322)
1	2278 (2083-2473)	2970 (2635-3305)	1733 (1506-1960)
2	2763 (2440-3085)	3801 (3225-4378)	1978 (1616-2339)
3	2710 (2275-3145)	3371 (2632-4109)	2209 (1687-2730)
4	3376 (2751-4001)	4224 (3172-5275)	2703 (1954-3453)
>=5	4330 (3812-4849)	5528 (4652-6404)	3362 (2747-3976)
**Covariates**			
**Health region**			
Lleida	3692 (3269-4114)	4802 (4108-5495)	2656 (2157-3154)
Camp de Tarragona	3247 (2939-3555)	4845 (4266-5424)	2075 (1750-2399)
Terres de l'Ebre	3255 (2617-3893)	3957 (2902-5012)	2695 (1916-3473)
Girona	2545 (2296-2794)	3302 (2882-3721)	1907 (1614-2200)
Central Catalonia	2730 (2432-3028)	3471 (2976-3966)	2098 (1743-2454)
High Pyrenees and Aran	3841 (2666-5017)	5013 (3086-6940)	2734 (1350-4117)
Barcelona Metropolitan South	3382 (3177-3586)	4439 (4102-4777)	2394 (2155-2634)
Barcelona Metropolitan North	2798 (2633-2964)	3524 (3252-3797)	2165 (1965-2364)
Barcelona City	2592 (2433-2751)	3227 (2968-3487)	2035 (1843-2228)
**Year of discharge**			
2014	2531 (2406-2655)	3337 (3126-3547)	1843 (1699-1988)
2015	2538 (2383-2693)	3086 (2835-3336)	2064 (1873-2255)
2016	2939 (2742-3135)	3703 (3383-4024)	2257 (2020-2493)
2017	3931 (3648-4213)	5114 (4647-5582)	2866 (2534-3198)
2018	4892 (4474-5311)	6882 (6157-7607)	3138 (2678-3598)

Abbreviations: CI = Confidence interval; IR = incidence rate.

© 2024 Mortier P et al. *JAMA Network Open*.

a Median (IQR) age at discharge among those with post-discharge non-lethal intentional self-harm was 41.1 (27.1-50.3) years; median (IQR) age at event was 42.1 (28.5-51.6) years; median (IQR) survival time (i.e., between discharge and event) was 314 (IQR=80-752) days.

b per 100 000 person-years

© 2024 Mortier P et al. *JAMA Network Open*.

eTable 9. Lethality Index[a] of Intentional Self-Harm After Discharge From Psychiatric Hospitalization, Total and Stratified by Sex (n = 49108)

	Total	Females	Males
	**Index (95% CI)**	**Index (95% CI)**	**Index (95% CI)**
**Overall**	11.7 (10.5-12.8)	19.4 (16.4-22.5)	7.2 (6.3-8.1)
**Independent variables**			
**Age at discharge from index hospitalization, y**			
10-14	41.7 (4.0-79.5)	65.1 (-22.1-152.3)	15.9 (-9.2-41.0)
15-19	37.9 (17.8-58.0)	140.5 (-130.1-411.2)	11.0 (4.2-17.9)
20-29	12.6 (8.9-16.4)	23.7 (11.0-36.4)	8.1 (5.2-11.1)
30-39	11.3 (8.9-13.8)	15.7 (10.3-21.1)	8.7 (6.2-11.1)
40-49	11.4 (9.3-13.6)	21.7 (14.4-29.0)	7.0 (5.4-8.6)
50-59	8.5 (6.6-10.3)	11.6 (7.7-15.5)	6.2 (4.4-8.0)
60-69	6.9 (4.8-9.0)	10.1 (5.6-14.5)	4.2 (2.4-6.0)
70 or more	8.2 (4.6-11.8)	13.5 (1.1-25.9)	4.8 (2.0-7.5)
**Socio-economic group**			
contributory annual income < 18000 €/y	11.5 (10.2-12.9)	19.9 (16.0-23.8)	7.1 (6.0-8.2)
contributory annual income > 18000 €/y	9.4 (7.0-11.7)	17.5 (10.4-24.6)	4.8 (3.3-6.4)
socio-economically vulnerable categories	14.8 (11.1-18.4)	19.4 (12.5-26.4)	10.9 (7.2-14.6)
**Diagnosis of intentional self-harm associated with index hospitalization**	20.6 (13.1-28.1)	27.2 (13.9-40.4)	13.0 (5.9-20.1)
**Mental disorders associated with index hospitalization**			
Adjustment disorders	17.0 (12.7-21.2)	31.1 (18.4-43.8)	8.9 (6.0-11.8)
Alcohol-related disorders	10.0 (8.2-11.9)	17.0 (10.8-23.2)	7.9 (6.2-9.5)
Anxiety disorders	14.2 (9.9-18.5)	21.8 (11.8-31.9)	8.9 (5.3-12.5)
Attention-deficit/hyperactivity disorder	14.8 (0.2-29.5)	19.2 (-18.1-56.6)	12.6 (-4.5-29.7)
Bipolar disorders	6.4 (4.8-8.0)	6.8 (4.5-9.0)	5.8 (3.6-8.1)
Conduct disorder or oppositional defiant disorder	28.9 (-5.4-63.3)	39.1 (-36.5-114.7)	19.5 (-18.3-57.3)
Delirium, dementia and amnestic and other cognitive disorders	9.1 (3.4-14.7)	13.1 (-7.6-33.8)	6.7 (-0.7-14.2)
Depressive disorders	14.1 (11.3-16.8)	27.3 (18.7-36.0)	6.4 (4.7-8.0)
Developmental disorders	23.3 (-4.4-51.1)	76.7 (-274.2-427.6)	12.8 (-4.6-30.2)
Disorders usually diagnosed in infancy, childhood, or adolescence	21.7 (-20.4-63.8)	30.7 (-109.9-171.2)	17.6 (-28.8-64.0)
Eating disorders	24.8 (9.8-39.8)	33.3 (0.7-65.9)	2.8 (-3.2-8.8)
Personality disorders	17.3 (13.7-21.0)	25.2 (17.7-32.7)	10.4 (7.3-13.4)
Schizophrenia and other psychotic disorders	6.1 (4.9-7.2)	9.1 (6.0-12.3)	4.8 (3.8-5.9)
Drug-related disorders	11.2 (9.2-13.2)	16.8 (11.0-22.5)	9.2 (7.2-11.1)

© 2024 Mortier P et al. *JAMA Network Open*.

Other disorders	22.5 (8.9-36.1)	25.6 (-8.9-60.2)	20.0 (-3.8-43.9)
**Duration of hospitalization**			
0-6	17.7 (14.0-21.3)	31.7 (21.0-42.4)	9.8 (7.2-12.4)
7-13	11.1 (8.9-13.4)	21.7 (13.6-29.8)	7.0 (5.3-8.7)
14-20	12.0 (9.1-14.9)	21.3 (12.5-30.0)	7.3 (5.0-9.5)
21-27	7.7 (5.6-9.7)	9.7 (6.0-13.3)	5.9 (3.7-8.1)
28 or more	9.3 (7.4-11.1)	14.4 (10.0-18.9)	5.8 (4.2-7.3)
**Psychiatric hospitalizations in the previous 6 y, No.**			
0	13.1 (11.5-14.7)	23.0 (18.4-27.7)	7.7 (6.5-8.9)
1	8.9 (6.5-11.2)	13.6 (8.0-19.3)	6.0 (4.0-8.0)
2	8.2 (5.4-11.0)	10.3 (5.3-15.3)	6.4 (3.3-9.4)
3	7.7 (4.2-11.2)	12.6 (-1.0-26.3)	5.3 (2.2-8.3)
4	8.2 (3.8-12.7)	35.0 (-56.7-126.6)	4.2 (1.6-6.7)
>=5	14.5 (8.1-21.0)	16.5 (6.4-26.6)	12.7 (4.5-20.8)
**Covariates**			
**Health region**			
Lleida	13.4 (7.8-19.0)	29.6 (-2.1-61.2)	6.9 (3.4-10.4)
Camp de Tarragona	22.0 (12.3-31.6)	33.7 (3.2-64.2)	13.7 (5.7-21.8)
Terres de l'Ebre	10.9 (3.8-18.0)	15.1 (-8.8-38.9)	8.2 (-1.7-18.2)
Girona	7.8 (5.6-9.9)	12.9 (7.0-18.8)	4.9 (3.1-6.6)
Central Catalonia	10.1 (6.5-13.7)	12.9 (6.3-19.4)	7.8 (4.0-11.6)
High Pyrenees and Aran	/[b]	/[b]	/[b]
Barcelona Metropolitan South	13.1 (10.2-16.0)	23.2 (15.0-31.5)	7.4 (5.3-9.5)
Barcelona Metropolitan North	12.5 (9.9-15.1)	25.2 (15.7-34.7)	7.2 (5.4-9.1)
Barcelona City	9.7 (7.8-11.6)	14.0 (9.8-18.2)	6.8 (5.0-8.6)
**Year of discharge**			
2014	9.8 (8.2-11.3)	16.4 (12.3-20.5)	6.0 (4.7-7.2)
2015	11.4 (9.0-13.8)	17.6 (11.7-23.6)	7.8 (5.7-9.8)
2016	11.2 (8.7-13.8)	17.4 (11.2-23.6)	7.3 (5.2-9.5)
2017	15.9 (11.3-20.4)	29.4 (15.2-43.6)	9.1 (5.8-12.3)
2018	17.8 (11.3-24.2)	33.6 (13.4-53.8)	9.2 (5.0-13.5)

a Lethality Index of post-discharge intentional self-harm is defined as the post-discharge self-harm/suicide incidence ratio, with lower index values indicating higher lethality

b Lethality Index could not be calculated due to zero cases of suicide

© 2024 Mortier P et al. *JAMA Network Open*.

eTable 10. Hazard Ratios for Premature Death After Discharge From Psychiatric Hospitalization, Total and Stratified by Sex (n=44267)[a]

	Total			Females			Males		
**Independent variables**	**AHR[b] (95% CI)**	***P* value**	***P* value[c]**	**AHR[b] (95% CI)**	***P* value**	***P* value[c]**	**AHR[b] (95% CI)**	***P* value**	***P* value[c]**
**Sex**									
male vs female	1.73 (1.57-1.90)	<0.001	<0.001	/	/	/	/	/	/
**Age at discharge from index hospitalization, y[d]**									
10-14	0.24 (0.15-0.38)	<0.001	<0.001	0.35 (0.20-0.62)	<0.001	0.002	0.14 (0.06-0.34)	<0.001	<0.001
15-19	0.34 (0.25-0.47)	<0.001	<0.001	0.18 (0.09-0.34)	<0.001	<0.001	0.51 (0.35-0.74)	<0.001	0.002
20-29	0.60 (0.50-0.73)	<0.001	<0.001	0.61 (0.43-0.87)	0.007	0.03	0.64 (0.50-0.81)	<0.001	0.002
30-39	1.06 (0.92-1.22)	0.43	0.57	1.17 (0.92-1.48)	0.20	0.42	1.07 (0.88-1.31)	0.49	0.70
40-49	1.65 (1.46-1.87)	<0.001	<0.001	1.68 (1.37-2.07)	<0.001	<0.001	1.73 (1.44-2.08)	<0.001	<0.001
50-59	2.51 (2.22-2.85)	<0.001	<0.001	2.78 (2.27-3.41)	<0.001	<0.001	2.50 (2.07-3.01)	<0.001	<0.001
60-69	4.57 (3.99-5.23)	<0.001	<0.001	4.83 (3.88-6.00)	<0.001	<0.001	4.66 (3.82-5.68)	<0.001	<0.001
**Socio-economic group[d]**									
contributory annual income < 18000 €/y	1.00 (0.94-1.06)	0.98	0.98	0.99 (0.90-1.10)	0.89	0.89	1.01 (0.94-1.09)	0.76	0.83
contributory annual income > 18000 €/y	0.92 (0.84-1.01)	0.07	0.15	0.89 (0.77-1.04)	0.14	0.31	0.93 (0.83-1.05)	0.24	0.39
socio-economically vulnerable categories	1.09 (1.00-1.17)	0.04	0.10	1.13 (0.99-1.28)	0.06	0.18	1.06 (0.96-1.17)	0.25	0.39
**Diagnosis of intentional self-harm associated with index hospitalization**	1.03 (0.80-1.32)	0.83	0.87	1.13 (0.76-1.67)	0.56	0.70	0.97 (0.70-1.36)	0.88	0.91
**Intentional self-harm after discharge from index psychiatric hospitalization**	1.12 (0.97-1.28)	0.11	0.23	1.03 (0.82-1.28)	0.81	0.89	1.19 (1.00-1.42)	0.05	0.13
**Mental disorders associated with index hospitalization**									
Adjustment disorders	0.93 (0.79-1.09)	0.35	0.52	0.85 (0.64-1.12)	0.26	0.47	0.96 (0.79-1.16)	0.66	0.77
Alcohol-related disorders	1.29 (1.17-1.42)	<0.001	<0.001	1.41 (1.18-1.70)	<0.001	0.002	1.22 (1.09-1.37)	<0.001	0.002
Anxiety disorders	0.84 (0.71-1.00)	0.05	0.12	0.83 (0.62-1.11)	0.21	0.43	0.84 (0.68-1.03)	0.10	0.20
Attention-deficit/hyperactivity disorder	1.08 (0.68-1.71)	0.76	0.81	1.48 (0.60-3.66)	0.39	0.60	1.00 (0.58-1.71)	0.99	0.99
Bipolar disorders	0.76 (0.66-0.88)	<0.001	<0.001	0.75 (0.59-0.96)	0.02	0.07	0.75 (0.62-0.91)	0.003	0.01
Conduct disorder or oppositional defiant disorder	0.89 (0.63-1.27)	0.53	0.64	1.19 (0.68-2.06)	0.54	0.69	0.76 (0.48-1.21)	0.25	0.39
Delirium, dementia and amnestic and other cognitive disorders	2.69 (2.33-3.11)	<0.001	<0.001	2.89 (2.24-3.74)	<0.001	<0.001	2.59 (2.17-3.08)	<0.001	<0.001
Depressive disorders	0.91 (0.81-1.03)	0.12	0.23	0.77 (0.63-0.95)	0.01	0.05	1.03 (0.88-1.19)	0.74	0.82
Developmental disorders	0.89 (0.68-1.15)	0.37	0.54	0.96 (0.62-1.49)	0.86	0.89	0.85 (0.61-1.18)	0.32	0.48
Disorders usually diagnosed in infancy, childhood, or adolescence	0.49 (0.24-0.99)	0.05	0.12	0.42 (0.06-3.02)	0.39	0.60	0.49 (0.23-1.04)	0.06	0.15
Eating disorders	1.15 (0.78-1.69)	0.48	0.61	1.17 (0.76-1.81)	0.48	0.67	1.32 (0.54-3.18)	0.54	0.73
Personality disorders	0.92 (0.82-1.03)	0.16	0.28	0.89 (0.74-1.08)	0.25	0.47	0.94 (0.81-1.09)	0.40	0.59
Schizophrenia and other psychotic disorders	0.75 (0.67-0.84)	<0.001	<0.001	0.79 (0.64-0.98)	0.03	0.10	0.73 (0.63-0.84)	<0.001	<0.001

© 2024 Mortier P et al. *JAMA Network Open*.

Drug-related disorders	0.97 (0.87-1.07)	0.54	0.64	1.23 (1.01-1.49)	0.04	0.13	0.89 (0.79-1.01)	0.07	0.15
Other disorders	0.97 (0.78-1.19)	0.75	0.81	1.03 (0.70-1.51)	0.88	0.89	0.95 (0.73-1.22)	0.68	0.77
**Duration of index hospitalization, d[d]**									
0-6	1.15 (1.06-1.25)	0.001	0.004	1.14 (0.99-1.32)	0.08	0.21	1.17 (1.06-1.29)	0.002	0.009
7-13	0.95 (0.87-1.03)	0.24	0.38	0.84 (0.72-0.98)	0.03	0.10	1.00 (0.91-1.11)	0.97	0.99
14-20	0.98 (0.89-1.07)	0.58	0.66	0.98 (0.84-1.14)	0.79	0.89	0.97 (0.87-1.08)	0.62	0.75
21-27	0.95 (0.86-1.06)	0.39	0.55	1.04 (0.88-1.24)	0.65	0.79	0.91 (0.79-1.04)	0.15	0.28
28 or more	0.98 (0.91-1.06)	0.68	0.75	1.02 (0.90-1.17)	0.75	0.86	0.97 (0.88-1.07)	0.52	0.72
**Psychiatric hospitalizations in the previous 6 y, No.**									
0	1 [Reference]	/	/	1 [Reference]	/	/	1 [Reference]	/	/
1	1.12 (0.99-1.26)	0.06	0.14	1.10 (0.89-1.36)	0.37	0.60	1.13 (0.98-1.31)	0.10	0.20
2	1.31 (1.11-1.54)	0.001	0.004	1.64 (1.26-2.11)	<0.001	0.002	1.15 (0.93-1.42)	0.19	0.33
3	1.22 (0.98-1.51)	0.07	0.15	0.91 (0.59-1.39)	0.67	0.79	1.38 (1.07-1.76)	0.01	0.04
4	1.20 (0.91-1.59)	0.20	0.34	0.77 (0.43-1.38)	0.38	0.60	1.43 (1.04-1.97)	0.03	0.08
>=5	1.48 (1.21-1.80)	<0.001	<0.001	1.75 (1.28-2.40)	<0.001	0.003	1.35 (1.05-1.74)	0.02	0.06
**Covariates**									
**Health region[d]**									
Lleida	0.91 (0.75-1.10)	0.33	0.50	0.85 (0.61-1.18)	0.33	0.59	0.94 (0.74-1.20)	0.61	0.75
Camp de Tarragona	1.10 (0.95-1.28)	0.21	0.34	1.23 (0.95-1.59)	0.11	0.27	1.05 (0.88-1.27)	0.57	0.75
Terres de l'Ebre	0.92 (0.69-1.22)	0.57	0.66	1.29 (0.83-2.01)	0.26	0.47	0.75 (0.52-1.10)	0.14	0.27
Girona	1.11 (0.96-1.28)	0.15	0.27	1.03 (0.80-1.32)	0.84	0.89	1.16 (0.97-1.37)	0.10	0.20
Central Catalonia	1.00 (0.85-1.17)	0.96	0.98	0.91 (0.69-1.21)	0.53	0.69	1.05 (0.86-1.28)	0.64	0.75
High Pyrenees and Aran	0.82 (0.49-1.36)	0.44	0.57	0.74 (0.31-1.78)	0.51	0.68	0.86 (0.46-1.60)	0.63	0.75
Barcelona Metropolitan South	1.16 (1.03-1.30)	0.01	0.04	1.16 (0.95-1.41)	0.14	0.31	1.17 (1.01-1.35)	0.03	0.09
Barcelona Metropolitan North	0.95 (0.85-1.07)	0.43	0.57	0.85 (0.69-1.03)	0.10	0.26	1.02 (0.89-1.17)	0.81	0.86
Barcelona City	1.09 (0.98-1.22)	0.12	0.23	1.08 (0.90-1.31)	0.40	0.60	1.08 (0.95-1.24)	0.24	0.39
**Year of discharge[d]**									
2014	1.10 (1.01-1.20)	0.02	0.07	1.01 (0.88-1.17)	0.87	0.89	1.15 (1.04-1.28)	0.006	0.02
2015	1.13 (1.04-1.23)	0.005	0.02	1.06 (0.91-1.23)	0.46	0.65	1.17 (1.05-1.30)	0.003	0.01
2016	1.24 (1.14-1.36)	<0.001	<0.001	1.30 (1.11-1.52)	0.001	0.005	1.21 (1.08-1.36)	<0.001	0.004
2017	0.96 (0.86-1.08)	0.53	0.64	1.08 (0.89-1.31)	0.42	0.61	0.91 (0.79-1.05)	0.18	0.33
2018	0.67 (0.57-0.79)	<0.001	<0.001	0.66 (0.50-0.88)	0.004	0.02	0.67 (0.55-0.82)	<0.001	<0.001

Abbreviations: CI = confidence interval; AHR = adjusted hazard ratio.

© 2024 Mortier P et al. *JAMA Network Open*.

a All premature death analyses were restricted to the subcohort of 44267 patients with age at discharge 69 years or less, with follow-up time censored at age 70 years. Median (IQR) age at discharge among those with post-discharge premature death was 51.5 (42.1-59.0); median (IQR) age at event was 52.4 (44.0-61.4) years; median (IQR) survival time (i.e., between discharge and event) was 600 (242-1075) days.

b The AHRs were calculated using a single multivariable cause-specific hazard model that included all independent variables shown in the Table.

c After applying false-discovery-rate (Benjamini-Hochberg) correction for multiple testing.

d Effect coding was used to estimate the deviation of risk (hazard) for all separate variable levels from the mean risk (hazard) in the study cohort.

© 2024 Mortier P et al. *JAMA Network Open*.

eTable 11. Hazard Ratios for Suicide After Discharge From Psychiatric Hospitalization, Total and Stratified by Sex (n=49108)[a]

	Total			Females			Males		
**Independent variables**	**AHR[b] (95% CI)**	***P* value**	***P* value[c]**	**AHR[b] (95% CI)**	***P* value**	***P* value[c]**	**AHR[b] (95% CI)**	***P* value**	***P* value[c]**
**Sex**									
male vs female	1.87 (1.51-2.30)	<0.001	<0.001	/	/	/	/	/	/
**Age at discharge from index hospitalization, y[d]**									
10-14	0.50 (0.27-0.93)	0.03	0.10	0.52 (0.22-1.22)	0.14	0.49	0.51 (0.20-1.29)	0.16	0.57
15-19	0.53 (0.32-0.86)	0.01	0.06	0.23 (0.08-0.66)	0.006	0.06	0.84 (0.48-1.46)	0.53	0.87
20-29	1.07 (0.79-1.43)	0.67	0.75	1.22 (0.72-2.05)	0.46	0.76	1.01 (0.70-1.45)	0.97	0.99
30-39	1.26 (0.99-1.60)	0.06	0.15	1.63 (1.11-2.40)	0.01	0.07	1.08 (0.79-1.48)	0.63	0.92
40-49	1.32 (1.06-1.64)	0.01	0.06	1.28 (0.87-1.86)	0.21	0.62	1.31 (1.00-1.73)	0.05	0.26
50-59	1.41 (1.11-1.79)	0.004	0.04	1.89 (1.30-2.76)	<0.001	0.02	1.15 (0.84-1.57)	0.37	0.72
60-69	1.52 (1.13-2.04)	0.005	0.04	1.87 (1.19-2.93)	0.007	0.06	1.30 (0.87-1.94)	0.20	0.60
70 or more	0.99 (0.66-1.49)	0.97	0.97	0.91 (0.47-1.74)	0.77	0.86	1.09 (0.65-1.85)	0.74	0.94
**Socio-economic group[d]**									
contributory annual income < 18000 €/y	0.97 (0.85-1.11)	0.68	0.75	0.95 (0.76-1.18)	0.62	0.79	1.01 (0.84-1.20)	0.95	0.99
contributory annual income > 18000 €/y	1.24 (1.03-1.50)	0.02	0.09	1.09 (0.81-1.48)	0.57	0.79	1.35 (1.06-1.72)	0.01	0.15
socio-economically vulnerable categories	0.83 (0.69-1.00)	0.05	0.13	0.97 (0.73-1.29)	0.82	0.88	0.74 (0.57-0.95)	0.02	0.15
**Diagnosis of intentional self-harm associated with index hospitalization**	1.65 (1.12-2.43)	0.01	0.06	2.50 (1.45-4.32)	<0.001	0.02	1.22 (0.70-2.14)	0.49	0.82
**Intentional self-harm after discharge from index psychiatric hospitalization**	3.09 (2.47-3.87)	<0.001	<0.001	2.83 (1.97-4.05)	<0.001	<0.001	3.29 (2.47-4.40)	<0.001	<0.001
**Mental disorders associated with index hospitalization**									
Adjustment disorders	1.77 (1.31-2.40)	<0.001	0.004	1.55 (0.93-2.57)	0.09	0.38	1.94 (1.32-2.83)	<0.001	0.01
Alcohol-related disorders	1.07 (0.85-1.35)	0.57	0.73	1.13 (0.74-1.74)	0.57	0.79	1.05 (0.79-1.37)	0.75	0.94
Anxiety disorders	1.39 (1.01-1.92)	0.04	0.12	1.60 (0.97-2.63)	0.06	0.33	1.25 (0.82-1.90)	0.29	0.63
Attention-deficit/hyperactivity disorder	1.49 (0.72-3.11)	0.28	0.51	2.36 (0.72-7.76)	0.16	0.50	1.17 (0.46-2.96)	0.74	0.94
Bipolar disorders	1.55 (1.14-2.11)	0.006	0.04	1.94 (1.21-3.09)	0.006	0.06	1.20 (0.78-1.84)	0.42	0.74
Conduct disorder or oppositional defiant disorder	0.79 (0.34-1.82)	0.57	0.73	1.23 (0.37-4.03)	0.73	0.85	0.53 (0.16-1.74)	0.30	0.63
Delirium, dementia and amnestic and other cognitive disorders	0.64 (0.34-1.20)	0.17	0.36	0.64 (0.23-1.81)	0.40	0.72	0.62 (0.28-1.37)	0.24	0.63
Depressive disorders	1.61 (1.23-2.10)	<0.001	0.006	1.12 (0.72-1.74)	0.60	0.79	2.13 (1.52-2.97)	<0.001	<0.001
Developmental disorders	0.45 (0.20-1.02)	0.05	0.14	0.20 (0.03-1.48)	0.12	0.45	0.60 (0.24-1.46)	0.26	0.63
Disorders usually diagnosed in infancy, childhood, or adolescence	0.61 (0.19-1.96)	0.41	0.59	1.61 (0.22-11.91)	0.64	0.79	0.40 (0.10-1.68)	0.21	0.60
Eating disorders	1.51 (0.80-2.84)	0.20	0.41	1.39 (0.65-2.95)	0.40	0.72	2.90 (0.90-9.31)	0.07	0.32
Personality disorders	1.10 (0.87-1.40)	0.43	0.59	1.18 (0.81-1.71)	0.39	0.72	1.01 (0.73-1.39)	0.95	0.99

© 2024 Mortier P et al. *JAMA Network Open*.

Schizophrenia and other psychotic disorders	1.19 (0.91-1.57)	0.20	0.41	1.12 (0.69-1.80)	0.65	0.79	1.26 (0.90-1.76)	0.18	0.58
Drug-related disorders	0.89 (0.70-1.13)	0.35	0.54	1.15 (0.75-1.75)	0.52	0.79	0.82 (0.62-1.09)	0.18	0.58
Other disorders	0.61 (0.34-1.12)	0.11	0.26	0.99 (0.40-2.43)	0.98	0.98	0.47 (0.21-1.06)	0.07	0.32
**Duration of index hospitalization, d[d]**									
0-6	0.96 (0.79-1.17)	0.69	0.75	0.91 (0.66-1.26)	0.57	0.79	1.02 (0.80-1.30)	0.86	0.97
7-13	1.07 (0.89-1.29)	0.45	0.60	0.90 (0.65-1.24)	0.51	0.79	1.18 (0.95-1.48)	0.14	0.53
14-20	0.89 (0.72-1.09)	0.26	0.49	0.81 (0.57-1.15)	0.23	0.62	0.94 (0.73-1.22)	0.65	0.92
21-27	1.19 (0.96-1.48)	0.12	0.28	1.54 (1.11-2.13)	0.009	0.07	0.99 (0.74-1.34)	0.95	0.99
28 or more	0.92 (0.76-1.11)	0.37	0.57	0.99 (0.74-1.32)	0.93	0.94	0.88 (0.70-1.12)	0.32	0.65
**Psychiatric hospitalizations in the previous 6 y, No.**									
0	1 [Reference]	/	/	1 [Reference]	/	/	1 [Reference]	/	/
1	1.17 (0.88-1.55)	0.28	0.51	1.19 (0.75-1.89)	0.46	0.76	1.17 (0.81-1.67)	0.40	0.73
2	1.52 (1.07-2.18)	0.02	0.09	1.98 (1.17-3.36)	0.01	0.07	1.25 (0.76-2.04)	0.38	0.72
3	1.60 (1.01-2.53)	0.04	0.13	1.45 (0.66-3.21)	0.35	0.72	1.76 (1.00-3.09)	0.05	0.26
4	1.83 (1.07-3.13)	0.03	0.10	0.63 (0.15-2.62)	0.53	0.79	2.73 (1.51-4.92)	<0.001	0.01
>=5	1.23 (0.77-1.96)	0.39	0.59	1.63 (0.83-3.20)	0.15	0.50	0.99 (0.51-1.93)	0.98	0.99
**Covariates**									
**Health region[d]**									
Lleida	1.01 (0.70-1.47)	0.95	0.97	0.68 (0.34-1.34)	0.26	0.67	1.27 (0.81-2.00)	0.29	0.63
Camp de Tarragona	0.60 (0.41-0.90)	0.01	0.06	0.77 (0.42-1.42)	0.41	0.72	0.55 (0.33-0.92)	0.02	0.16
Terres de l'Ebre	1.15 (0.66-2.01)	0.61	0.75	1.46 (0.61-3.52)	0.40	0.72	0.99 (0.49-2.03)	0.99	0.99
Girona	1.34 (1.03-1.75)	0.03	0.10	1.26 (0.81-1.94)	0.30	0.72	1.41 (1.01-1.96)	0.04	0.26
Central Catalonia	1.04 (0.76-1.44)	0.80	0.84	1.26 (0.78-2.04)	0.34	0.72	0.90 (0.58-1.39)	0.64	0.92
High Pyrenees and Aran	/[f]	/[f]	/[f]	/[f]	/[f]	/[f]	/[f]	/[f]	/[f]
Barcelona Metropolitan South	1.02 (0.81-1.27)	0.88	0.92	0.96 (0.66-1.38)	0.81	0.88	1.07 (0.81-1.42)	0.63	0.92
Barcelona Metropolitan North	0.90 (0.72-1.12)	0.33	0.54	0.70 (0.48-1.03)	0.07	0.33	1.03 (0.79-1.35)	0.83	0.96
Barcelona City	1.10 (0.90-1.36)	0.35	0.54	1.23 (0.88-1.70)	0.23	0.62	1.03 (0.79-1.35)	0.81	0.96
**Year of discharge[d]**									
2014	1.10 (0.92-1.32)	0.30	0.51	1.06 (0.78-1.43)	0.72	0.85	1.13 (0.91-1.42)	0.27	0.63
2015	0.96 (0.79-1.16)	0.65	0.75	0.98 (0.71-1.35)	0.89	0.93	0.96 (0.75-1.22)	0.72	0.94
2016	1.09 (0.89-1.33)	0.43	0.59	1.16 (0.83-1.62)	0.37	0.72	1.04 (0.80-1.35)	0.75	0.94
2017	0.95 (0.74-1.21)	0.66	0.75	0.90 (0.59-1.37)	0.61	0.79	0.97 (0.71-1.31)	0.82	0.96
2018	0.93 (0.68-1.25)	0.62	0.75	0.93 (0.56-1.55)	0.77	0.86	0.92 (0.63-1.34)	0.65	0.92

© 2024 Mortier P et al. *JAMA Network Open*.

Abbreviations: CI = confidence interval; AHR = adjusted hazard ratio.

a Median (IQR) age at discharge among those with post-discharge death by suicide was 44.7 (35.8-55.7) years; median (IQR) age at event was 46.8 (36.9-56.9) years; median (IQR) survival time (i.e., between discharge and event) was 430 (132-976) days.

b per 100 000 person-years.

c The AHRs were calculated using a single multivariable cause-specific hazard model that included all variables showed in the Table.

d After applying false-discovery-rate (Benjamini-Hochberg) correction for multiple testing.

e Effect coding was used to estimate the deviation of risk (hazard) for all separate variable levels from the mean risk (hazard) in the study cohort.

f AHR could not be estimated due to zero cases of suicide

© 2024 Mortier P et al. *JAMA Network Open*.

eTable 12. Hazard Ratios for Non-Lethal Intentional Self-Harm After Discharge From Psychiatric Hospitalization, Total and Stratified by Sex (n=49108)[a]

	Total			Females			Males		
**Independent variables**	**AHR[b] (95% CI)**	***P* value**	***P* value[c]**	**AHR[b] (95% CI)**	***P* value**	***P* value[c]**	**AHR[b] (95% CI)**	***P* value**	***P* value[c]**
**Sex**									
female vs male	1.47 (1.38-1.56)	<0.001	<0.001	/	/	/	/	/	/
**Age at discharge from index hospitalization, y[d]**									
10-14	1.38 (1.24-1.54)	<0.001	<0.001	1.59 (1.40-1.80)	<0.001	<0.001	0.91 (0.71-1.17)	0.45	0.60
15-19	1.35 (1.23-1.47)	<0.001	<0.001	1.42 (1.28-1.58)	<0.001	<0.001	1.17 (0.98-1.40)	0.08	0.15
20-29	1.15 (1.06-1.25)	<0.001	0.002	1.19 (1.07-1.33)	0.002	0.004	1.10 (0.97-1.25)	0.15	0.25
30-39	1.17 (1.09-1.25)	<0.001	<0.001	1.15 (1.05-1.26)	0.002	0.006	1.21 (1.09-1.35)	<0.001	0.002
40-49	1.18 (1.11-1.25)	<0.001	<0.001	1.18 (1.09-1.28)	<0.001	<0.001	1.21 (1.09-1.34)	<0.001	0.001
50-59	0.93 (0.86-1.00)	0.05	0.06	0.88 (0.80-0.97)	0.01	0.02	1.01 (0.90-1.14)	0.88	0.93
60-69	0.77 (0.69-0.85)	<0.001	<0.001	0.76 (0.66-0.86)	<0.001	<0.001	0.81 (0.67-0.98)	0.03	0.07
70 or more	0.48 (0.42-0.55)	<0.001	<0.001	0.41 (0.34-0.49)	<0.001	<0.001	0.71 (0.56-0.90)	0.004	0.01
**Socio-economic group[d]**									
contributory annual income < 18000 €/y	0.96 (0.92-1.00)	0.08	0.10	0.94 (0.90-1.00)	0.03	0.06	0.99 (0.93-1.06)	0.84	0.92
contributory annual income > 18000 €/y	0.93 (0.87-0.98)	0.01	0.02	0.93 (0.87-1.01)	0.08	0.11	0.89 (0.81-0.99)	0.03	0.07
socio-economically vulnerable categories	1.12 (1.06-1.18)	<0.001	<0.001	1.13 (1.06-1.21)	<0.001	0.001	1.13 (1.03-1.23)	0.007	0.02
**Diagnosis of intentional self-harm associated with index hospitalization**	2.16 (1.95-2.39)	<0.001	<0.001	1.95 (1.73-2.21)	<0.001	<0.001	2.62 (2.20-3.13)	<0.001	<0.001
**Mental disorders associated with index hospitalization**									
Adjustment disorders	1.66 (1.53-1.81)	<0.001	<0.001	1.48 (1.33-1.65)	<0.001	<0.001	1.99 (1.74-2.27)	<0.001	<0.001
Alcohol-related disorders	1.07 (0.99-1.15)	0.08	0.10	1.04 (0.93-1.16)	0.49	0.58	1.07 (0.96-1.19)	0.20	0.30
Anxiety disorders	1.29 (1.18-1.41)	<0.001	<0.001	1.24 (1.10-1.39)	<0.001	<0.001	1.36 (1.18-1.58)	<0.001	<0.001
Attention-deficit/hyperactivity disorder	1.01 (0.83-1.23)	0.92	0.94	0.98 (0.74-1.31)	0.91	0.91	1.19 (0.90-1.56)	0.23	0.33
Bipolar disorders	0.73 (0.65-0.81)	<0.001	<0.001	0.64 (0.55-0.74)	<0.001	<0.001	0.88 (0.74-1.05)	0.14	0.25
Conduct disorder or oppositional defiant disorder	1.00 (0.84-1.18)	0.96	0.96	1.05 (0.86-1.30)	0.62	0.67	0.96 (0.72-1.28)	0.77	0.90
Delirium, dementia and amnestic and other cognitive disorders	0.60 (0.49-0.75)	<0.001	<0.001	0.54 (0.40-0.72)	<0.001	<0.001	0.69 (0.50-0.94)	0.02	0.05
Depressive disorders	1.64 (1.52-1.77)	<0.001	<0.001	1.54 (1.40-1.69)	<0.001	<0.001	1.80 (1.58-2.04)	<0.001	<0.001
Developmental disorders	0.75 (0.63-0.90)	0.002	0.004	0.72 (0.57-0.92)	0.008	0.02	0.81 (0.63-1.05)	0.12	0.21
Disorders usually diagnosed in infancy, childhood, or adolescence	0.67 (0.52-0.87)	0.003	0.005	0.80 (0.55-1.17)	0.25	0.32	0.70 (0.49-1.00)	0.05	0.11
Eating disorders	1.13 (0.98-1.30)	0.09	0.11	1.03 (0.89-1.19)	0.69	0.72	0.81 (0.40-1.63)	0.56	0.68
Personality disorders	1.53 (1.43-1.64)	<0.001	<0.001	1.59 (1.46-1.73)	<0.001	<0.001	1.43 (1.28-1.60)	<0.001	<0.001

© 2024 Mortier P et al. *JAMA Network Open*.

Schizophrenia and other psychotic disorders	0.52 (0.48-0.58)	<0.001	<0.001	0.44 (0.38-0.51)	<0.001	<0.001	0.63 (0.55-0.72)	<0.001	<0.001
Drug-related disorders	0.94 (0.87-1.01)	0.07	0.09	0.89 (0.80-0.99)	0.03	0.05	1.00 (0.90-1.11)	0.94	0.96
Other disorders	1.07 (0.93-1.22)	0.33	0.36	0.94 (0.78-1.13)	0.51	0.59	1.21 (1.00-1.47)	0.05	0.11
**Duration of index hospitalization, d[d]**									
0-6	1.23 (1.17-1.30)	<0.001	<0.001	1.20 (1.12-1.28)	<0.001	<0.001	1.30 (1.20-1.42)	<0.001	<0.001
7-13	1.07 (1.01-1.13)	0.02	0.03	1.02 (0.95-1.10)	0.57	0.64	1.14 (1.04-1.24)	0.004	0.01
14-20	0.97 (0.91-1.04)	0.40	0.42	0.98 (0.91-1.07)	0.69	0.72	0.97 (0.88-1.07)	0.49	0.62
21-27	0.88 (0.82-0.96)	0.002	0.004	0.90 (0.82-1.00)	0.05	0.07	0.86 (0.76-0.98)	0.02	0.05
28 or more	0.88 (0.83-0.94)	<0.001	<0.001	0.92 (0.85-0.99)	0.03	0.06	0.81 (0.74-0.90)	<0.001	<0.001
**Psychiatric hospitalizations in the previous 6 y, No.**									
0	1 [Reference]	/	/	1 [Reference]	/	/	1 [Reference]	/	/
1	1.07 (0.97-1.17)	0.18	0.21	1.09 (0.96-1.24)	0.16	0.22	1.04 (0.90-1.20)	0.62	0.73
2	1.39 (1.23-1.58)	<0.001	<0.001	1.44 (1.22-1.70)	<0.001	<0.001	1.30 (1.06-1.58)	0.01	0.03
3	1.40 (1.19-1.66)	<0.001	<0.001	1.27 (1.01-1.60)	0.04	0.07	1.57 (1.22-2.01)	<0.001	0.002
4	1.77 (1.46-2.14)	<0.001	<0.001	1.63 (1.25-2.11)	<0.001	<0.001	1.95 (1.46-2.61)	<0.001	<0.001
>=5	2.22 (1.94-2.53)	<0.001	<0.001	1.96 (1.64-2.33)	<0.001	<0.001	2.59 (2.11-3.18)	<0.001	<0.001
**Covariates**									
**Health region[d]**									
Lleida	1.06 (0.95-1.19)	0.28	0.31	1.10 (0.95-1.26)	0.21	0.28	1.02 (0.85-1.23)	0.84	0.92
Camp de Tarragona	1.14 (1.03-1.25)	0.01	0.02	1.24 (1.09-1.40)	<0.001	0.002	1.01 (0.86-1.18)	0.92	0.95
Terres de l'Ebre	1.16 (0.97-1.39)	0.11	0.13	1.07 (0.84-1.37)	0.57	0.64	1.27 (0.97-1.66)	0.08	0.15
Girona	0.88 (0.80-0.97)	0.01	0.02	0.89 (0.78-1.01)	0.08	0.11	0.87 (0.74-1.01)	0.07	0.15
Central Catalonia	0.87 (0.78-0.97)	0.01	0.02	0.85 (0.74-0.98)	0.03	0.05	0.88 (0.75-1.05)	0.16	0.25
High Pyrenees and Aran	1.05 (0.79 - 1.38)	0.75	0.78	1.06 (0.75 - 1.50)	0.72	0.74	1.00 (0.64 - 1.58)	0.99	0.99
Barcelona Metropolitan South	1.07 (0.99-1.15)	0.07	0.09	1.06 (0.97-1.16)	0.23	0.29	1.11 (0.98-1.25)	0.09	0.17
Barcelona Metropolitan North	0.91 (0.84-0.97)	0.006	0.01	0.87 (0.80-0.96)	0.004	0.008	0.96 (0.86-1.07)	0.47	0.61
Barcelona City	0.92 (0.86-0.99)	0.03	0.05	0.92 (0.84-1.01)	0.08	0.12	0.94 (0.84-1.05)	0.26	0.38
**Year of discharge[d]**									
2014	0.93 (0.88-0.98)	0.01	0.02	0.96 (0.90-1.03)	0.30	0.36	0.89 (0.81-0.97)	0.01	0.03
2015	0.92 (0.87-0.98)	0.005	0.010	0.87 (0.80-0.94)	<0.001	<0.001	1.01 (0.92-1.10)	0.84	0.92
2016	0.94 (0.88-0.99)	0.03	0.05	0.92 (0.85-1.00)	0.04	0.06	0.97 (0.88-1.07)	0.51	0.63
2017	1.07 (1.00-1.14)	0.04	0.06	1.07 (0.98-1.16)	0.11	0.15	1.08 (0.97-1.20)	0.17	0.26
2018	1.17 (1.08-1.26)	<0.001	<0.001	1.22 (1.11-1.34)	<0.001	<0.001	1.07 (0.94-1.22)	0.31	0.43

© 2024 Mortier P et al. *JAMA Network Open*.

Abbreviations: CI = confidence interval; AHR = adjusted hazard ratio.

a Median (IQR) age at discharge among those with post-discharge non-lethal intentional self-harm was 41.1 (27.1-50.3) years; median (IQR) age at event was 42.1 (28.5-51.6) years; median (IQR) survival time (i.e., between discharge and event) was 314 (IQR=80-752) days.

b per 100 000 person-years.

c The AHRs were calculated using a single multivariable cause-specific hazard model that included all variables showed in the Table.

d After applying false-discovery-rate (Benjamini-Hochberg) correction for multiple testing.

e Effect coding was used to estimate the deviation of risk (hazard) for all separate variable levels from the mean risk (hazard) in the study cohort.

© 2024 Mortier P et al. *JAMA Network Open*.

eTable 13. Hazard Ratios for Premature Death After Discharge From Psychiatric Hospitalization, Total and Stratified by Post-Discharge Follow-Up Time (n=44267)[a]

	Total			7-days[e]			1-month		
**Independent variables**	**AHR[b] (95% CI)**	***P* value**	***P* value[c]**	**AHR[b] (95% CI)**	***P* value**	***P* value[c]**	**AHR[b] (95% CI)**	***P* value**	***P* value[c]**
**Sex**									
male vs female	1.73 (1.57 - 1.90)	<0.001	<0.001	/	/	/	1.98 (1.32 - 2.98)	0.001	0.02
**Age at discharge from index hospitalization, y[d]**									
10-14	0.24 (0.15 - 0.38)	<0.001	<0.001	/	/	/	0.36 (0.06 - 2.06)	0.25	0.60
15-19	0.34 (0.25 - 0.47)	<0.001	<0.001	/	/	/	0.21 (0.04 - 1.17)	0.08	0.33
20-29	0.60 (0.50 - 0.73)	<0.001	<0.001	/	/	/	0.72 (0.33 - 1.55)	0.40	0.72
30-39	1.06 (0.92 - 1.22)	0.43	0.57	/	/	/	1.00 (0.54 - 1.85)	0.99	1.00
40-49	1.65 (1.46 - 1.87)	<0.001	<0.001	/	/	/	1.66 (0.97 - 2.84)	0.06	0.30
50-59	2.51 (2.22 - 2.85)	<0.001	<0.001	/	/	/	2.35 (1.36 - 4.05)	0.002	0.03
60-69	4.57 (3.99 - 5.23)	<0.001	<0.001	/	/	/	4.72 (2.72 - 8.21)	<0.001	<0.001
70 or more	0.00 (0.00 - 0.00)	<0.001	<0.001	/	/	/	0.00 (0.00 - 0.00)	<0.001	<0.001
**Socio-economic group[d]**									
contributory annual income < 18000 €/y	1.00 (0.94 - 1.06)	0.98	0.98	/	/	/	0.85 (0.66 - 1.10)	0.22	0.60
contributory annual income > 18000 €/y	0.92 (0.84 - 1.01)	0.07	0.15	/	/	/	0.89 (0.62 - 1.28)	0.54	0.83
socio-economically vulnerable categories	1.09 (1.00 - 1.17)	0.04	0.10	/	/	/	1.31 (0.96 - 1.80)	0.09	0.35
**Diagnosis of intentional self-harm associated with index hospitalization**	1.03 (0.80 - 1.32)	0.83	0.87	/	/	/	1.48 (0.58 - 3.74)	0.41	0.72
**Intentional self-harm after discharge from index psychiatric hospitalization**	1.12 (0.97 - 1.28)	0.11	0.23	/	/	/	0.57 (0.26 - 1.24)	0.16	0.52
**Mental disorders associated with index hospitalization**									
Adjustment disorders	0.93 (0.79 - 1.09)	0.35	0.52	/	/	/	0.80 (0.41 - 1.56)	0.51	0.80
Alcohol-related disorders	1.29 (1.17 - 1.42)	<0.001	<0.001	/	/	/	0.62 (0.40 - 0.97)	0.04	0.25
Anxiety disorders	0.84 (0.71 - 1.00)	0.05	0.12	/	/	/	1.03 (0.54 - 1.96)	0.92	1.00
Attention-deficit/hyperactivity disorder	1.08 (0.68 - 1.71)	0.76	0.81	/	/	/	/	/	/
Bipolar disorders	0.76 (0.66 - 0.88)	<0.001	<0.001	/	/	/	0.47 (0.23 - 1.00)	0.05	0.28
Conduct disorder or oppositional defiant disorder	0.89 (0.63 - 1.27)	0.53	0.64	/	/	/	0.74 (0.18 - 3.11)	0.69	0.94
Delirium, dementia and amnestic and other cognitive disorders	2.69 (2.33 - 3.11)	<0.001	<0.001	/	/	/	2.53 (1.44 - 4.44)	0.001	0.02
Depressive disorders	0.91 (0.81 - 1.03)	0.12	0.23	/	/	/	0.95 (0.58 - 1.56)	0.85	0.99
Developmental disorders	0.89 (0.68 - 1.15)	0.37	0.54	/	/	/	0.91 (0.29 - 2.91)	0.88	0.99
Disorders usually diagnosed in infancy, childhood, or adolescence	0.49 (0.24 - 0.99)	0.05	0.12	/	/	/	/	/	/
Eating disorders	1.15 (0.78 - 1.69)	0.48	0.61	/	/	/	0.80 (0.11 - 5.97)	0.82	0.99
Personality disorders	0.92 (0.82 - 1.03)	0.16	0.28	/	/	/	1.02 (0.63 - 1.66)	0.93	1.00

© 2024 Mortier P et al. *JAMA Network Open*.

Schizophrenia and other psychotic disorders	0.75 (0.67 - 0.84)	<0.001	<0.001	/	/	/	0.57 (0.34 - 0.97)	0.04	0.25
Drug-related disorders	0.97 (0.87 - 1.07)	0.54	0.64	/	/	/	1.30 (0.83 - 2.04)	0.25	0.60
Other disorders	0.97 (0.78 - 1.19)	0.75	0.81	/	/	/	1.42 (0.69 - 2.94)	0.34	0.71
**Duration of index hospitalization, d[d]**									
0-6	1.15 (1.06 - 1.25)	0.001	0.004	/	/	/	1.44 (1.04 - 2.00)	0.03	0.25
7-13	0.95 (0.87 - 1.03)	0.24	0.38	/	/	/	1.06 (0.73 - 1.53)	0.76	0.96
14-20	0.98 (0.89 - 1.07)	0.58	0.66	/	/	/	0.76 (0.49 - 1.18)	0.22	0.60
21-27	0.95 (0.86 - 1.06)	0.39	0.55	/	/	/	0.87 (0.53 - 1.44)	0.59	0.87
28 or more	0.98 (0.91 - 1.06)	0.68	0.75	/	/	/	0.99 (0.69 - 1.42)	0.96	1.00
**Psychiatric hospitalizations in the previous 6 y, No.**									
0	1 [Reference]	/	/	1 [Reference]	/	/	1 [Reference]	/	/
1	1.12 (0.99 - 1.26)	0.06	0.14	/	/	/	0.35 (0.15 - 0.80)	0.01	0.13
2	1.31 (1.11 - 1.54)	0.001	0.004	/	/	/	0.61 (0.22 - 1.68)	0.33	0.71
3	1.22 (0.98 - 1.51)	0.07	0.15	/	/	/	0.56 (0.13 - 2.30)	0.42	0.72
4	1.20 (0.91 - 1.59)	0.20	0.34	/	/	/	0.49 (0.07 - 3.54)	0.48	0.77
>=5	1.48 (1.21 - 1.80)	<0.001	<0.001	/	/	/	0.79 (0.24 - 2.60)	0.70	0.94
**Covariates**									
**Health region[d]**									
Lleida	0.91 (0.75 - 1.10)	0.33	0.50	/	/	/	0.65 (0.26 - 1.64)	0.36	0.72
Camp de Tarragona	1.10 (0.95 - 1.28)	0.21	0.34	/	/	/	0.95 (0.49 - 1.85)	0.88	0.99
Terres de l'Ebre	0.92 (0.69 - 1.22)	0.57	0.66	/	/	/	0.46 (0.08 - 2.65)	0.38	0.72
Girona	1.11 (0.96 - 1.28)	0.15	0.27	/	/	/	1.12 (0.62 - 2.04)	0.70	0.94
Central Catalonia	1.00 (0.85 - 1.17)	0.96	0.98	/	/	/	0.93 (0.45 - 1.93)	0.85	0.99
High Pyrenees and Aran	0.82 (0.49 - 1.36)	0.44	0.57	/	/	/	2.43 (0.68 - 8.63)	0.17	0.52
Barcelona Metropolitan South	1.16 (1.03 - 1.30)	0.01	0.04	/	/	/	1.41 (0.90 - 2.22)	0.14	0.48
Barcelona Metropolitan North	0.95 (0.85 - 1.07)	0.43	0.57	/	/	/	0.77 (0.46 - 1.28)	0.31	0.70
Barcelona City	1.09 (0.98 - 1.22)	0.12	0.23	/	/	/	1.29 (0.83 - 2.01)	0.25	0.60
**Year of discharge[d]**									
2014	1.10 (1.01 - 1.20)	0.02	0.07	/	/	/	1.07 (0.75 - 1.53)	0.70	0.94
2015	1.13 (1.04 - 1.23)	0.005	0.02	/	/	/	1.33 (0.96 - 1.86)	0.09	0.35
2016	1.24 (1.14 - 1.36)	<0.001	<0.001	/	/	/	1.15 (0.81 - 1.63)	0.44	0.75
2017	0.96 (0.86 - 1.08)	0.53	0.64	/	/	/	0.94 (0.63 - 1.39)	0.75	0.96
2018	0.67 (0.57 - 0.79)	<0.001	<0.001	/	/	/	0.65 (0.41 - 1.02)	0.06	0.30

© 2024 Mortier P et al. *JAMA Network Open*.

Abbreviations: CI = confidence interval; AHR = adjusted hazard ratio.

a All premature death analyses were restricted to the subcohort of 44267 patients with age at discharge 69 years or less, with follow-up time censored at age 70 years. Median (IQR) age at discharge among those with post-discharge premature death was 51.5 (42.1-59.0); median (IQR) age at event was 52.4 (44.0-61.4) years; median (IQR) survival time (i.e., between discharge and event) was 600 (242-1075) days.

b The AHRs were calculated using a single multivariable cause-specific hazard model that included all independent variables shown in the Table.

c After applying false-discovery-rate (Benjamini-Hochberg) correction for multiple testing.

d Effect coding was used to estimate the deviation of risk (hazard) for all separate variable levels from the mean risk (hazard) in the study cohort.

e Results not reported due to non-convergence of models caused by data sparsity (i.e., small frequencies in the outcome, predictor or covariate variables).

© 2024 Mortier P et al. *JAMA Network Open*.

eTable 13. *(continued)*

	3-month			1-year			5-year		
**Independent variables**	**AHR[b] (95% CI)**	***P* value**	***P* value[c]**	**AHR[b] (95% CI)**	***P* value**	***P* value[c]**	**AHR[b] (95% CI)**	***P* value**	***P* value[c]**
**Sex**									
male vs female	1.83 (1.40 - 2.39)	<0.001	<0.001	1.85 (1.57 - 2.17)	<0.001	<0.001	1.75 (1.59 - 1.92)	<0.001	<0.001
**Age at discharge from index hospitalization, y[d]**									
10-14	0.29 (0.08 - 1.02)	0.05	0.16	0.26 (0.12 - 0.57)	<0.001	0.005	0.23 (0.14 - 0.38)	<0.001	<0.001
15-19	0.19 (0.06 - 0.65)	0.008	0.05	0.42 (0.26 - 0.69)	<0.001	0.005	0.34 (0.24 - 0.47)	<0.001	<0.001
20-29	0.73 (0.43 - 1.24)	0.24	0.48	0.65 (0.48 - 0.89)	0.006	0.03	0.62 (0.51 - 0.75)	<0.001	<0.001
30-39	1.27 (0.84 - 1.91)	0.25	0.49	1.17 (0.93 - 1.48)	0.17	0.42	1.06 (0.92 - 1.22)	0.43	0.55
40-49	1.59 (1.09 - 2.31)	0.02	0.07	1.45 (1.18 - 1.79)	<0.001	0.004	1.67 (1.47 - 1.90)	<0.001	<0.001
50-59	2.42 (1.66 - 3.53)	<0.001	<0.001	2.10 (1.70 - 2.59)	<0.001	<0.001	2.53 (2.22 - 2.87)	<0.001	<0.001
60-69	4.97 (3.39 - 7.29)	<0.001	<0.001	3.89 (3.13 - 4.84)	<0.001	<0.001	4.55 (3.96 - 5.23)	<0.001	<0.001
70 or more	0.00 (0.00 - 0.00)	<0.001	<0.001	0.00 (0.00 - 0.00)	<0.001	<0.001	0.00 (0.00 - 0.00)	<0.001	<0.001
**Socio-economic group[d]**									
contributory annual income < 18000 €/y	0.80 (0.68 - 0.94)	0.007	0.05	0.93 (0.84 - 1.03)	0.17	0.42	1.00 (0.94 - 1.07)	0.91	0.95
contributory annual income > 18000 €/y	1.06 (0.85 - 1.33)	0.61	0.78	1.03 (0.89 - 1.19)	0.68	0.81	0.93 (0.85 - 1.02)	0.11	0.22
socio-economically vulnerable categories	1.18 (0.96 - 1.46)	0.12	0.29	1.04 (0.91 - 1.19)	0.55	0.77	1.08 (0.99 - 1.16)	0.07	0.16
**Diagnosis of intentional self-harm associated with index hospitalization**	1.71 (0.96 - 3.06)	0.07	0.19	1.12 (0.74 - 1.70)	0.59	0.78	1.06 (0.82 - 1.37)	0.65	0.69
**Intentional self-harm after discharge from index psychiatric hospitalization**	0.58 (0.34 - 0.97)	0.04	0.12	0.78 (0.59 - 1.03)	0.08	0.25	1.09 (0.94 - 1.25)	0.25	0.40
**Mental disorders associated with index hospitalization**									
Adjustment disorders	0.91 (0.59 - 1.41)	0.69	0.83	0.98 (0.76 - 1.28)	0.90	0.92	0.93 (0.79 - 1.10)	0.39	0.53
Alcohol-related disorders	0.77 (0.58 - 1.03)	0.07	0.19	1.09 (0.92 - 1.29)	0.30	0.61	1.28 (1.16 - 1.41)	<0.001	<0.001
Anxiety disorders	1.10 (0.72 - 1.68)	0.65	0.81	0.89 (0.67 - 1.18)	0.42	0.73	0.85 (0.71 - 1.01)	0.06	0.14
Attention-deficit/hyperactivity disorder	0.42 (0.06 - 3.09)	0.40	0.61	0.60 (0.22 - 1.64)	0.32	0.62	1.02 (0.62 - 1.66)	0.95	0.96
Bipolar disorders	0.62 (0.39 - 0.96)	0.03	0.11	0.68 (0.52 - 0.88)	0.004	0.02	0.76 (0.65 - 0.88)	<0.001	<0.001
Conduct disorder or oppositional defiant disorder	0.68 (0.25 - 1.87)	0.46	0.65	0.73 (0.39 - 1.33)	0.30	0.61	0.84 (0.58 - 1.22)	0.37	0.53
Delirium, dementia and amnestic and other cognitive disorders	3.87 (2.75 - 5.44)	<0.001	<0.001	3.31 (2.66 - 4.12)	<0.001	<0.001	2.70 (2.34 - 3.13)	<0.001	<0.001
Depressive disorders	0.99 (0.72 - 1.37)	0.94	0.94	0.94 (0.77 - 1.15)	0.53	0.77	0.91 (0.80 - 1.03)	0.13	0.25
Developmental disorders	0.78 (0.35 - 1.78)	0.56	0.75	0.83 (0.51 - 1.32)	0.43	0.73	0.88 (0.67 - 1.15)	0.36	0.53
Disorders usually diagnosed in infancy, childhood, or adolescence	0.55 (0.08 - 4.03)	0.56	0.75	0.33 (0.08 - 1.35)	0.12	0.36	0.51 (0.25 - 1.04)	0.06	0.15
Eating disorders	1.11 (0.35 - 3.55)	0.86	0.92	1.08 (0.55 - 2.14)	0.82	0.89	1.18 (0.79 - 1.74)	0.42	0.55
Personality disorders	0.97 (0.70 - 1.35)	0.87	0.92	0.94 (0.77 - 1.15)	0.54	0.77	0.93 (0.82 - 1.04)	0.21	0.35

© 2024 Mortier P et al. *JAMA Network Open*.

Schizophrenia and other psychotic disorders	0.65 (0.46 - 0.91)	0.01	0.07	0.75 (0.62 - 0.92)	0.006	0.03	0.75 (0.67 - 0.85)	<0.001	<0.001
Drug-related disorders	1.40 (1.04 - 1.88)	0.03	0.10	1.05 (0.88 - 1.25)	0.60	0.78	0.96 (0.86 - 1.06)	0.41	0.55
Other disorders	1.30 (0.78 - 2.16)	0.31	0.56	1.13 (0.81 - 1.57)	0.47	0.75	0.95 (0.76 - 1.18)	0.62	0.68
**Duration of index hospitalization, d[d]**									
0-6	1.35 (1.08 - 1.67)	0.007	0.05	1.22 (1.07 - 1.40)	0.004	0.02	1.15 (1.06 - 1.26)	<0.001	0.003
7-13	1.04 (0.82 - 1.31)	0.77	0.89	0.96 (0.83 - 1.11)	0.61	0.78	0.94 (0.86 - 1.03)	0.17	0.30
14-20	0.82 (0.62 - 1.08)	0.15	0.37	0.91 (0.78 - 1.07)	0.25	0.57	0.97 (0.89 - 1.06)	0.51	0.62
21-27	1.02 (0.75 - 1.38)	0.91	0.93	0.96 (0.80 - 1.15)	0.64	0.79	0.97 (0.87 - 1.08)	0.53	0.62
28 or more	0.86 (0.68 - 1.10)	0.23	0.48	0.97 (0.85 - 1.12)	0.69	0.81	0.98 (0.90 - 1.06)	0.65	0.69
**Psychiatric hospitalizations in the previous 6 y, No.**									
0	1 [Reference]	/	/	1 [Reference]	/	/	1 [Reference]	/	/
1	0.58 (0.37 - 0.90)	0.02	0.07	0.92 (0.73 - 1.14)	0.43	0.73	1.12 (0.99 - 1.27)	0.06	0.14
2	1.05 (0.61 - 1.80)	0.87	0.92	1.01 (0.73 - 1.39)	0.97	0.97	1.32 (1.12 - 1.56)	<0.001	0.003
3	0.65 (0.26 - 1.60)	0.35	0.59	0.84 (0.53 - 1.34)	0.47	0.75	1.24 (1.00 - 1.55)	0.05	0.14
4	0.47 (0.11 - 1.89)	0.29	0.53	0.94 (0.53 - 1.69)	0.84	0.90	1.24 (0.93 - 1.65)	0.15	0.28
>=5	0.89 (0.41 - 1.94)	0.77	0.89	0.87 (0.55 - 1.38)	0.56	0.77	1.51 (1.23 - 1.85)	<0.001	<0.001
**Covariates**									
**Health region[d]**									
Lleida	1.20 (0.74 - 1.94)	0.46	0.65	0.95 (0.69 - 1.31)	0.76	0.85	0.91 (0.75 - 1.12)	0.38	0.53
Camp de Tarragona	0.89 (0.56 - 1.41)	0.61	0.78	1.14 (0.89 - 1.46)	0.31	0.61	1.13 (0.97 - 1.32)	0.11	0.22
Terres de l'Ebre	0.62 (0.22 - 1.72)	0.35	0.59	0.92 (0.56 - 1.51)	0.74	0.85	0.91 (0.68 - 1.23)	0.55	0.62
Girona	1.16 (0.78 - 1.73)	0.45	0.65	1.04 (0.81 - 1.32)	0.77	0.85	1.09 (0.95 - 1.27)	0.22	0.36
Central Catalonia	0.96 (0.59 - 1.54)	0.86	0.92	0.81 (0.60 - 1.09)	0.16	0.42	1.00 (0.84 - 1.18)	0.96	0.96
High Pyrenees and Aran	1.07 (0.31 - 3.73)	0.91	0.93	0.95 (0.44 - 2.09)	0.91	0.92	0.79 (0.46 - 1.34)	0.38	0.53
Barcelona Metropolitan South	1.39 (1.02 - 1.90)	0.04	0.12	1.23 (1.02 - 1.49)	0.03	0.10	1.18 (1.05 - 1.33)	0.006	0.02
Barcelona Metropolitan North	0.80 (0.57 - 1.12)	0.19	0.43	0.94 (0.78 - 1.14)	0.52	0.77	0.96 (0.86 - 1.08)	0.51	0.62
Barcelona City	1.15 (0.85 - 1.56)	0.37	0.60	1.09 (0.91 - 1.31)	0.36	0.66	1.08 (0.97 - 1.21)	0.16	0.28
**Year of discharge[d]**									
2014	1.15 (0.91 - 1.45)	0.23	0.48	1.19 (1.04 - 1.36)	0.01	0.04	1.10 (1.01 - 1.19)	0.03	0.08
2015	1.29 (1.03 - 1.62)	0.03	0.10	1.21 (1.05 - 1.38)	0.007	0.03	1.13 (1.04 - 1.23)	0.005	0.02
2016	1.42 (1.14 - 1.77)	0.002	0.02	1.25 (1.09 - 1.43)	0.002	0.01	1.25 (1.14 - 1.37)	<0.001	<0.001
2017	0.89 (0.67 - 1.16)	0.38	0.60	0.91 (0.77 - 1.07)	0.24	0.57	0.97 (0.86 - 1.08)	0.55	0.62
2018	0.54 (0.38 - 0.75)	<0.001	0.003	0.62 (0.51 - 0.75)	<0.001	<0.001	0.67 (0.57 - 0.79)	<0.001	<0.001

© 2024 Mortier P et al. *JAMA Network Open*.

Abbreviations: CI = confidence interval; AHR = adjusted hazard ratio.

a All premature death analyses were restricted to the subcohort of 44267 patients with age at discharge 69 years or less, with follow-up time censored at age 70 years. Median (IQR) age at discharge among those with post-discharge premature death was 51.5 (42.1-59.0); median (IQR) age at event was 52.4 (44.0-61.4) years; median (IQR) survival time (i.e., between discharge and event) was 600 (242-1075) days.

b The AHRs were calculated using a single multivariable cause-specific hazard model that included all independent variables shown in the Table.

c After applying false-discovery-rate (Benjamini-Hochberg) correction for multiple testing.

d Effect coding was used to estimate the deviation of risk (hazard) for all separate variable levels from the mean risk (hazard) in the study cohort.

e Results not reported due to non-convergence of models caused by data sparsity (i.e., small frequencies in the outcome, predictor or covariate variables).

© 2024 Mortier P et al. *JAMA Network Open*.

eTable 14. Hazard Ratios for Suicide After Discharge From Psychiatric Hospitalization, Total and Stratified by Post-Discharge Follow-Up Time (n=49108)[a]

	Total			7-days[e]			1-month[e]		
**Independent variables**	**AHR[b] (95% CI)**	***P* value**	***P* value[c]**	**AHR[b] (95% CI)**	***P* value**	***P* value[c]**	**AHR[b] (95% CI)**	***P* value**	***P* value[c]**
**Sex**									
male vs female	1.87 (1.51 - 2.30)	<0.001	<0.001	/	/	/	/	/	/
**Age at discharge from index hospitalization, y[d]**									
10-14	0.50 (0.27 - 0.93)	0.03	0.10	/	/	/	/	/	/
15-19	0.53 (0.32 - 0.86)	0.01	0.06	/	/	/	/	/	/
20-29	1.07 (0.79 - 1.43)	0.67	0.75	/	/	/	/	/	/
30-39	1.26 (0.99 - 1.60)	0.06	0.15	/	/	/	/	/	/
40-49	1.32 (1.06 - 1.64)	0.01	0.06	/	/	/	/	/	/
50-59	1.41 (1.11 - 1.79)	0.004	0.04	/	/	/	/	/	/
60-69	1.52 (1.13 - 2.04)	0.005	0.04	/	/	/	/	/	/
70 or more	0.99 (0.66 - 1.49)	0.97	0.97	/	/	/	/	/	/
**Socio-economic group[d]**									
contributory annual income < 18000 €/y	0.97 (0.85 - 1.11)	0.68	0.75	/	/	/	/	/	/
contributory annual income > 18000 €/y	1.24 (1.03 - 1.50)	0.02	0.09	/	/	/	/	/	/
socio-economically vulnerable categories	0.83 (0.69 - 1.00)	0.05	0.13	/	/	/	/	/	/
**Diagnosis of intentional self-harm associated with index hospitalization**	1.65 (1.12 - 2.43)	0.01	0.06	/	/	/	/	/	/
**Intentional self-harm after discharge from index psychiatric hospitalization**	3.09 (2.47 - 3.87)	<0.001	<0.001	/	/	/	/	/	/
**Mental disorders associated with index hospitalization**									
Adjustment disorders	1.77 (1.31 - 2.40)	<0.001	0.004	/	/	/	/	/	/
Alcohol-related disorders	1.07 (0.85 - 1.35)	0.57	0.73	/	/	/	/	/	/
Anxiety disorders	1.39 (1.01 - 1.92)	0.04	0.12	/	/	/	/	/	/
Attention-deficit/hyperactivity disorder	1.49 (0.72 - 3.11)	0.28	0.51	/	/	/	/	/	/
Bipolar disorders	1.55 (1.14 - 2.11)	0.006	0.04	/	/	/	/	/	/
Conduct disorder or oppositional defiant disorder	0.79 (0.34 - 1.82)	0.57	0.73	/	/	/	/	/	/
Delirium, dementia and amnestic and other cognitive disorders	0.64 (0.34 - 1.20)	0.17	0.36	/	/	/	/	/	/
Depressive disorders	1.61 (1.23 - 2.10)	<0.001	0.006	/	/	/	/	/	/
Developmental disorders	0.45 (0.20 - 1.02)	0.05	0.14	/	/	/	/	/	/
Disorders usually diagnosed in infancy, childhood, or adolescence	0.61 (0.19 - 1.96)	0.41	0.59	/	/	/	/	/	/
Eating disorders	1.51 (0.80 - 2.84)	0.20	0.41	/	/	/	/	/	/
Personality disorders	1.10 (0.87 - 1.40)	0.43	0.59	/	/	/	/	/	/

© 2024 Mortier P et al. *JAMA Network Open*.

Schizophrenia and other psychotic disorders	1.19 (0.91 - 1.57)	0.20	0.41	/	/	/	/	/	/
Drug-related disorders	0.89 (0.70 - 1.13)	0.35	0.54	/	/	/	/	/	/
Other disorders	0.61 (0.34 - 1.12)	0.11	0.26	/	/	/	/	/	/
**Duration of index hospitalization, d[d]**									
0-6	0.96 (0.79 - 1.17)	0.69	0.75	/	/	/	/	/	/
7-13	1.07 (0.89 - 1.29)	0.45	0.60	/	/	/	/	/	/
14-20	0.89 (0.72 - 1.09)	0.26	0.49	/	/	/	/	/	/
21-27	1.19 (0.96 - 1.48)	0.12	0.28	/	/	/	/	/	/
28 or more	0.92 (0.76 - 1.11)	0.37	0.57	/	/	/	/	/	/
**Psychiatric hospitalizations in the previous 6 y, No.**									
0	1 [Reference]	/	/	1 [Reference]	/	/	1 [Reference]	/	/
1	1.17 (0.88 - 1.55)	0.28	0.51	/	/	/	/	/	/
2	1.52 (1.07 - 2.18)	0.02	0.09	/	/	/	/	/	/
3	1.60 (1.01 - 2.53)	0.04	0.13	/	/	/	/	/	/
4	1.83 (1.07 - 3.13)	0.03	0.10	/	/	/	/	/	/
>=5	1.23 (0.77 - 1.96)	0.39	0.59	/	/	/	/	/	/
**Covariates**									
**Health region[d]**							/	/	/
Lleida	1.01 (0.70 - 1.47)	0.95	0.97	/	/	/	/	/	/
Camp de Tarragona	0.60 (0.41 - 0.90)	0.01	0.06	/	/	/	/	/	/
Terres de l'Ebre	1.15 (0.66 - 2.01)	0.61	0.75	/	/	/	/	/	/
Girona	1.34 (1.03 - 1.75)	0.03	0.10	/	/	/	/	/	/
Central Catalonia	1.04 (0.76 - 1.44)	0.80	0.84	/	/	/	/	/	/
High Pyrenees and Aran	/[f]	/[f]	/[f]	/	/	/	/	/	/
Barcelona Metropolitan South	1.02 (0.81 - 1.27)	0.88	0.92	/	/	/	/	/	/
Barcelona Metropolitan North	0.90 (0.72 - 1.12)	0.33	0.54	/	/	/	/	/	/
Barcelona City	1.10 (0.90 - 1.36)	0.35	0.54	/	/	/	/	/	/
**Year of discharge[d]**									
2014	1.10 (0.92 - 1.32)	0.30	0.51	/	/	/	/	/	/
2015	0.96 (0.79 - 1.16)	0.65	0.75	/	/	/	/	/	/
2016	1.09 (0.89 - 1.33)	0.43	0.59	/	/	/	/	/	/
2017	0.95 (0.74 - 1.21)	0.66	0.75	/	/	/	/	/	/
2018	0.93 (0.68 - 1.25)	0.62	0.75	/	/	/	/	/	/

© 2024 Mortier P et al. *JAMA Network Open*.

Abbreviations: CI = confidence interval; AHR = adjusted hazard ratio.

a Median (IQR) age at discharge among those with post-discharge death by suicide was 44.7 (35.8-55.7) years; median (IQR) age at event was 46.8 (36.9-56.9) years; median (IQR) survival time (i.e., between discharge and event) was 430 (132-976) days.

b The AHRs were calculated using a single multivariable cause-specific hazard model that included all independent variables shown in the Table.

c After applying false-discovery-rate (Benjamini-Hochberg) correction for multiple testing.

d Effect coding was used to estimate the deviation of risk (hazard) for all separate variable levels from the mean risk (hazard) in the study cohort.

e Results not reported due to non-convergence of models caused by data sparsity (i.e., small frequencies in the outcome, predictor or covariate variables).

f AHR could not be estimated due to zero cases of suicide

© 2024 Mortier P et al. *JAMA Network Open*.

eTable 14. ***(continued)***

	3-month[e]			1-year			5-year		
**Independent variables**	**AHR[b] (95% CI)**	***P* value**	***P* value[c]**	**AHR[b] (95% CI)**	***P* value**	***P* value[c]**	**AHR[b] (95% CI)**	***P* value**	***P* value[c]**
**Sex**									
male vs female	/	/	/	2.01 (1.47 - 2.75)	<0.001	<0.001	1.87 (1.51 - 2.31)	<0.001	<0.001
**Age at discharge from index hospitalization, y[d]**									
10-14	/	/	/	0.42 (0.15 - 1.20)	0.11	0.40	0.47 (0.25 - 0.91)	0.02	0.09
15-19	/	/	/	0.55 (0.26 - 1.14)	0.11	0.40	0.52 (0.31 - 0.85)	0.01	0.06
20-29	/	/	/	1.28 (0.84 - 1.96)	0.25	0.56	1.10 (0.82 - 1.48)	0.52	0.68
30-39	/	/	/	1.56 (1.11 - 2.21)	0.01	0.12	1.26 (0.99 - 1.61)	0.07	0.18
40-49	/	/	/	1.25 (0.89 - 1.75)	0.20	0.56	1.29 (1.03 - 1.61)	0.03	0.09
50-59	/	/	/	1.08 (0.73 - 1.58)	0.71	0.84	1.45 (1.15 - 1.84)	0.002	0.02
60-69	/	/	/	1.75 (1.16 - 2.64)	0.008	0.10	1.56 (1.16 - 2.10)	0.003	0.03
70 or more	/	/	/	0.92 (0.52 - 1.64)	0.78	0.87	1.01 (0.67 - 1.52)	0.96	0.98
**Socio-economic group[d]**									
contributory annual income < 18000 €/y	/	/	/	0.93 (0.76 - 1.13)	0.46	0.66	0.99 (0.86 - 1.13)	0.86	0.91
contributory annual income > 18000 €/y	/	/	/	1.37 (1.05 - 1.79)	0.02	0.13	1.23 (1.01 - 1.49)	0.03	0.11
socio-economically vulnerable categories	/	/	/	0.79 (0.59 - 1.04)	0.10	0.40	0.82 (0.68 - 1.00)	0.05	0.14
**Diagnosis of intentional self-harm associated with index hospitalization**	/	/	/	1.41 (0.76 - 2.60)	0.27	0.56	1.70 (1.15 - 2.50)	0.007	0.05
**Intentional self-harm after discharge from index psychiatric hospitalization**	/	/	/	1.93 (1.33 - 2.80)	<0.001	0.01	3.06 (2.43 - 3.84)	<0.001	<0.001
**Mental disorders associated with index hospitalization**									
Adjustment disorders	/	/	/	1.88 (1.22 - 2.90)	0.004	0.08	1.74 (1.28 - 2.36)	<0.001	0.007
Alcohol-related disorders	/	/	/	0.95 (0.67 - 1.34)	0.76	0.86	1.07 (0.85 - 1.35)	0.56	0.68
Anxiety disorders	/	/	/	1.33 (0.84 - 2.13)	0.23	0.56	1.43 (1.04 - 1.96)	0.03	0.09
Attention-deficit/hyperactivity disorder	/	/	/	0.38 (0.05 - 2.76)	0.34	0.63	1.41 (0.65 - 3.07)	0.38	0.57
Bipolar disorders	/	/	/	1.00 (0.61 - 1.65)	1.00	1.00	1.50 (1.09 - 2.05)	0.01	0.06
Conduct disorder or oppositional defiant disorder	/	/	/	0.57 (0.14 - 2.35)	0.43	0.64	0.54 (0.20 - 1.49)	0.24	0.47
Delirium, dementia and amnestic and other cognitive disorders	/	/	/	0.62 (0.26 - 1.46)	0.27	0.56	0.64 (0.34 - 1.21)	0.17	0.35
Depressive disorders	/	/	/	1.61 (1.09 - 2.38)	0.02	0.12	1.61 (1.23 - 2.11)	<0.001	0.007
Developmental disorders	/	/	/	0.34 (0.08 - 1.38)	0.13	0.40	0.48 (0.21 - 1.07)	0.07	0.18
Disorders usually diagnosed in infancy, childhood, or adolescence	/	/	/	0.44 (0.06 - 3.22)	0.42	0.64	0.65 (0.20 - 2.07)	0.47	0.65
Eating disorders	/	/	/	1.70 (0.71 - 4.06)	0.23	0.56	1.57 (0.83 - 2.96)	0.17	0.35
Personality disorders	/	/	/	1.17 (0.82 - 1.67)	0.38	0.64	1.09 (0.85 - 1.39)	0.50	0.66

© 2024 Mortier P et al. *JAMA Network Open*.

Schizophrenia and other psychotic disorders	/	/	/	0.87 (0.58 - 1.32)	0.52	0.70	1.17 (0.89 - 1.55)	0.26	0.47
Drug-related disorders	/	/	/	0.91 (0.64 - 1.29)	0.59	0.75	0.88 (0.69 - 1.12)	0.29	0.51
Other disorders	/	/	/	0.56 (0.23 - 1.37)	0.21	0.56	0.62 (0.34 - 1.14)	0.12	0.30
**Duration of index hospitalization, d[d]**									
0-6	/	/	/	0.99 (0.75 - 1.30)	0.93	0.94	0.98 (0.81 - 1.19)	0.84	0.91
7-13	/	/	/	1.01 (0.77 - 1.33)	0.92	0.94	1.06 (0.88 - 1.27)	0.55	0.68
14-20	/	/	/	0.94 (0.70 - 1.28)	0.71	0.84	0.89 (0.72 - 1.09)	0.26	0.47
21-27	/	/	/	1.15 (0.82 - 1.60)	0.42	0.64	1.18 (0.95 - 1.48)	0.14	0.31
28 or more	/	/	/	0.92 (0.70 - 1.22)	0.57	0.74	0.92 (0.76 - 1.11)	0.37	0.57
**Psychiatric hospitalizations in the previous 6 y, No.**									
0	1 [Reference]	/	/	1 [Reference]	/	/	1 [Reference]	/	/
1	/	/	/	1.27 (0.82 - 1.95)	0.28	0.56	1.18 (0.89 - 1.58)	0.25	0.47
2	/	/	/	1.92 (1.13 - 3.26)	0.02	0.12	1.61 (1.12 - 2.30)	0.009	0.06
3	/	/	/	1.31 (0.57 - 3.03)	0.53	0.70	1.70 (1.07 - 2.69)	0.02	0.09
4	/	/	/	2.18 (0.94 - 5.08)	0.07	0.37	1.70 (0.96 - 3.01)	0.07	0.18
>=5	/	/	/	1.45 (0.68 - 3.08)	0.33	0.63	1.26 (0.78 - 2.04)	0.34	0.54
**Covariates**									
**Health region[d]**									
Lleida	/	/	/	1.33 (0.79 - 2.24)	0.28	0.56	1.03 (0.70 - 1.49)	0.90	0.93
Camp de Tarragona	/	/	/	0.52 (0.28 - 0.98)	0.04	0.25	0.62 (0.42 - 0.91)	0.02	0.08
Terres de l'Ebre	/	/	/	1.06 (0.44 - 2.55)	0.89	0.94	1.18 (0.68 - 2.05)	0.56	0.68
Girona	/	/	/	1.36 (0.91 - 2.04)	0.13	0.40	1.35 (1.03 - 1.76)	0.03	0.09
Central Catalonia	/	/	/	0.79 (0.46 - 1.37)	0.40	0.64	1.00 (0.72 - 1.39)	0.99	0.99
High Pyrenees and Aran	/	/	/	/[f]	/[f]	/[f]	/[f]	/[f]	/[f]
Barcelona Metropolitan South	/	/	/	1.04 (0.74 - 1.46)	0.83	0.90	1.03 (0.82 - 1.29)	0.81	0.90
Barcelona Metropolitan North	/	/	/	0.94 (0.68 - 1.31)	0.72	0.84	0.90 (0.72 - 1.12)	0.33	0.54
Barcelona City	/	/	/	1.29 (0.95 - 1.75)	0.10	0.40	1.08 (0.87 - 1.33)	0.48	0.65
**Year of discharge[d]**									
2014	/	/	/	1.12 (0.87 - 1.46)	0.38	0.64	1.10 (0.92 - 1.31)	0.32	0.53
2015	/	/	/	0.93 (0.70 - 1.25)	0.64	0.80	0.95 (0.79 - 1.16)	0.64	0.74
2016	/	/	/	1.24 (0.94 - 1.63)	0.12	0.40	1.09 (0.89 - 1.33)	0.42	0.60
2017	/	/	/	0.87 (0.62 - 1.21)	0.40	0.64	0.95 (0.74 - 1.22)	0.68	0.77
2018	/	/	/	0.89 (0.62 - 1.26)	0.50	0.70	0.92 (0.68 - 1.25)	0.61	0.72

© 2024 Mortier P et al. *JAMA Network Open*.

Abbreviations: CI = confidence interval; AHR = adjusted hazard ratio.

a Median (IQR) age at discharge among those with post-discharge death by suicide was 44.7 (35.8-55.7) years; median (IQR) age at event was 46.8 (36.9-56.9) years; median (IQR) survival time (i.e., between discharge and event) was 430 (132-976) days.

b The AHRs were calculated using a single multivariable cause-specific hazard model that included all independent variables shown in the Table.

c After applying false-discovery-rate (Benjamini-Hochberg) correction for multiple testing.

d Effect coding was used to estimate the deviation of risk (hazard) for all separate variable levels from the mean risk (hazard) in the study cohort.

e Results not reported due to non-convergence of models caused by data sparsity (i.e., small frequencies in the outcome, predictor or covariate variables).

f AHR could not be estimated due to zero cases of suicide

© 2024 Mortier P et al. *JAMA Network Open*.

eTable 15. Hazard Ratios for Non-Lethal Intentional Self-Harm After Discharge From Psychiatric Hospitalization, Total and Stratified by Post-Discharge Follow-Up Time (n=49108)[a]

	Total			7-days			1-month		
**Independent variables**	**AHR[b] (95% CI)**	***P* value**	***P* value[c]**	**AHR[b] (95% CI)**	***P* value**	***P* value[c]**	**AHR[b] (95% CI)**	***P* value**	***P* value[c]**
**Sex**									
female vs male	1.47 (1.38 - 1.56)	<0.001	<0.001	1.02 (0.80 - 1.28)	0.90	0.98	1.15 (0.98 - 1.35)	0.08	0.22
**Age at discharge from index hospitalization, y[d]**									
10-14	1.38 (1.24 - 1.54)	<0.001	<0.001	0.89 (0.61 - 1.30)	0.55	0.82	0.97 (0.75 - 1.26)	0.81	0.88
15-19	1.35 (1.23 - 1.47)	<0.001	<0.001	0.73 (0.50 - 1.05)	0.09	0.26	0.91 (0.72 - 1.15)	0.44	0.74
20-29	1.15 (1.06 - 1.25)	<0.001	0.002	1.02 (0.75 - 1.41)	0.88	0.98	1.03 (0.83 - 1.28)	0.77	0.88
30-39	1.17 (1.09 - 1.25)	<0.001	<0.001	1.00 (0.76 - 1.32)	0.99	1.00	1.09 (0.91 - 1.31)	0.33	0.63
40-49	1.18 (1.11 - 1.25)	<0.001	<0.001	1.10 (0.87 - 1.39)	0.42	0.78	1.03 (0.87 - 1.20)	0.76	0.88
50-59	0.93 (0.86 - 1.00)	0.05	0.06	1.03 (0.79 - 1.34)	0.82	0.97	0.97 (0.81 - 1.16)	0.73	0.88
60-69	0.77 (0.69 - 0.85)	<0.001	<0.001	1.12 (0.80 - 1.55)	0.52	0.82	1.01 (0.81 - 1.28)	0.90	0.92
70 or more	0.48 (0.42 - 0.55)	<0.001	<0.001	1.19 (0.86 - 1.66)	0.29	0.63	0.99 (0.78 - 1.27)	0.96	0.96
**Socio-economic group[d]**									
contributory annual income < 18000 €/y	0.96 (0.92 - 1.00)	0.08	0.10	1.12 (0.95 - 1.33)	0.17	0.40	1.03 (0.93 - 1.15)	0.56	0.84
contributory annual income > 18000 €/y	0.93 (0.87 - 0.98)	0.01	0.02	1.07 (0.86 - 1.33)	0.54	0.82	1.03 (0.89 - 1.19)	0.67	0.86
socio-economically vulnerable categories	1.12 (1.06 - 1.18)	<0.001	<0.001	0.83 (0.65 - 1.07)	0.14	0.36	0.94 (0.80 - 1.10)	0.42	0.73
**Diagnosis of intentional self-harm associated with index hospitalization**	2.16 (1.95 - 2.39)	<0.001	<0.001	3.76 (2.84 - 4.99)	<0.001	<0.001	3.36 (2.75 - 4.10)	<0.001	<0.001
**Mental disorders associated with index hospitalization**									
Adjustment disorders	1.66 (1.53 - 1.81)	<0.001	<0.001	1.99 (1.49 - 2.64)	<0.001	<0.001	1.94 (1.60 - 2.35)	<0.001	<0.001
Alcohol-related disorders	1.07 (0.99 - 1.15)	0.08	0.10	0.75 (0.56 - 1.02)	0.07	0.23	0.88 (0.72 - 1.08)	0.23	0.50
Anxiety disorders	1.29 (1.18 - 1.41)	<0.001	<0.001	1.44 (1.06 - 1.97)	0.02	0.09	1.49 (1.21 - 1.84)	<0.001	<0.001
Attention-deficit/hyperactivity disorder	1.01 (0.83 - 1.23)	0.92	0.94	0.84 (0.34 - 2.09)	0.71	0.91	0.88 (0.49 - 1.59)	0.67	0.86
Bipolar disorders	0.73 (0.65 - 0.81)	<0.001	<0.001	0.38 (0.21 - 0.70)	0.002	0.01	0.59 (0.41 - 0.83)	0.003	0.01
Conduct disorder or oppositional defiant disorder	1.00 (0.84 - 1.18)	0.96	0.96	0.81 (0.39 - 1.68)	0.57	0.82	0.87 (0.54 - 1.40)	0.56	0.84
Delirium, dementia and amnestic and other cognitive disorders	0.60 (0.49 - 0.75)	<0.001	<0.001	0.14 (0.05 - 0.39)	<0.001	0.001	0.26 (0.15 - 0.47)	<0.001	<0.001
Depressive disorders	1.64 (1.52 - 1.77)	<0.001	<0.001	1.81 (1.37 - 2.38)	<0.001	<0.001	1.81 (1.51 - 2.18)	<0.001	<0.001
Developmental disorders	0.75 (0.63 - 0.90)	0.002	0.004	0.48 (0.18 - 1.29)	0.15	0.36	0.58 (0.32 - 1.05)	0.07	0.21
Disorders usually diagnosed in infancy, childhood, or adolescence	0.67 (0.52 - 0.87)	0.003	0.005	0.58 (0.18 - 1.84)	0.35	0.73	0.41 (0.17 - 1.01)	0.05	0.16
Eating disorders	1.13 (0.98 - 1.30)	0.09	0.11	1.20 (0.68 - 2.11)	0.53	0.82	1.34 (0.94 - 1.90)	0.10	0.27
Personality disorders	1.53 (1.43 - 1.64)	<0.001	<0.001	1.19 (0.91 - 1.54)	0.20	0.45	1.49 (1.26 - 1.77)	<0.001	<0.001
Schizophrenia and other psychotic disorders	0.52 (0.48 - 0.58)	<0.001	<0.001	0.27 (0.16 - 0.44)	<0.001	<0.001	0.35 (0.26 - 0.48)	<0.001	<0.001

© 2024 Mortier P et al. *JAMA Network Open*.

Drug-related disorders	0.94 (0.87 - 1.01)	0.07	0.09	0.69 (0.51 - 0.95)	0.02	0.09	0.69 (0.56 - 0.85)	<0.001	0.002
Other disorders	1.07 (0.93 - 1.22)	0.33	0.36	0.34 (0.15 - 0.77)	0.009	0.05	0.86 (0.60 - 1.24)	0.41	0.73
**Duration of index hospitalization, d[d]**									
0-6	1.23 (1.17 - 1.30)	<0.001	<0.001	2.17 (1.79 - 2.64)	<0.001	<0.001	1.74 (1.53 - 1.98)	<0.001	<0.001
7-13	1.07 (1.01 - 1.13)	0.02	0.03	1.29 (1.02 - 1.63)	0.04	0.13	1.21 (1.04 - 1.41)	0.01	0.05
14-20	0.97 (0.91 - 1.04)	0.40	0.42	0.90 (0.68 - 1.20)	0.48	0.82	0.95 (0.79 - 1.13)	0.54	0.84
21-27	0.88 (0.82 - 0.96)	0.002	0.004	0.70 (0.47 - 1.03)	0.07	0.23	0.78 (0.62 - 0.98)	0.04	0.12
28 or more	0.88 (0.83 - 0.94)	<0.001	<0.001	0.57 (0.41 - 0.78)	<0.001	0.004	0.64 (0.53 - 0.78)	<0.001	<0.001
**Psychiatric hospitalizations in the previous 6 y, No.**									
0	1 [Reference]	/	/	1 [Reference]	/	/	1 [Reference]	/	/
1	1.07 (0.97 - 1.17)	0.18	0.21	0.56 (0.34 - 0.94)	0.03	0.11	0.60 (0.44 - 0.83)	0.002	0.009
2	1.39 (1.23 - 1.58)	<0.001	<0.001	0.86 (0.44 - 1.70)	0.67	0.89	0.90 (0.59 - 1.37)	0.61	0.86
3	1.40 (1.19 - 1.66)	<0.001	<0.001	0.20 (0.03 - 1.47)	0.11	0.31	0.56 (0.26 - 1.19)	0.13	0.32
4	1.77 (1.46 - 2.14)	<0.001	<0.001	1.00 (0.32 - 3.17)	1.00	1.00	0.81 (0.36 - 1.83)	0.61	0.86
>=5	2.22 (1.94 - 2.53)	<0.001	<0.001	0.92 (0.40 - 2.11)	0.84	0.97	0.64 (0.34 - 1.21)	0.17	0.40
**Covariates**									
**Health region[d]**									
Lleida	1.06 (0.95 - 1.19)	0.28	0.31	0.95 (0.64 - 1.40)	0.78	0.94	1.06 (0.81 - 1.37)	0.68	0.86
Camp de Tarragona	1.14 (1.03 - 1.25)	0.01	0.02	0.82 (0.52 - 1.28)	0.38	0.76	0.96 (0.73 - 1.27)	0.79	0.88
Terres de l'Ebre	1.16 (0.97 - 1.39)	0.11	0.13	2.40 (1.43 - 4.01)	<0.001	0.006	2.00 (1.38 - 2.92)	<0.001	0.002
Girona	0.88 (0.80 - 0.97)	0.01	0.02	0.86 (0.59 - 1.24)	0.42	0.78	0.89 (0.69 - 1.14)	0.35	0.65
Central Catalonia	0.87 (0.78 - 0.97)	0.01	0.02	0.67 (0.42 - 1.07)	0.09	0.27	0.66 (0.48 - 0.90)	0.009	0.03
High Pyrenees and Aran	1.05 (0.79 - 1.38)	0.75	0.78	0.85 (0.31 - 2.35)	0.75	0.93	0.84 (0.41 - 1.72)	0.63	0.86
Barcelona Metropolitan South	1.07 (0.99 - 1.15)	0.07	0.09	1.07 (0.82 - 1.40)	0.61	0.86	1.13 (0.94 - 1.35)	0.19	0.42
Barcelona Metropolitan North	0.91 (0.84 - 0.97)	0.006	0.01	0.95 (0.73 - 1.24)	0.72	0.91	0.90 (0.75 - 1.08)	0.27	0.56
Barcelona City	0.92 (0.86 - 0.99)	0.03	0.05	1.08 (0.83 - 1.41)	0.57	0.82	0.99 (0.82 - 1.19)	0.89	0.92
**Year of discharge[d]**									
2014	0.93 (0.88 - 0.98)	0.01	0.02	0.99 (0.80 - 1.22)	0.91	0.98	0.95 (0.82 - 1.10)	0.51	0.83
2015	0.92 (0.87 - 0.98)	0.005	0.010	1.08 (0.87 - 1.33)	0.48	0.82	0.97 (0.84 - 1.13)	0.71	0.88
2016	0.94 (0.88 - 0.99)	0.03	0.05	1.00 (0.81 - 1.24)	1.00	1.00	1.08 (0.94 - 1.24)	0.30	0.60
2017	1.07 (1.00 - 1.14)	0.04	0.06	0.99 (0.80 - 1.23)	0.92	0.98	1.02 (0.88 - 1.18)	0.82	0.88
2018	1.17 (1.08 - 1.26)	<0.001	<0.001	0.95 (0.75 - 1.20)	0.65	0.89	0.99 (0.84 - 1.15)	0.85	0.90

Abbreviations: CI = confidence interval; AHR = adjusted hazard ratio.

© 2024 Mortier P et al. *JAMA Network Open*.

a Median (IQR) age at discharge among those with post-discharge non-lethal intentional self-harm was 41.1 (27.1-50.3) years; median (IQR) age at event was 42.1 (28.5-51.6) years; median (IQR) survival time (i.e., between discharge and event) was 314 (IQR=80-752) days.

b The AHRs were calculated using a single multivariable cause-specific hazard model that included all independent variables shown in the Table.

c After applying false-discovery-rate (Benjamini-Hochberg) correction for multiple testing.

d Effect coding was used to estimate the deviation of risk (hazard) for all separate variable levels from the mean risk (hazard) in the study cohort.

© 2024 Mortier P et al. *JAMA Network Open*.

eTable 15. *(continued)*

	3-month			1-year			5-year		
**Independent variables**	**AHR[b] (95% CI)**	***P* value**	***P* value[c]**	**AHR[b] (95% CI)**	***P* value**	***P* value[c]**	**AHR[b] (95% CI)**	***P* value**	***P* value[c]**
**Sex**									
female vs male	1.30 (1.15 - 1.47)	<0.001	<0.001	1.49 (1.37 - 1.63)	<0.001	<0.001	1.47 (1.38 - 1.57)	<0.001	<0.001
**Age at discharge from index hospitalization, y[d]**									
10-14	1.15 (0.95 - 1.40)	0.16	0.32	1.32 (1.15 - 1.52)	<0.001	<0.001	1.39 (1.25 - 1.55)	<0.001	<0.001
15-19	1.17 (0.99 - 1.38)	0.07	0.16	1.36 (1.21 - 1.53)	<0.001	<0.001	1.36 (1.24 - 1.49)	<0.001	<0.001
20-29	1.09 (0.93 - 1.28)	0.28	0.47	1.14 (1.02 - 1.27)	0.02	0.05	1.14 (1.05 - 1.24)	0.003	0.005
30-39	1.05 (0.92 - 1.21)	0.45	0.62	1.12 (1.01 - 1.23)	0.02	0.05	1.17 (1.09 - 1.25)	<0.001	<0.001
40-49	1.08 (0.96 - 1.22)	0.20	0.39	1.11 (1.02 - 1.21)	0.02	0.04	1.18 (1.11 - 1.26)	<0.001	<0.001
50-59	0.95 (0.83 - 1.10)	0.50	0.66	0.92 (0.83 - 1.02)	0.11	0.17	0.92 (0.86 - 1.00)	0.04	0.05
60-69	0.90 (0.75 - 1.08)	0.26	0.46	0.82 (0.72 - 0.94)	0.005	0.01	0.77 (0.69 - 0.85)	<0.001	<0.001
70 or more	0.70 (0.56 - 0.86)	<0.001	0.004	0.52 (0.44 - 0.62)	<0.001	<0.001	0.47 (0.41 - 0.54)	<0.001	<0.001
**Socio-economic group[d]**									
contributory annual income < 18000 €/y	1.02 (0.94 - 1.11)	0.61	0.75	1.01 (0.96 - 1.07)	0.65	0.70	0.97 (0.93 - 1.01)	0.12	0.14
contributory annual income > 18000 €/y	1.01 (0.90 - 1.13)	0.91	0.97	0.97 (0.90 - 1.05)	0.46	0.55	0.92 (0.87 - 0.98)	0.009	0.02
socio-economically vulnerable categories	0.97 (0.87 - 1.09)	0.64	0.75	1.02 (0.94 - 1.10)	0.68	0.72	1.12 (1.06 - 1.18)	<0.001	<0.001
**Diagnosis of intentional self-harm associated with index hospitalization**	3.07 (2.62 - 3.60)	<0.001	<0.001	2.42 (2.14 - 2.74)	<0.001	<0.001	2.19 (1.98 - 2.42)	<0.001	<0.001
**Mental disorders associated with index hospitalization**									
Adjustment disorders	1.92 (1.65 - 2.23)	<0.001	<0.001	1.85 (1.66 - 2.06)	<0.001	<0.001	1.66 (1.53 - 1.80)	<0.001	<0.001
Alcohol-related disorders	0.92 (0.79 - 1.07)	0.27	0.46	0.96 (0.86 - 1.06)	0.42	0.51	1.07 (0.99 - 1.15)	0.10	0.12
Anxiety disorders	1.43 (1.21 - 1.68)	<0.001	<0.001	1.35 (1.20 - 1.52)	<0.001	<0.001	1.29 (1.18 - 1.41)	<0.001	<0.001
Attention-deficit/hyperactivity disorder	0.84 (0.54 - 1.30)	0.43	0.62	0.89 (0.67 - 1.18)	0.41	0.51	1.02 (0.83 - 1.24)	0.88	0.88
Bipolar disorders	0.72 (0.56 - 0.91)	0.007	0.03	0.73 (0.62 - 0.85)	<0.001	<0.001	0.74 (0.66 - 0.83)	<0.001	<0.001
Conduct disorder or oppositional defiant disorder	0.91 (0.65 - 1.30)	0.61	0.75	0.92 (0.73 - 1.16)	0.48	0.55	0.97 (0.82 - 1.15)	0.74	0.77
Delirium, dementia and amnestic and other cognitive disorders	0.37 (0.24 - 0.58)	<0.001	<0.001	0.49 (0.36 - 0.66)	<0.001	<0.001	0.62 (0.50 - 0.76)	<0.001	<0.001
Depressive disorders	1.86 (1.62 - 2.15)	<0.001	<0.001	1.80 (1.63 - 1.99)	<0.001	<0.001	1.65 (1.53 - 1.78)	<0.001	<0.001
Developmental disorders	0.68 (0.45 - 1.02)	0.06	0.16	0.80 (0.63 - 1.03)	0.08	0.13	0.76 (0.64 - 0.91)	0.003	0.005
Disorders usually diagnosed in infancy, childhood, or adolescence	0.52 (0.29 - 0.94)	0.03	0.08	0.74 (0.53 - 1.02)	0.07	0.12	0.66 (0.51 - 0.86)	0.002	0.005
Eating disorders	1.38 (1.07 - 1.78)	0.01	0.04	1.23 (1.03 - 1.48)	0.02	0.05	1.13 (0.98 - 1.31)	0.08	0.10
Personality disorders	1.63 (1.44 - 1.86)	<0.001	<0.001	1.57 (1.43 - 1.71)	<0.001	<0.001	1.53 (1.43 - 1.64)	<0.001	<0.001
Schizophrenia and other psychotic disorders	0.44 (0.35 - 0.54)	<0.001	<0.001	0.46 (0.40 - 0.53)	<0.001	<0.001	0.53 (0.48 - 0.58)	<0.001	<0.001

© 2024 Mortier P et al. *JAMA Network Open*.

Drug-related disorders	0.77 (0.66 - 0.89)	<0.001	0.003	0.85 (0.76 - 0.94)	0.001	0.005	0.93 (0.87 - 1.01)	0.07	0.09
Other disorders	1.00 (0.77 - 1.30)	0.99	0.99	1.00 (0.83 - 1.20)	0.96	0.96	1.08 (0.94 - 1.23)	0.29	0.32
**Duration of index hospitalization, d[d]**									
0-6	1.44 (1.30 - 1.59)	<0.001	<0.001	1.25 (1.17 - 1.34)	<0.001	<0.001	1.24 (1.18 - 1.31)	<0.001	<0.001
7-13	1.17 (1.05 - 1.31)	0.006	0.02	1.11 (1.03 - 1.20)	0.009	0.02	1.08 (1.02 - 1.14)	0.01	0.02
14-20	0.97 (0.85 - 1.10)	0.63	0.75	0.99 (0.91 - 1.08)	0.80	0.83	0.98 (0.92 - 1.04)	0.51	0.54
21-27	0.86 (0.73 - 1.02)	0.08	0.19	0.86 (0.77 - 0.96)	0.008	0.02	0.87 (0.80 - 0.94)	<0.001	<0.001
28 or more	0.71 (0.62 - 0.81)	<0.001	<0.001	0.85 (0.78 - 0.92)	<0.001	<0.001	0.88 (0.83 - 0.94)	<0.001	<0.001
**Psychiatric hospitalizations in the previous 6 y, No.**									
0	1 [Reference]	/	/	1 [Reference]	/	/	1 [Reference]	/	/
1	0.71 (0.57 - 0.89)	0.003	0.01	0.94 (0.82 - 1.09)	0.42	0.51	1.06 (0.96 - 1.16)	0.26	0.29
2	0.88 (0.64 - 1.22)	0.45	0.62	1.10 (0.90 - 1.34)	0.37	0.48	1.39 (1.22 - 1.58)	<0.001	<0.001
3	0.91 (0.59 - 1.42)	0.69	0.78	1.20 (0.92 - 1.57)	0.17	0.25	1.41 (1.19 - 1.67)	<0.001	<0.001
4	1.06 (0.62 - 1.81)	0.83	0.90	1.34 (0.97 - 1.86)	0.08	0.13	1.78 (1.46 - 2.16)	<0.001	<0.001
>=5	0.92 (0.61 - 1.38)	0.68	0.78	1.49 (1.19 - 1.87)	<0.001	0.002	2.24 (1.96 - 2.57)	<0.001	<0.001
**Covariates**									
**Health region[d]**									
Lleida	1.00 (0.81 - 1.24)	0.98	0.99	1.05 (0.91 - 1.22)	0.49	0.56	1.06 (0.95 - 1.19)	0.30	0.32
Camp de Tarragona	0.91 (0.74 - 1.13)	0.39	0.58	1.01 (0.87 - 1.16)	0.93	0.95	1.14 (1.03 - 1.26)	0.01	0.02
Terres de l'Ebre	1.52 (1.09 - 2.10)	0.01	0.04	1.23 (0.96 - 1.57)	0.10	0.15	1.16 (0.97 - 1.40)	0.10	0.12
Girona	0.95 (0.79 - 1.14)	0.59	0.75	0.87 (0.76 - 1.00)	0.05	0.08	0.88 (0.80 - 0.98)	0.01	0.02
Central Catalonia	0.78 (0.63 - 0.97)	0.03	0.08	0.81 (0.69 - 0.94)	0.006	0.02	0.86 (0.77 - 0.96)	0.007	0.01
High Pyrenees and Aran	1.02 (0.61 - 1.69)	0.95	0.98	1.11 (0.78 - 1.58)	0.55	0.61	1.04 (0.79 - 1.37)	0.80	0.81
Barcelona Metropolitan South	1.13 (0.98 - 1.29)	0.09	0.19	1.08 (0.98 - 1.19)	0.12	0.18	1.07 (1.00 - 1.16)	0.05	0.07
Barcelona Metropolitan North	0.91 (0.79 - 1.04)	0.16	0.32	0.96 (0.87 - 1.05)	0.36	0.48	0.91 (0.85 - 0.98)	0.009	0.02
Barcelona City	0.94 (0.82 - 1.08)	0.38	0.58	0.95 (0.86 - 1.05)	0.30	0.41	0.92 (0.86 - 0.99)	0.04	0.05
**Year of discharge[d]**									
2014	0.91 (0.81 - 1.02)	0.10	0.22	0.93 (0.87 - 1.01)	0.08	0.13	0.93 (0.88 - 0.98)	0.010	0.02
2015	0.94 (0.84 - 1.06)	0.31	0.49	0.92 (0.85 - 1.00)	0.04	0.08	0.92 (0.87 - 0.98)	0.006	0.01
2016	1.02 (0.91 - 1.14)	0.72	0.79	0.95 (0.88 - 1.03)	0.24	0.34	0.94 (0.88 - 0.99)	0.03	0.05
2017	1.06 (0.95 - 1.19)	0.30	0.49	1.09 (1.00 - 1.17)	0.04	0.08	1.07 (1.00 - 1.14)	0.04	0.05
2018	1.08 (0.95 - 1.21)	0.23	0.43	1.12 (1.03 - 1.22)	0.008	0.02	1.17 (1.08 - 1.26)	<0.001	<0.001

Abbreviations: CI = confidence interval; AHR = adjusted hazard ratio.

© 2024 Mortier P et al. *JAMA Network Open*.

a Median (IQR) age at discharge among those with intentional self-harm following discharge = 41.1 years (IQR = 23.1); median (IQR) age at event = 42.1 years (IQR = 23.2); median (IQR) survival time (i.e., between discharge and event) = 314 days (IQR = 672).

b The AHRs were calculated using a single multivariable cause-specific hazard model that included all independent variables shown in the Table.

c After applying false-discovery-rate (Benjamini-Hochberg) correction for multiple testing.

d Effect coding was used to estimate the deviation of risk (hazard) for all separate variable levels from the mean risk (hazard) in the study cohort.

© 2024 Mortier P et al. *JAMA Network Open*.
