# Supplementary material for: Premature Death, Suicide, and Nonlethal Intentional Self-Harm After Psychiatric Discharge
Source: JAMA Netw Open. 2024 Jun 26;7(6):e2417131. doi: 10.1001/jamanetworkopen.2024.17131 (PMC11208976; doi:10.1001/jamanetworkopen.2024.17131)
Supplement: Supplement 2. — Data Sharing Statement [file jamanetwopen-e2417131-s002.pdf]

Data Sharing Statement

Mortier. Premature Death, Suicide, and Nonlethal Intentional Self-Harm After Psychiatric Discharge. *JAMA Netw Open*. Published June 26, 2024.
doi:10.1001/jamanetworkopen.2024.17131

Data
Data available: Yes
Data types: Deidentified participant data, Data dictionary
How to access data: pmortier@researchmar.net
When available: With publication

Supporting Documents
Document types: Statistical/analytic code
How to access documents: pmortier@researchmar.net
When available: With publication

Additional Information
Who can access the data: researchers whose proposed use of the data has been approved
Types of analyses: for the purpose of replicating the analysis and findings as reported in this paper
Mechanisms of data availability: The anonymized registry data as well as the study protocol, statistical analysis plan and data dictionaries used for this study are available as of publication and upon reasonable request from the corresponding authors (Philippe Mortier, pmortier@researchmar.net; Gemma Vilagut, gvilagut@researchmar.net) as long as the main objective of the data-sharing request is replicating the analysis and findings as reported in this paper (without investigator support) after approval of a proposal and with a signed data access agreement.
